# Sex is a defining feature of neuroimaging phenotypes in major brain disorders

**DOI:** 10.1002/hbm.25438

**Published:** 2021-05-05

**Authors:** Lauren E. Salminen, Meral A. Tubi, Joanna Bright, Sophia I. Thomopoulos, Alyssa Wieand, Paul M. Thompson

**Affiliations:** ^1^ Imaging Genetics Center Mark and Mary Stevens Neuroimaging and Informatics Institute, Keck School of Medicine of USC Marina del Rey California USA

**Keywords:** diffusion MRI, ENIGMA, female, gender, male, neuroimaging, neurology, psychiatry, sex, structural MRI

## Abstract

Sex is a biological variable that contributes to individual variability in brain structure and behavior. Neuroimaging studies of population‐based samples have identified normative differences in brain structure between males and females, many of which are exacerbated in psychiatric and neurological conditions. Still, sex differences in MRI outcomes are understudied, particularly in clinical samples with known sex differences in disease risk, prevalence, and expression of clinical symptoms. Here we review the existing literature on sex differences in adult brain structure in normative samples and in 14 distinct psychiatric and neurological disorders. We discuss commonalities and sources of variance in study designs, analysis procedures, disease subtype effects, and the impact of these factors on MRI interpretation. Lastly, we identify key problems in the neuroimaging literature on sex differences and offer potential recommendations to address current barriers and optimize rigor and reproducibility. In particular, we emphasize the importance of large‐scale neuroimaging initiatives such as the Enhancing NeuroImaging Genetics through Meta‐Analyses consortium, the UK Biobank, Human Connectome Project, and others to provide unprecedented power to evaluate sex‐specific phenotypes in major brain diseases.

## Graphical abstract

Here, we review the human neuroimaging literature examining sex effects on adult brain structure using structural and diffusion MRI. We discuss normative sex differences based on population‐based studies as well as sex differences in 14 major brain diseases. Finally, we identify key barriers to advancing the science on neuroimaging sex effects and offer recommendations to mitigate these challenges, particularly through large‐scale neuroimaging.

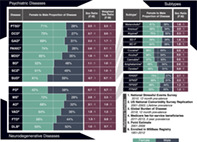

## 1 INTRODUCTION

For many decades, the majority of biomedical knowledge was based on studies of males, leading to major disparities in our understanding of disease etiology, symptom presentation, treatment strategy, and clinical response in females. In 1993, the National Institutes of Health (NIH) in the United States implemented the Revitalization Act (https://orwh.od.nih.gov/resources/pdf/NIH-Revitalization-Act-1993.pdf), which mandated that females must be included in NIH‐funded clinical trials. Unfortunately, sex biases in findings from basic and preclinical research persisted in both human and animal studies. A 2009 study of sex biases in animal research revealed that 80% of all animal studies examined male rodents only, across eight different scientific fields (Beery & Zucker, 2011), with the strongest male biases in neuroscience and pharmacology. Male animals still dominate the biomedical animal literature, particularly in cardiovascular research—a field with known sex differences in health risks, symptom presentation, and treatment response (Ramirez et al., 2017).

Sex disparities in human biomedical research have begun to be addressed in recent years, but they are still understudied. A recent bibliometric analysis of over 11 million papers (Sugimoto, Ahn, Smith, Macaluso, & Larivière, 2019) outlined the severity of sex biases across various fields of medicine, with the vast majority of studies neglecting to report sex characteristics of the sample. Of the disciplines studied, psychiatric and neurological studies reported sex in approximately 80% and 65% of research papers, respectively. The poorest sex reporting came from pharmacology studies, where only 24% of papers disclosed sex characteristics. More disturbingly, greater sex reporting was found in publications in lower impact journals over time, meaning lower visibility for papers that may adequately address sex effects (Sugimoto et al., 2019). To address these persisting biases, the NIH mandated that all grant proposals must address sex as a biological variable (NOT‐OD‐15‐102, http://grants.nih.gov/grants/guide/notice-files/NOT-OD-15-102.html), even for basic and preclinical research. NIH investigators are now required to provide a detailed plan to analyze equal numbers of males and females, or provide sufficient justification if sex distributions would be unequal (Clayton, 2018). Early reports suggest this mandate has improved sex reporting and awareness of its importance in clinical and preclinical studies (Lee, 2018; Zucker & Beery, 2019), but decades of biased work still dominate the literature and continue to be published. For example, a systematic review of 1,827 neuroscience papers published in 2017 (25% human studies) showed that 44% of studies included both males and females but did not consider sex as an experimental variable,^1^ 26% were male‐only, 16% did not report sex, 8% included males and females and did consider sex as an experimental variable, 5% were female‐only, and 1% were hermaphrodites (Mamlouk, Dorris, Barrett, & Meitzen, 2020). These numbers indicate that only 8% of studies adhered to the NIH mandate implemented 1 year prior (~57% were NIH‐funded) (Mamlouk et al., 2020).

Previous preclinical studies justified sex imbalances by claiming that females introduced too much variability into research designs due to hormonal fluctuations along the oestrous cycle (Sugimoto et al., 2019; Wang, ; Zucker & Beery, 2010). However, there is considerable evidence from animal studies showing that variability in behavioral, biological, and molecular end points is consistent between females and males (Sugimoto et al., 2019). Further, a meta‐analysis of 293 rodent studies revealed greater variability in males than females on indices of hormones, metabolism, and morphological traits (Prendergast, Onishi, & Zucker, 2014). The female reproductive cycle also has been used to justify explicit recruitment biases against women in clinical research. For example, pregnant women were considered a vulnerable population that was “protected” (i.e., excluded) from clinical research until the U.S. Department of Health and Human Services (HHS) amended the Federal Policy for the Protection of Human Subjects (“Common Rule”) in 2018 https://www.hhs.gov/ohrp/regulations-and-policy/regulations/45-cfr-46/index.html (Biggio Jr, 2020). The amended policy (implemented in 2019) was a response to the U.S. Task Force on Research Specific to Pregnant Women and Lactating Women, which stated that pregnant women are fully capable of making medical decisions for themselves and their fetus, and that the term “vulnerable” restrained the right to autonomy (Costantine, Landon, & Saade, 2020; Heyrana, Byers, & Stratton, 2018). Research exclusion of pregnant women also led to gross knowledge gaps in treatments and interventions that can be safely administered during pregnancy. While it is too soon to tell whether the new amendment will reduce explicit research biases against pregnant women, the overwhelming exclusion of pregnant women in studies of COVID‐19 suggest that these biases are still in play (Costantine et al., 2020). Finally, implicit sex biases are also well‐documented in healthcare and clinical research and contribute to lower participation of women in clinical trials (Chadwick & Baruah, 2020; Chapman, Kaatz, & Carnes, 2013; Daugherty et al., 2017; Hansen et al., 2019; Kannan et al., 2019; Salles et al., 2019).

The current state of knowledge on sex differences in human brain structure is sparse given the established sex differences in disease prevalence rates, age of onset, and symptom patterns for many psychiatric and neurodegenerative conditions. In this review, we will illuminate these gaps in the human neuroimaging literature and discuss commonly used methods and design strategies that miss opportunities to sufficiently address the role of sex in brain health and disease. To provide mechanistic context for the purported sex differences in various brain disorders and conditions, we first provide a brief overview of the function and trajectory of the primary sex hormones and their associations with neuroimaging indices. We then discuss normative sex differences in brain structure and function from population‐based samples, as these differences are preserved in many clinical conditions and should not be interpreted as an outcome of disease. We chose to review both psychiatric and neurodegenerative diseases, as many psychiatric conditions are risk factors for neurodegenerative diseases and dementia later in life (Ahearn et al., 2020; Almeida, Hankey, Yeap, Golledge, & Flicker, 2017; Diniz et al., 2017; Gimson, Schlosser, Huntley, & Marchant, 2018; Kørner, Lopez, Lauritzen, Andersen, & Kessing, 2009; Kuring, Mathias, & Ward, 2020; Mrabet Khiari et al., 2011; Ribe et al., 2015; Singh‐Manoux et al., 2017; Truelsen et al., 2002; Yaffe et al., 2010; Zilkens, Bruce, Duke, Spilsbury, & Semmens, 2014), and are associated with abnormalities in similar neuroimaging measures. Importantly, psychiatric disorders typically have an earlier age of onset than neurodegenerative conditions, with peak prevalences and symptom severities during the critical decades (ages 20–40) when neurodegenerative pathologies are seeded (Jones, 2013; Kessler et al., 2007; Zilkens et al., 2014). Thus, understanding the role of sex on psychiatric neuroimaging phenotypes may inform etiologic mechanisms of neurodegenerative disease and dementia, and help in the development of novel interventions.

Our literature review was conducted using a combination of search terms in PubMed, Google Scholar, and *bioRxiv*. Search terms to query information about sex included “sex,” “gender,” “males,” “females,” “men,” and “women” in conjunction with comparison terms such as “differences,” “biases,” “disparities,” and “confounds.” Additional phrases included “sex‐specific,” “sex‐by‐age interaction,” “sex‐by‐diagnosis interaction,” “sex covariate,” “adjusting for sex,” “nuisance covariate,” “sexually dimorphic,” “biological sex,” and “genetic sex.” We prioritized the most recent publications first and gradually expanded our search in 2‐year increments; all years were searched if terms yielded no hits in the last decade. As this is not a systematic review and our paper covers 14 distinct neurological and psychiatric conditions (in addition to normative studies), it was not possible to account for every published MRI study on sex effects or every imaging modality within these published studies. Instead, we prioritized large‐scale studies from biobanks (e.g., UK Biobank), consortia (e.g., ENIGMA), and systematic reviews that focused on traditional structural MRI (e.g., volumetrics, thickness, surface area) and diffusion MRI (e.g., diffusion tensor imaging [DTI] scalar metrics) outcomes when available. Small‐scale studies were reviewed when large studies of sex effects had not been conducted, were inconclusive, or contradicted other work. For conditions with a considerable literature on sex effects on neuroimaging (e.g., multiple sclerosis [MS], Alzheimer's disease [AD]), we prioritized studies that contributed to key themes or findings related to sex and neuroimaging. A summary of the studies reviewed is provided in Table S1 in the Supplementary Material. Although many of the conditions in this review may have developmental origins in childhood and adolescence, this literature is highly complex and has been reviewed in detail in other work (Deak et al., 2015; Earls, 1987; Schwarz & Bilbo, 2012). As such, we elected to focus on sex differences in the adult brain to adhere to page constraints and avoid an overly exhaustive and redundant review. Finally, we specifically use the term “sex” to refer to biological differences between males and females, rather than socially constructed roles that vary across time and cultures (i.e., “gender”). Although sex and gender continuously interact to influence the human healthspan, there is limited empirical data that demonstrates these interactive effects on brain structure in the context of major brain diseases. Addressing these research gaps is a central goal of the newly formed ENIGMA Gender Studies and Transgender Working Groups.

## 2 ROLE OF PRIMARY SEX HORMONES IN THE BRAIN

### 2.1 Developmental influences and lifespan trajectories

In humans, the impact of sex differences on health and disease begins as early as 50 days postconception when sex is determined through a cascade of genetic interactions beginning with the sex‐determining region of the Y chromosome (*SRY* gene, chromosome 9) (Mamsen et al., 2017). When SRY transcripts are present, they initiate sexual differentiation of bipotential gonads by activating the SRY‐box 9 gene (*SOX9)* approximately 50 days postconception (Mamsen et al., 2017). *SOX9* regulates the transcription of anti‐mullerian hormone along with other male‐specific genes that promote androgen biosynthesis and the development of male sex organs. In the absence of *SRY*, female reproductive genes (*WNT4*, *RSPO‐1*, *FOXL2*) promote the development of ovaries and inhibit differentiation of the testis, resulting in estrogen and progesterone synthesis. The divergent mechanisms of primary sex hormones are enacted, in part, by the location of hormone synthesis and the function and regional distribution of target receptors. Biosynthesis of sex steroids primarily occurs in male and female reproductive organs, but they are also synthesized in the brain and other tissues de novo from cholesterol (Hu, Zhang, Shen, & Azhar, 2010). Many additional genetic and epigenetic factors have been identified in sex differentiating pathways, as detailed elsewhere (Mamsen et al., 2017; Rotgers, Jørgensen, & Yao, 2018).

Sex hormones are believed to have organizational (permanent) and activational (dynamic) effects that impact disease manifestation, timing, and neuropathological progression, with associated changes in brain structure and function (Herting & Sowell, 2017; Schulz & Sisk, 2016). Organizational effects of sex hormones are “permanent” effects related to sexual differentiation and development that occur during the perinatal period (Arnold & Breedlove, 1985) and puberty/adolescence (Schulz, Molenda‐Figueira, & Sisk, 2009; Schulz & Sisk, 2016). During these periods, sex hormones are believed to impart lasting effects on brain structure through complex gene‐biology interactions that influence dendritic spine growth, synaptogenesis, synaptic patterning, and pruning (Arnold & Breedlove, 1985; Herting & Sowell, 2017; McCarthy, 2008; Schulz et al., 2009; Schulz & Sisk, 2016). These organizational effects are implicated as underlying mechanisms of the “developmental origins” hypothesis—a widely accepted theory linking early life experiences to adult disease (McCarthy, Arnold, Ball, Blaustein, & de Vries, 2012). By contrast, activational effects of sex hormones refer to transient and dynamic effects of sex hormones that occur throughout life after neural circuits have been organized, typically during adulthood (Herting & Sowell, 2017; Schulz & Sisk, 2016).

The developmental trajectory of primary sex hormones is dynamic in both males and females. In males, testosterone levels rise during the perinatal period, reaching peak levels 1–3 months after birth. Afterward, testosterone levels decline sharply until they plateau around 7–12 postnatal months; levels sharply rise again at puberty and plateau again around age 17 (Forest, Sizonenko, Cathiard, & Bertrand, 1974; Johannsen et al., 2018; Mason, Schoelwer, & Rogol, 2020; Senefeld et al., 2020; Tomlinson, Macintyre, Dorrian, Ahmed, & Wallace, 2004). Estrogen levels in males remain low in early childhood and then modestly rise around age 8 until they peak between ages 16 and 18 years (Frederiksen et al., 2020). In females, estrogen levels rise shortly after birth until approximately age 1, when estrogen plateaus until puberty (~age 10). During puberty, estrogen levels increase until ages 15–16 (Bidlingmaier, Wagner‐Barnack, Butenandt, & Knorr, 1973; Frederiksen et al., 2020). Testosterone levels in females remain low throughout infancy and childhood, and modestly increase around age 6 until around age 14 (Søeborg et al., 2014). At the onset of puberty, females experience monthly fluctuations in estrogen and progesterone according to the menstrual cycle, as detailed in the sections below. Deviations from these normal sex‐specific hormone trajectories can permanently alter structural brain development and increase vulnerability to disease acutely and many years later (McCarthy, 2008; Pike, 2017).

Testosterone levels remain fairly stable throughout adulthood in both males and females (Handelsman, Sikaris, & Ly, 2016), with modest decline in males that begins around the fifth decade (Feldman et al., 2002; Harman et al., 2001). Testosterone levels are also higher in males than females throughout the lifespan (Rothman et al., 2011). Females experience more dynamic changes in primary sex hormones (estrogen, progesterone) during adulthood due to menstruation, pregnancy, and menopause (Del Río et al., 2018). Estrogen levels are higher in premenopausal females than males, but estrogen levels between males and females become similar after females experience menopause (Handelsman et al., 2016; Nugent et al., 2012; Rothman et al., 2011).

### 2.2 Testosterone

Testosterone is known for promoting muscle and bone growth, healthy libido, mood, and social behaviors such as aggression, competitiveness, and risk taking (Campbell et al., 2010; Casto & Edwards, 2016; Eisenegger, Haushofer, & Fehr, 2011; Tyagi, Scordo, Yoon, Liporace, & Greene, 2017; Walther, Wasielewska, & Leiter, 2019). The effects of testosterone on brain and behavior occur by binding to androgen receptors in the forebrain, midbrain, and brainstem, with the highest concentrations in the ventromedial hypothalamus, medial preoptic area, nucleus accumbens, basal nucleus of the *stria terminalis*, and septum (Davey & Grossmann, 2016). Neuroimaging investigations relating testosterone levels to brain structure are limited in healthy adults, but suggest a link between circulating testosterone and frontal‐temporal brain integrity. Specifically, a structural MRI study of healthy young adults (*N* = 34, 50% female; ages 21–47, *M*_age_ = 26.6 ± 5.0) reported a negative association between testosterone levels and gray matter volume in the left inferior frontal gyrus (IFG) after adjusting for sex and total gray matter volume (Witte, Savli, Holik, Kasper, & Lanzenberger, 2010). However, testosterone only explained 2.2% of total model variance compared to 32% and 47.2% explained by sex and gray matter volume, respectively. Sex‐stratified analyses did not show significant associations between testosterone and gray matter volumes, likely due to low statistical power from the small sample size and limited explanatory effect of testosterone on gray matter volume in the whole sample. More recently, a study of hippocampal volume in the Vietnam Era Twin Study of Aging cohort (*N* = 445 males, ages 51–60) showed that effects of salivary free testosterone (unbound to a receptor) on hippocampal volume differed based on a person's cortisol levels (Panizzon et al., 2018). Specifically, Panizzon et al. (2018) found that the effect of free testosterone on hippocampal volume was only significant when cortisol levels were >1 *SD* above or below the mean, such that hippocampal volumes were larger in individuals with high testosterone and high cortisol, but smaller in individuals with low testosterone and low cortisol. These associations were observed after covarying for age, ethnicity, twin pair, current alcohol use, depression, smoking status, and a history of cardiovascular disease, hypertension, and diabetes, and after correcting for lack of independence in the sample (i.e., twin clustering) (Panizzon et al., 2018).

### 2.3 Estrogen and progesterone

Estrogen and progesterone are important opposing sex hormones that fluctuate significantly across the female menstrual cycle. Estrogen impacts a wide range of positive biological functions beyond the reproductive system, including maintenance of bone mineral density, regulation of antioxidant defense systems and mitochondrial oxidation, maintenance of blood vessel structure and vascular tone, and enhanced neuron survival (Prabhushankar, Krueger, & Manrique, 2014; Ventura‐Clapier, Piquereau, Veksler, & Garnier, 2019). At the same time, estrogen is associated with increased production of the stress hormone, cortisol, upregulation of excitatory neurotransmitters (acetylcholine, dopamine) and downregulation of the inhibitory neurotransmitter, GABA (Barth, Villringer, & Sacher, 2015). These latter biological effects of estrogen increase vulnerability to adverse psychosomatic symptoms under certain conditions.

Estrogen exerts its effects by binding to G‐coupled receptors that activate second messenger systems and to intracellular estrogen receptor alpha (ER‐α) and beta (ER‐β) to modulate transcription (Fuentes & Silveyra, 2019). ER‐α and ER‐β, respectively, modulate the excitatory and inhibitory effects of estrogen, and both receptors are located throughout the limbic system, midbrain, and brainstem to regulate the stress response in the preoptic area, arcuate and lateral habenula, periaqueductal gray, locus coeruleus, and in nuclei of the amygdala, hypothalamus, *pons*, and *medulla oblongata* (Weiser, Foradori, & Handa, 2008). However, only ER‐α is found in the ventromedial nucleus of the hypothalamus and subfornical organ, whereas only ER‐β is found in the olfactory bulb, *zona incerta* of the subthalamus, ventral tegmental area, cerebellum, pineal gland, and hypothalamic nuclei of the supraoptic (SON), paraventricular, suprachiasmatic, and tuberal areas (Weiser et al., 2008). The distinct distribution of ER‐α and ER‐β in these brain regions allows for separate interactions with neurotransmitter systems to facilitate target functions.

Progesterone is a derivative of the hormone, pregnenolone, in both males and females. Although it is most commonly known for its role in female physiology, progesterone also facilitates sperm capacitation, fertilization and immunosuppression in both sexes (Maybin & Critchley, 2011; Oettel & Mukhopadhyay, 2004). In the brain, progesterone helps to maintain the structural integrity of myelin and regulates synaptogenesis, neuron survival and dendritic growth (Schumacher et al., 2012). Neural functions of progesterone occur primarily through membrane‐associated progesterone receptors in various brain regions including the hippocampus, amygdala, olfactory bulb, cortex, cerebellum, locus coeruleus, hypothalamus, thalamus, basal ganglia, and brainstem (Schumacher et al., 2012).

Estrogen and progesterone levels fluctuate throughout the female menstrual cycle, and these fluctuations have been associated with changes in mood, concentration, somatic sensations, and brain structure (Catenaccio, Mu, & Lipton, 2016). The follicular phase and luteal phase are the two primary phases of the menstrual cycle. Menstruation generally occurs between days 1 and 8 of the follicular phase, during which estrogen rises until reaching peak levels at the start of the peri‐ovulatory period of the follicular phase (~days 11–12). Ovulation, which typically occurs around Day 14 of the menstrual cycle, marks the transition from the follicular phase to the luteal phase, and a significant drop in estrogen levels. Simultaneously, progesterone levels begin to rise during the luteal phase (progesterone levels are low during follicular phase), peaking at the start of the pre‐menstrual period (~days 21–22) and declining rapidly thereafter (Catenaccio et al., 2016; Maybin & Critchley, 2011). A recent review of 25 neuroimaging studies (*N* = 1,321) (Catenaccio et al., 2016) described the brain signatures that corresponded to these phases. Four studies reported larger volumes during the follicular phase than the luteal phase, with the most consistent effects in the hippocampus, parahippocampal and middle frontal gyri. Five studies reported larger volumes during the luteal phase than the follicular phase using a mix of voxel‐based morphometry (VBM) and region of interest (ROI) methods, but none reported effects in consistent regions. Three studies reported no volume differences between follicular and luteal phases. A few studies compared neuroimaging metrics (VBM, volumetry, ROI‐based) between the menstrual and peri‐ovulatory periods, but none reported consistent effects in any regions (Catenaccio et al., 2016). It is worth noting, however, that the sample sizes of each of the 25 reviewed studies was small (largest *N* = 128, mean *N* ~ 32) and effect sizes were not reported in any systematic way.

### 2.4 Exogenous sources of estrogen and progesterone

Studies of hormone contraceptive use provide naturalistic designs to understand relationships between hormonal fluctuations and brain health. These studies are important as approximately 25% of premenopausal females use oral hormone contraceptives or long‐acting reversible contraceptives (Daniels & Abma, 2020). There is increasing recognition that hormone contraceptives can influence short and long‐term changes in brain structure. Lisofsky, Riediger, Gallinat, Lindenberger, and Kühn (2016) showed that even brief use of oral contraceptives (the pill) can result in structural brain changes in regular cycling females (ages 16–35) using VBM. Specifically, female contraceptive users (*n* = 28) showed volume reductions in the left amygdala and anterior parahippocampal gyrus (PHG) compared to age‐matched controls (*n* = 28) after 3 months of daily contraceptive use and after adjusting for age, intracranial volume (ICV), and total estrogen and progesterone levels. These regions are key hubs for emotion processing and regulation and may explain affective changes associated with contraceptive use (Montoya & Bos, 2017). However, individuals on oral contraceptives were not on the same pill type regimen, and it is unclear how different pill types (estrogen + progestin vs. progestin‐only, etc.) may have influenced these results.

Our group recently expanded on this work in the UK Biobank cohort by examining the impact of oral contraceptive use on whole‐brain white matter in premenopausal and postmenopausal females using DTI (Nabulsi & Lawrence, 2020)—a noninvasive imaging technique that measures patterns of water diffusion throughout the brain (Basser & Pierpaoli, 1996). The most common diffusion MRI metric is fractional anisotropy (FA), which measures the degree of diffusion restriction within an image voxel. In a trajectory analysis using fractional polynomial modeling, Nabulsi and Lawrence (2020) revealed higher whole‐brain FA and tensor distribution function (TDF, a more rigorous multitensor diffusion model that accounts for intravoxel fiber crossing) FA in contraceptive users compared to never‐users (*n* = 7,136 users, *n* = 1,177 nonusers; ages 45–80 years) after adjusting for age, years of education, socioeconomic status (SES), waist‐to‐hip ratio, and genetic ancestry. Conversely, longer duration and younger age at first contraceptive use were associated with lower FA and TDF‐FA compared to never users. As higher FA is typically a good indicator of “healthy” white matter (Basser & Pierpaoli, 1996; Bennett, Madden, Vaidya, Howard, & Howard Jr, 2010), these results suggest that brief oral contraceptive use may serve a protective role for white matter microstructure, but chronic use—particularly at older ages—may be associated with a faster rate of white matter decline in older adulthood.

In addition to contraceptive use, hormone replacement therapy (HRT) may have neuroprotective effects when administered during perimenopause (Eberling, Wu, Haan, & Mungas, 2003; Shao et al., 2012). Specifically, earlier studies suggested that HRT implemented early in menopause (Eberling et al., 2003; Shao et al., 2012) or over long periods may attenuate risk of AD diagnosis (Imtiaz et al., 2017) and death (Mikkola et al., 2017) in females. Indeed, neuroimaging work by Pintzka and Håberg (2015) showed that females who initiated HRT prior to menopause and remained on HRT for at least 3 years (*n* = 80) had greater whole hippocampal volume compared to HRT‐naive females (*n* = 80) who were matched for ICV and age (ages 51–66). A recent voxelwise study by Boyle et al. (2020) also revealed larger gray and white matter volume in various regions in females with a history of HRT (*N* = 562, ages 71–94) after adjusting for data acquisition site, age, ethnicity, years of education, clinical diagnosis, history of heart disease, Type 2 diabetes, and hypertension, presence of white matter lesions, BMI, physical activity, and past‐year estrogen use, but the duration of HRT was not associated with imaging variables.

Other work suggests that HRT does not prevent cognitive decline (Henderson et al., 2016) and that long‐term HRT may slightly increase risk for dementia (Savolainen‐Peltonen et al., 2019; Wu et al., 2020). Indeed, a randomized, double‐blinded placebo controlled trial of HRT (specifically, conjugated equine estrogens) in 95 recently postmenopausal women (ages 42–56) showed decreases in whole brain volume, increased ventricle expansion, and increased white matter hyperintensity volume over 48 months of HRT compared to the placebo group. However, cognitive performance did not differ between groups (Kantarci, Tosakulwong, et al., 2016). Finally, Nabulsi & Lawrence, 2020] earlier mentioned work on contraceptive use also examined the impact of HRT on brain white matter in the UK Biobank using both DTI and neurite orientation dispersion density imaging (NODDI). Results revealed lower whole‐brain white matter fiber coherence (orientation dispersion index) in females on HRT (*N* = 3,106) compared to never‐users (*N* = 5,195), but less pronounced white matter changes with age (Nabulsi & Lawrence, 2020). Further, females on an estrogen‐only regimen showed greater white matter disruption with age (lower fiber dispersion, and increased free water).

The inconsistent neuroimaging markers of HRT emphasize that there is much to learn regarding the therapeutic potential of exogenous estrogen use. A major limitation of previous work is the variability in HRT chemical composition used across individuals, even within the same study. Prior work on HRT has compared studies using various hormonal combinations (e.g., estrogen, androgen, progesterone treatment alone or together), chemical formulations, doses, and delivery routes that exert different effects on the body (Comasco, Frokjaer, & Sundström‐Poromaa, 2014; Maki & Dumas, 2009; Moraga‐Amaro, van Waarde, Doorduin, & de Vries, 2018; Yare & Woodward, 2020). In fact, estrogen and progesterone have various molecular and binding mechanisms that modulate neurotransmitter activity, sometimes in opposing directions (Del Río et al., 2018). This variability and lack of consensus of HRT effects on brain structure is a major gap in the literature that requires immediate attention given the growing population of older adults and the high percentage of females taking HRT worldwide (Boyle et al., 2020).

## 3 NORMATIVE SEX DIFFERENCES IN HUMAN BRAIN STRUCTURE

### 3.1 Brain size metrics and gray matter

Neuroimaging has been particularly informative for understanding how sex differences in brain size can be explained by differences in underlying gray and white matter microstructure. At the outermost level, ICV remains an important index of maximal brain size across the lifespan, as the intracranial vault is set at approximately age 7 and is not susceptible to developmental or degenerative processes (O'Brien et al., 2006). Sex differences in ICV have been established in both large and small‐scale studies. On average, males have significantly larger ICV than females (Ruigrok et al., 2014), and these differences have been shown to account for some, but not all, regional sex differences in brain volumes (Jahanshad & Thompson, 2017). The literature also shows that total brain volume (TBV)—measured on T1‐weighted brain scans—is approximately 9–12% larger in males than females in childhood, adolescence, and adulthood in both large and small scale studies (for review, see Kaczkurkin, Raznahan, & Satterthwaite, 2019; Lenroot & Giedd, 2010). This aligns with very early evidence that brain weight is approximately 10% larger in males than females at autopsy (Pakkenberg & Voigt, 1964; Voigt & Pakkenberg, 1983).

Similarly, sex differences in gray matter measures are well‐established across the lifespan. In young adults (ages 20–34 years), VBM has revealed larger total normalized gray matter volume (total gray matter volume/total ICV) but also a faster rate of age‐related decline in normalized gray matter volume in females (*n* = 71) than males (*n* = 71) (Farokhian, Yang, Beheshti, Matsuda, & Wu, 2017). Yang, Bozek, Han, and Gao (2020) revealed sex differences in several different cortical gray matter features, including sulcal depth and cortical thickness, in young adults (ages 19–37) from the Chinese and U.S. Human Connectome Project (HCP) cohorts using sample‐specific surface templates based on FreeSurfer segmentations from 35 Desikan–Killiany (DK) and 75 Destrieux atlas structures in each hemisphere. In the Chinese HCP, males (*n* = 100) exhibited greater cortical thickness than females (*n* = 100) in the frontal, temporal, and parietal lobes after correcting for age and ICV, whereas in the U.S. HCP, males (*n* = 100) exhibited lower thickness than females in the caudal anterior cingulate cortex (ACC), but greater thickness in the insula, lateral orbitofrontal cortex (OFC), and the isthmus of the cingulate than females (*n* = 100).

The Enhancing Neuroimaging Genetics through Meta‐Analysis (ENIGMA) consortium has begun to chart neuroimaging sex differences across the lifespan to provide a benchmark for evaluating individual brain health and improve disease detection and monitoring (Dima, Papachristou, Modabbernia, & Doucet, 2020; Frangou, Modabbernia, Doucet, Moser, et al., 2020; Wierenga et al., 2020). Recent mega‐analyses of subcortical volumes and cortical thickness by the ENIGMA Lifespan Working Group has shown that while unadjusted overall cortical volume and thickness are larger in males than females, sex differences do not persist after covarying for ICV (Dima et al., 2020; Frangou, Modabbernia, & Doucet, 2020). Trajectory analyses, however, do show significant sex differences in the rate of change in cortical thickness across the lifespan. Specifically, Frangou, Modabbernia, and Doucet (2020) showed that males (*n* = 8,212), on average, had faster whole‐brain cortical thinning than females (*n* = 8,863) during midlife (ages 30–59), but cortical thinning rates were comparable between males and females in early life (ages 3–29) and in older adulthood (ages 60–90). Males also had faster regional cortical thinning than females in motor, somatosensory, and visual association cortices during early life (ages 3–29), and in frontal‐temporal cortical areas during midlife (Frangou, Modabbernia, & Doucet, 2020). Of note, sex was the only covariate used for trajectory modeling, as the imaging data were harmonized using the ComBat package in R, which adjusts for site and scanner‐related variance.

In more recent work from the ENIGMA‐Lifespan group, Wierenga et al. () examined cortical and subcortical brain metrics in 16,683 individuals between ages 1 and 90 years (47% females) using the ENIGMA FreeSurfer (Fischl, 2012) cortical and subcortical pipelines http://enigma.ini.usc.edu/protocols/imaging-protocols/ (ENIGMA, 2017). Results revealed sex differences both in brain structure metrics and in between‐subject variability in brain metrics after adjusting for cohort, magnetic field strength, FreeSurfer version, and age. Specifically, males had larger volumes than females in all subcortical ROIs (Cohen's *d* range = 0.41 [left accumbens] to 0.92 [right thalamus], and these differences persisted with slightly smaller effect sizes after covarying for TBV (*d* range = 0.21 [left accumbens] to 0.58 [right thalamus]). Males also had greater regional surface area than females across the entire cortex with and without adjusting for total surface area (without: *d* range = 0.42 [left ACC] to 0.97 [left superior temporal gyrus, STG]; with: *d* = 0.21 [left ACC] to 0.59 [left STG]). Females had greater thickness than males in 38 of the 68 DK atlas‐defined cortical regions, but effect sizes were comparatively small (largest effect, *d* = 0.12 in the right caudal ACC), and effects in several regions changed direction (males > females) or became nonsignificant when total thickness was included as a covariate. However, males showed significantly greater between‐subject variability than females for all subcortical volumes and cortical surface area metrics, and for 60% of cortical thickness metrics, and these differences persisted throughout the lifespan (Wierenga et al., 2020).

Population‐based studies of middle‐aged and older adults (ages 45–80 years) from the UK Biobank cohort generally align with the findings from the ENIGMA‐Lifespan group. Specifically, Ritchie et al. (2018) reported greater cortical surface area in males (*n* = 2,466) than females (*n* = 2,750) in most regions after adjusting for TBV, age, and ethnicity. Conversely, females had greater thickness than males across most of the cortex, except the medial OFC and rostral ACC, which was thicker in males. Females also had larger volumes in the nucleus accumbens compared to males after adjusting for TBV, whereas males had larger volumes in the putamen, amygdala, and pallidum after adjusting for the same covariates (Ritchie et al., 2018). Other work in the UK Biobank revealed significant age‐by‐sex interaction effects in subcortical volume trajectories, such that males (*n* = 12,665) exhibited faster decline than females (*n* = 13,775) in all volumes across the full age range (44–81 years) after adjusting for ICV, education, and BMI (Ching et al., 2020). Interestingly, sex differences in volume loss were significantly attenuated after age 60, suggesting that the greatest sex effects on subcortical volumetry may occur during middle age.

#### 3.1.1 Hippocampal volume

The hippocampus is a complex subcortical brain structure that serves as an essential integratory hub for memory (formation, storage, retrieval), spatial navigation, and emotional processing. As sex differences are reported in these functions, numerous imaging studies have investigated hippocampal volume in clinical and non‐clinical populations (Yagi & Galea, 2019). Consistent with population‐based studies from ENIGMA and the UK Biobank, a meta‐analysis of hippocampal volumes in healthy participants from 76 studies (*N* = 4,418, ages 0–79 years, 45.3% females) showed that raw hippocampal volumes were larger in males than females by ~6–7%, but statistical adjustments for ICV or TBV nullify these sex differences (Tan, Ma, Vira, Marwha, & Eliot, 2016). Similarly, nomograms (percentile charts) of whole hippocampal volume (computed with FSL‐FIRST) in 19,793 individuals from the UK Biobank (ages 45–80 years, 52.9% females) showed similar hippocampal volume measurements by sex after adjusting for age, scan date, and an automated metric of head size derived from the nomogram pipeline (https://lnobis.github.io/HippoFit_Tool/index.html) (Nobis et al., 2019). However, trajectory analyses revealed significant sex differences in the rate and temporal change of hippocampal volume loss with age, with accelerated loss in males around age 50 and accelerated loss in females between ages 60 and 65. The rate of hippocampal volume loss relative to total gray matter volume also differed in males and females, with peak inflection points around ages 63 and 67, respectively (Nobis et al., 2019).

Finally, while sex differences in total hippocampal volume do not persist after correcting for head size, prior work suggests that subregions of the hippocampus are sexually dimorphic. In a recent lifespan study of hippocampal subfield volumes manually segmented from 4.7 T scans (*N* = 129, ages 18–85, *M*_age_ = 47.6 ± 18.9), Malykhin, Huang, Hrybouski, and Olsen (2017) found larger subfield volumes in the hippocampal head (dentate gyrus [DG]), body (CA1‐3, subiculum, DG), tail (all subfields), and DG in females (*n* = 70) than males (*n* = 59) after normalizing hippocampal volumes by ICV (raw hippocampal volume/ICV of same subject × sample averaged ICV) and removing the effects of age. Sex‐by‐age interactions were significant in the subiculum of the hippocampal tail, with a marginally significant nonlinear effect of age on subiculum volumes in females, but no significant effect in males (Malykhin et al., 2017).

Interestingly, a larger study of young adults (ages 21–36) from the Queensland Twin Imaging study (QTIM, 4 T scanner) and HCP (3 T scanner) revealed larger subfield volumes (segmented with the FreeSurfer‐v.6.0 hippocampal subfield pipeline) in males (*n* = 692) than females (*n* = 995) in the fimbria, parasubiculum, fissure, and presubiculum after statistically adjusting for whole hippocampal volume (van Eijk et al., 2020). Importantly, sex differences persisted across four different statistical methods to control group differences in whole hippocampal volume: (a) *allometric scaling*—regresses out the effect of whole hippocampal volume (or comparable metric) after identifying the scaled relationship between whole hippocampal volume and each subfield via log–log regression, (b) *covariate—*models the effect of whole hippocampal volume as a covariate predictor in regression or analysis of covariance (ANCOVA), (c) *residuals—*regresses out the effect of whole hippocampal volume on the subfield ROI and uses the residuals as the dependent variable, and (d) *matched—*where groups are matched by whole hippocampal volume. When subfields were adjusted for brain segmentation volume (a FreeSurfer defined metric of total brain size that includes gray matter, white matter, and CSF) rather than whole hippocampal volume, males again had larger volumes than females in the hippocampal fissure, presubiculum, and parasubiculum using all four correction methods, albeit at smaller effect sizes for subicular subregions. Males also had larger volumes than females in the fimbria and subiculum using covariate, residual, and matching methods, but not when using allometric scaling. Sex differences were not detected in the CA2/3, CA4, hippocampal–amygdala transition area (HATA), or DG using any normalization technique or covarying for whole hippocampal volume or brain size (van Eijk et al., 2020). While these findings differ considerably from those reported by Malykhin et al. (2017), they may be explained by key methodological distinctions including (but not limited to) considerable differences in sample size and composition (lifespan approach [mean age ~mid 1940s] vs. young adults [mean age ~mid 1920s]), use of manual vs. automated hippocampal subfield parcellation, and use of different normalization techniques and covariates.

#### 3.1.2 Laterality

Brain laterality is an important metric of brain organization and contributes to many cognitive processes. Thus, understanding sex differences in brain asymmetries can inform mechanisms of sex differences in cognitive function (visuospatial processing, spatial navigation) and risk factors for various brain diseases (e.g., AD, schizophrenia [SCZ]) (Kong et al., 2018). In the general population, our ENIGMA‐Laterality Working Group recently revealed greater right‐ward asymmetry in the putamen and greater left‐ward asymmetry in the globus pallidus in males compared to females in a volumetric meta‐analysis of subcortical brain structures in 15,847 participants (median age across studies = 33.9 years, 53% female, (Guadalupe et al., 2017). A subsequent meta‐analysis of cortical data by the same group revealed more leftward asymmetry in cortical thickness of the PHG and entorhinal cortex (ERC), and more rightward asymmetry of global surface area in males versus females in 17,141 individuals ages 3–90 years (Kong et al., 2018). Regional analyses showed the most pronounced rightward asymmetry in surface area of frontal, temporal, parietal, and anterior cingulate cortices. Results were replicated in two independent samples, including the young adult HCP cohort (Kong et al., 2018).

### 3.2 White matter

Normative sex differences in white matter microstructure have been shown using DTI metrics. Our ENIGMA‐DTI Working Group (*N* = 481, 60% female, ages 22–36 years) showed that FA is a heritable metric, with age, sex, age‐by‐sex, age^2^, and age^2^‐by‐sex explaining 10% of the total variance in whole‐brain average FA, and genetic factors explaining 78% of the remaining variance in FA (Kochunov et al., 2015). Sex was the only significant covariate predictor of whole‐brain FA, with ~2% higher whole‐brain average FA in females than males. Regionally, sex predicted higher FA in females than males for most white matter tracts, with the strongest sex effects in the internal capsule and fornix (19.1 and 14% variance explained, respectively), and the weakest effects in the superior fronto‐occipital fasciculus and inferior fronto‐occipital fasciculus (IFOF; 1.3 and 1.6%, respectively). Whole‐brain FA was highly heritable in both females and males when examined separately, with genetic factors explaining 85.7% and 91.9% of total FA variance (respectively). A weak proportion of FA variance was attributed to linear and nonlinear age effects in both males (1.5%) and females (0.15%), which is consistent with the age range of the cohort (Kochunov et al., 2015).

Later work in a slightly younger cohort (*N* = 667, ages 18–30, 62% females) of 415 families revealed a slightly different relationship between age and sex on FA measurements along the length of specific fiber tracts using high angular resolution diffusion imaging (Dennis et al., 2017). Results revealed significant age‐by‐sex interactions in right frontal callosal fibers and the right IFOF, with greater positive associations between FA and age in females than males after adjusting for family relatedness (random effect), age, and age^2^ (fixed effects). Although effect sizes were not reported, marginally higher FA in the IFOF of females than males is consistent with DTI findings in middle age and older adults in the UK Biobank cohort (ages 44–77 years) (Ritchie et al., 2018). Here, females (*n* = 2,750) had higher FA than males (*n* = 2,466) in the left inferior longitudinal fasciculus (ILF; *d* = 0.14) and posterior thalamic radiation (PTR; *d* = 0.12) after adjusting for TBV, age, age‐by‐sex interactions, and ethnicity. By contrast, males had significantly higher FA than females in the right arcuate fasciculus (ARC; *d* = 0.26), bilateral corticospinal tract (CST) (right *d* = 0.22, left *d* = 0.15), and bilateral superior thalamic radiation (STR) (right *d* = 0.16, left *d* = 0.15). Interestingly, analyses that did not adjust for group differences in TBV revealed stronger sex differences in FA (male > female) in the ARC, CST, and STR (*d* range = 0.26–0.56) and weaker sex differences (female > male) in the ILF and PTR (*d*'s = 0.10) and sex differences in the IFOF became nonsignificant compared to analyses that covaried for TBV. Interestingly, advanced diffusion metrics from NODDI revealed greater tract complexity (higher orientation dispersion) in females than males in all white matter tracts after adjusting for all covariates (average *d* = 0.30), but the functional significance of this finding is currently unknown (Ritchie et al., 2018).

## 4 SEX DIFFERENCES IN ADULT PSYCHIATRIC ILLNESS

**FIGURE 1:**
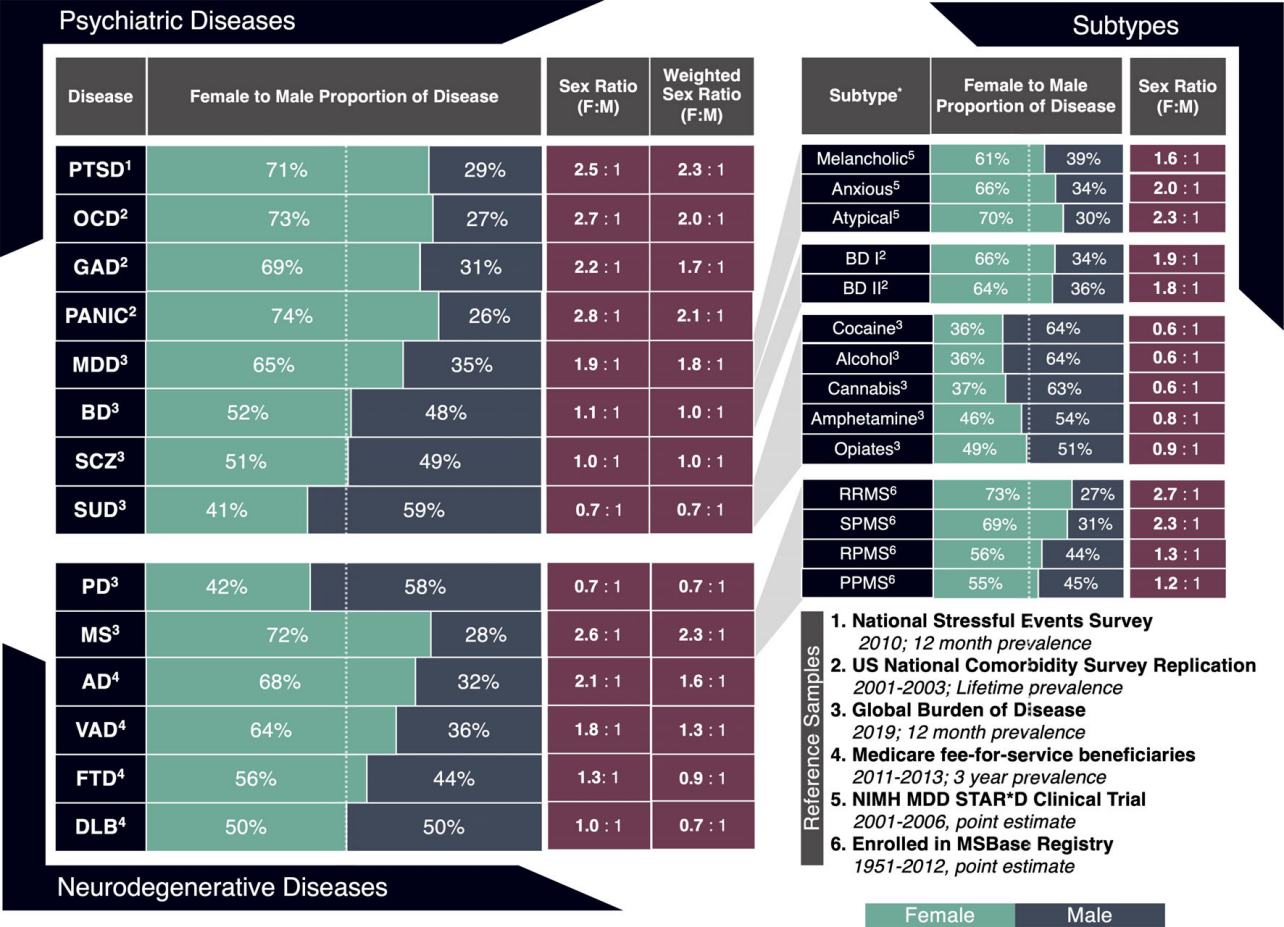
Sex composition of common neurological and psychiatric disorders in males and females in the United States. Male/female proportion of disease, where percentages reflect the male (*dark blue*) and female (*green*) proportion of the reference sample with each disease. The dashed line indicates equal proportion of males and females with disease (i.e., 50/50). Sex ratio reflects the absolute number of females with disease for every one male with disease within each sample, which was calculated by dividing the number of females with each disease by the number of males with each disease for each reference sample.The weighted sex ratio reflects the adjusted sex ratio after accounting for male and female differences in sample size. It was calculated by dividing the female disease prevalence (i.e., *N* females with disease/*N* total females) by the male disease prevalence (i.e., *N* males with disease/*N* total males) from each reference sample. Proportions and ratios *do not* reflect base rates of disease. *Reference samples*: Posttraumatic stress disorder (PTSD) calculations are based on 12‐month estimates from the 2010 National Stressful Events Survey using Diagnostic Statistical Manual (DSM‐V) criteria (*N* = 2,953, ages >18 years) (*N* = 36,309 adults) (Kilpatrick et al., 2013). Obsessive compulsive disorder (OCD), generalized anxiety disorder (GAD), and panic disorder (PANIC) calculations were obtained from the National Comorbidity Survey Replication (NCS‐R) (*N* = 9,282, ages >13 years) (Kessler, Petukhova, Sampson, Zaslavsky, & Wittchen, 2012) Major depressive disorder (MDD), bipolar disorder (BD), schizophrenia (SCZ), substance use disorders (SUDs), Parkinson's disease (PD), and multiple sclerosis (MS) calculations were taken from the 2019 Global Burden of Diseases (GBD) study based on 12‐month point estimates for the United States. Dementia subtype (Alzheimer's disease [AD], vascular dementia [VAD], frontotemporal lobe dementia [FTD], and dementia with Lewy bodies [DLB]) calculations were based on medicare fee‐for‐service beneficiaries (*N* = 21,624,228; age > 68 years), of whom 3,110,654 had dementia (Goodman et al., 2017). MDD subtypes were derived from patients enrolled in the NIMH Sequenced Treatment Alternatives to Relieve Depression (STAR*D) trial (ages 18–71 years), with sex‐specific subtype analysis derived from 952 males and 1,589 females with MDD (Marcus et al., 2008). BD I and II were not available by sex in the GBD, so we present the annual first incidence rate of BD I and II in males and females from a U.S. sample (*N* = 34,653) (Grant et al., 2009). MS subtypes were derived from the MSBase registry sample (RRMS *N* = 6,452; SPMS *N* = 594; RPMS *N* = 303, PPMS *N* = 881) collected between the years 1951 and 2012 across 25 countries and 55 MS centers (Kalincik et al., 2013). Collectively, these data represent the most current large‐scale information on sex‐specific disease composition. *Weighted sex ratios were not calculated for disease subtypes, as the majority of the samples were not derived from the general population

Many psychiatric conditions show sex differences in prevalence and incidence rates (Figure 1), age of onset, clinical presentation, or treatment efficacy, yet information on sex differences in brain imaging signatures is surprisingly limited. Below we discuss the extant literature on sex differences in neuroimaging, as well as clinical features and expression patterns for adult onset psychiatric disorders. As noted in Section 1, we focus our review on common psychiatric conditions that have been linked to increased risk for neurodegenerative conditions and dementia, as these may yield early neuroimaging cues that can be used for risk profiling, disease monitoring, and ideally the development of new treatments and interventions.

### 4.1 Posttraumatic stress disorder

Posttraumatic stress disorder (PTSD) is a complex condition that develops after exposure to a serious and often life‐threatening event (Nisar et al., 2020). Core symptoms include avoidance, hyperarousal, reexperiencing, and negative alterations in mood and cognition. A combination of these must persist for at least 1 month to meet criteria for PTSD (National Institute of Mental Health, 2019). The lifetime prevalence of PTSD is significantly higher for females (2.6%) than males (1.0%) cross‐nationally (*N* = 71,083) (Koenen et al., 2017), with around twice the prevalence for females (lifetime = 6.1%, 12‐month = 6.1%) than males (lifetime = 4.1%, 12‐month = 3.2%) in the United States (Goldstein et al., 2016), despite experiencing similar numbers of traumatic events (Lehavot, Katon, Chen, Fortney, & Simpson, 2018). In contrast to other psychiatric disorders, there are inherent sex biases related to trauma exposure type and the pathophysiology of PTSD. For example, females tend to be overrepresented in civilian cases of PTSD as a result of intimate partner violence and/or rape (Laskey, Bates, & Taylor, 2019), whereas males are significantly overrepresented in studies of combat‐exposed PTSD (Lehavot et al., 2018). Still, a U.S.‐based epidemiology study of PTSD (*N* = 36,101, 56.5% females) revealed significantly higher prevalence in 12‐month and lifetime PTSD in female versus male veterans (12‐month 11.7 vs. 6.7%; lifetime: 13.4 vs. 7.7%) and civilians (12‐month: 6 vs. 2.6%; lifetime: 8 vs. 3.4%) (Lehavot et al., 2018).

Sex differences in clinical patterns have also been reported, with females demonstrating greater acute PTSD symptoms (*d* = 0.24), peritraumatic dissociation (*d* = 0.21), and perceived life threat (*d* = 0.32) than males in a hospital setting, as well as greater PTSD symptoms 6 weeks (*d* = 0.52) and 6 months (*d* = 0.66) after trauma exposure (Irish et al., 2011). Females also show greater acquisition of conditioned fear and greater fear response to a conditioned stimulus compared to males (Inslicht et al., 2013). The severity of fear response and corresponding symptoms also varies by trauma exposure type in females, but not in males (Lancaster, Melka, Rodriguez, & Bryant, 2014).

The most common structural neuroimaging signatures of PTSD include volumetric abnormalities in the hippocampus, amygdala, and prefrontal cortex (PFC) (Akiki et al., 2017; Bae et al., 2019; Dennis et al., 2019), regions that collectively describe the “fear circuit.” Indeed, a meta‐analysis of subcortical brain volumes by our ENIGMA‐Psychiatric Genetics Consortium (PGC) PTSD Working Group (a partnership with the PGC) recently revealed significantly smaller volumes in the hippocampus, and a trend for smaller volumes in the amygdala, in 794 PTSD individuals compared to 1,074 controls (mostly trauma‐exposed) after adjusting for age and ICV (Logue et al., 2018). A sex‐by‐diagnosis interaction effect on hippocampal volume was not significant, but sex‐specific analyses revealed smaller hippocampal volumes in female PTSD patients (*n* = 308) versus female controls (*n* = 428; *p* < .001), with stronger effect sizes in females (*d* = −0.31) than the full sample (*d* = −0.17). Hippocampal volumes did not differ significantly between cases (*n* = 472) and controls (*n* = 629) in males (*p* = .23), but Cohen's *d* confidence intervals overlapped in males and females, suggesting limited power to detect a PTSD effect in males, despite being a larger subsample than females (Logue et al., 2018).

The ENIGMA‐PGC PTSD Working Group recently published a mega‐analysis of cortical volumes, revealing smaller volumes in 1,379 PTSD patients compared to 2,192 controls (aged 6–85 years) across most of the cortex (particularly in frontal‐temporal areas) after adjusting for age, sex, ICV, and a random intercept to model cohort and scanner differences across sites (Wang, Xie, et al., 2020). Although sex had a significant main effect on most cortical regions, sex‐by‐diagnosis interactions were not significant in any region. Similarly, a meta‐analysis by our group of PTSD effects on white matter tracts revealed significantly lower FA in the tapetum (a tract connecting the left and right hippocampi), but did not detect significant sex effects in any tract (Dennis et al., 2019).

### 4.2 Anxiety disorders

In the current version of the Diagnostic Statistical Manual (DSM‐5), nine distinct conditions are recognized as anxiety disorders: (a) generalized anxiety disorder (GAD), (b) social anxiety disorder, (c) separation anxiety disorder, (d) panic disorder (PaD), (e) specific phobia, (f) agoraphobia, (g) selective mutism, (h) anxiety disorders due to substance use, and (i) anxiety disorders due to another medical condition. Anxiety disorders are the most common mental health conditions worldwide (Ritchie & Roser, 2018), affecting over 3 million individuals in 2019 (Institute for Health Metrics and Evaluation (IHME), 2019). The nosology of anxiety disorders has evolved over the past decade, particularly with the transition from version 4 to version 5 of the DSM in 2013. Importantly, previously categorized anxiety disorders such as PTSD and obsessive compulsive disorders (OCD) are now recognized as independent diagnostic conditions in the DSM‐5. This has complicated the global picture of sex differences in psychiatric disease prevalences, as other diagnostic systems (e.g., International Classification of Diseases‐10) do not distinguish OCD and PTSD from other anxiety disorders. Nevertheless, most anxiety disorders are more common in females than males (Bekker & van Mens‐Verhulst, 2007; Jalnapurkar, Allen, & Pigott, 2018; McLean, Asnaani, Litz, & Hofmann, 2011).

Here, we focus on GAD and PaD, two of the most common anxiety disorders in adults. Social anxiety disorders and specific phobias affect a greater proportion of the population than GAD and PaD (Kessler, Chiu, Demler, Merikangas, & Walters, 2005; McLean et al., 2011) but are primarily linked to childhood/developmental origins (de Lijster et al., 2017). As such, we focus on anxiety disorders with adult onset to maintain a clear and concise narrative.

### 4.3 Generalized anxiety disorder

GAD is characterized by excessive, uncontrollable worry that interferes with activities of daily living (ADLs). Symptoms of GAD often include restlessness, insomnia, fatigue, irritability, and difficulty concentrating (Terlizzi & Villarroel, 2020). In a survey of 30,000 adults from the United States, nearly 15% reported some form of GAD symptoms in the 2 weeks preceding the survey; nearly 10% reported mild GAD symptoms and 3% reported severe symptoms. Approximately 6% of the total sample reported symptoms severe enough to meet clinical criteria for probable GAD. When stratified by sex, up to 19% of females reported GAD symptoms of any severity, compared to only 12% of males (Terlizzi & Villarroel, 2020). Globally, the lifetime prevalence of GAD is 3.7% (1.8% past year), with a disproportionate impact on females (OR = 1.8) and in higher‐income countries such as Australia (8%), New Zealand (7.9%), and the United States (7.8%) (Ruscio et al., 2017). Sex differences in GAD prevalence in the United States are similar to global estimates, with a nearly twofold greater prevalence in females than males (12‐month, OR = 1.74; lifetime, OR = 1.83) (McLean et al., 2011). Symptoms of GAD also differ by sex, with females reporting greater somatic issues (muscle tension, fatigue), neuroticism, and negative affect than males. Females also tend to have earlier symptom onset and lower remission rates than males (Yonkers, Bruce, Dyck, & Keller, 2003). GAD also has a high comorbidity profile that differs significantly by sex. Specifically, females with GAD are more likely than males to have comorbid depression and other anxiety disorders, whereas males with GAD are more likely than females to have comorbid alcohol, nicotine and other drug use disorders as well as antisocial personality disorder (Jalnapurkar et al., 2018).

Neuroimaging studies of GAD have shown abnormalities in both gray matter and white matter microstructure using structural and diffusion MRI. A brief review by Maron and Nutt (2017) noted that larger volumes in subcortical brain structures of GAD patients versus controls, particularly in the amygdala and dorsomedial PFC, were among the most common structural MRI signatures of GAD. However, a more recent systematic review of 26 structural imaging studies (Madonna, Delvecchio, Soares, & Brambilla, 2019) reported inconsistent effects of GAD in gray and white matter of these regions, and in the dorsolateral and ventrolateral PFC, ACC, and posterior parietal regions (larger and smaller volumes reported in GAD patients vs. controls). These paradoxical findings are likely due to heterogeneous research designs, imaging protocols, sample composition (inclusion vs. exclusion of comorbid psychiatric disorders), and remarkably small sample sizes across studies (average number of GAD participants per study = 10–25). Interestingly, however, DTI studies consistently reported lower FA in the uncinate fasciculus of GAD patients versus controls (Madonna et al., 2019).

We were surprised to find that almost no studies have examined the role of sex on structural brain signatures in GAD using neuroimaging, and to our knowledge this work has only been conducted in adolescents (Liao et al., 2014). The lack of dedicated neuroimaging studies in adult samples of GAD is a major gap in the literature, particularly given the significant role of sex in disease prevalence and clinical symptoms. In their 2017 review, Maron and Nutt (2017) noted that larger volumes in the amygdala and dorsomedial PFC in GAD were commonly observed in all‐female or predominantly‐female samples, but the role of sex was not explicitly tested. Thus, testing and comparing the directionality of GAD effects in the amygdala and PFC between males and females may be an ideal starting point for future neuroimaging investigations of GAD.

### 4.4 Panic disorder

PaD is a debilitating anxiety disorder characterized by abrupt and unpredictable episodes of heightened physiological arousal (i.e., panic attacks) that cause significant mental distress and physical discomfort. PaD affects 2.7% of the global population at some point during life, with median onset around age 32 (de Jonge et al., 2016). Similar to GAD, PaD is disproportionately represented in high‐income countries, with the highest 12‐month and lifetime prevalence in the United States. Cross‐nationally, both lifetime and 30‐day prevalence of PaD are significantly more prevalent in females (lifetime, OR = 1.8, 30‐day, OR = 2.0) than males (lifetime, OR = 1.0, 30‐day, OR = 1.0). Approximately 70–80% of individuals with PaD meet criteria for at least one comorbid mental health condition, most commonly another anxiety or mood disorder (de Jonge et al., 2016).

Clinical symptom expression of PaD also differs between males and females. Results of the United States National Comorbidity Study (*N* = 8,089, ages 15–54) showed that females with PaD (*n* = 194) experienced greater frequency of panic‐related symptoms than males with PaD (*n* = 80), including shortness of breath, nausea, and feeling smothered. Conversely, males with PaD reported greater frequency of stomach pain and sweating compared to females with PaD (Sheikh, Leskin, & Klein, 2002). A more recent study of PaD patients from South Korea indicated that females with PaD (*n* = 291) reported a greater number of stressful life events compared to males with PaD (*n* = 254), including physical injury, pregnancy‐related issues, and separation from parent, friend, or romantic partner. A series of self‐report questionnaires also showed that males with PaD were more likely to endorse a confrontational coping style, and were more likely to seek social support compared to females with PaD. Here, males and females reported comparable levels of depression and panic‐related symptoms, but females reported significantly greater symptoms of agoraphobia than males. Finally, females reported significantly lower physical functioning than males (Kim, Song, & Lee, 2017).

Neuroimaging markers of PaD include structural abnormalities in the amygdala, nucleus accumbens, thalamus, striatum, hippocampus, cerebellum, insula, ACC, midcingulate, IFG, STG, and somatosensory cortex using VBM (Del Casale et al., 2013; Wang, Cheng, et al., 2020). The direction of effects in these regions has been somewhat inconsistent across studies, with most studies reporting smaller volumes in these regions in PaD individuals compared to controls. However, a handful of studies reported larger volumes in patients than controls, particularly in brainstem nuclei (Del Casale et al., 2013; Del‐Ben & Graeff, 2009; Protopopescu et al., 2006; Sobanski & Wagner, 2017; Uchida et al., 2008; Wang, Cheng, et al., 2020). Larger brainstem volumes in PaD individuals versus controls may explain autonomic features of PaD, but much more work is needed in this area to understand this effect.

Few studies have examined neuroimaging sex differences in PaD, and all have been conducted in very small samples. An earlier VBM study of 24 PaD patients (9 males, 15 females) and 24 matched controls (Asami et al., 2009) reported smaller volumes in several brain regions (amygdala, ACC, STG, insula, cerebellar vermis, and regions of the fronto‐occipital cortex) of the PaD group after adjusting for age, ICV, and SES. Of these regions, males with PaD showed significantly lower volumes than females with PaD in the bilateral insula, right amygdala, and left occipitotemporal gyrus, whereas females with PaD only had smaller volumes than males in the right STG. However, sex‐specific analyses revealed female‐specific effects (PaD females < control females) in the bilateral dorsolateral PFC (DLPFC), ventrolateral PFC, thalamus, parietal cortex, and right cerebellar vermis (Asami et al., 2009).

### 4.5 Obsessive compulsive disorder

OCD is a common psychiatric disorder with a complex phenomenology. Previously characterized as an anxiety disorder, OCD is now recognized by the DSM‐5 as an independent class of obsessive compulsive and related disorders that include OCD, body dysmorphia, hoarding, trichotillomania, excoriation disorder (skin picking), and OCD secondary to medication use or another medical condition (Marras, Fineberg, & Pallanti, 2016). Hallmark features of OCD include persistent and intrusive thoughts, urges, or images (i.e., obsessions), and repetitive and/or rigid behaviors that occur in response to an obsession (NIMH, 2019). OCD is also highly comorbid with other psychiatric conditions, with lifetime comorbidity estimates as high as 92% in population‐based samples (Brakoulias et al., 2017; de Mathis et al., 2013; Ruscio, Stein, Chiu, & Kessler, 2010). The high comorbidity rate and evolving nosology of OCD has complicated efforts to characterize the role of demographic factors in OCD, but most studies show higher lifetime prevalence of adult OCD in females (OR = 1.4–3.0) than males (OR = 0.9–1.0) (Fawcett, Power, & Fawcett, 2020; Kessler et al., 2012). Sex differences in past year prevalence of OCD, however, has been inconsistent across studies (Adam, Meinlschmidt, Gloster, & Lieb, 2012; Castle, Deale, & Marks, 1995; Fawcett et al., 2020; Mathes, Morabito, & Schmidt, 2019; Ruscio et al., 2017). These inconsistencies may be due to the proportion of adolescents surveyed, as OCD is purportedly more common in males than females, whereas the opposite is true in adults (Mathes et al., 2019). In adults over age 65, recent work suggests greater OCD prevalence in males than females (Cath, Nizar, Boomsma, & Mathews, 2017).

Prior work has identified notable sex differences in the expression of OCD symptoms. Females are more likely to have contamination obsessions and preoccupation with things that may harm others compared to males. Thus, females tend to express more cleaning and checking compulsions. Obsessions and compulsions in males are more frequently related to intrusive sexual and religious dimensions, as well as preoccupations with symmetry and order (Torresan et al., 2013). Sex differences in comorbidity profiles also have been reported, with females having significantly higher comorbidity rates than males of any mental disorder (Benatti et al., 2020), with specifically higher comorbidity rates of mood and eating disorders than males. By contrast, males with OCD are more likely to have comorbid alcohol use disorders, psychotic disorders, and developmental disorders than females (Rintala et al., 2017; Torresan et al., 2013).

Neuroimaging hallmarks of OCD include structural alterations in gray matter and white matter of fronto‐striatal, thalamic, and temporolimbic and temporal–parietal networks, as well as the corpus callosum (Boedhoe et al., 2017; Boedhoe et al., 2018; Piras et al., 2015; Piras et al., 2019). In large‐scale studies, including those from our group, the most consistent structural abnormalities in OCD patients include cortical thinning of the DLPFC, transverse temporal, and inferior parietal cortices, smaller hippocampal volumes, and *larger* pallidum volumes relative to controls (Boedhoe et al., 2017; Boedhoe et al., 2018; Fouche et al., 2017; Piras et al., 2019). There is currently little to no evidence of sex differences in neuroimaging indices, but most studies have modeled sex as a covariate rather than predictor variable of interest or have included matched samples of males and females (Hazari, Narayanaswamy, & Venkatasubramanian, 2019). In one small study (Hawco et al., 2017) comparing whole‐brain FA metrics between male (*n* = 17) and female (*n* = 21) OCD patients, males with OCD had higher FA than females, consistent with normative sex differences in brain white matter. Sex‐by‐diagnosis interactions were tested in meta‐ and mega‐analyses of subcortical, cortical, and DTI metrics from the ENIGMA consortium (Boedhoe et al., 2017; Boedhoe et al., 2018; Piras et al., 2019), but these were not significant in any region. Of note, effect sizes of OCD on brain structure are small in general (average Cohen's *d* < 0.2) (Boedhoe et al., 2017; Boedhoe et al., 2018; Piras et al., 2019), which may be due to the high comorbidity rate of OCD. As sex differences in OCD appear to be stronger in relation to symptom dimensions and comorbidity profiles, future neuroimaging studies should examine sex‐specific neuroimaging patterns in relation to these clinical features.

### 4.6 Major depressive disorder

Approximately 4.4% of the global population has been diagnosed with major depressive disorder (MDD) (World Health Organization, 2017), with a twofold greater lifetime risk of MDD in females than males (Rubinow & Schmidt, 2019). Sex differences are also evident in patterns of symptom expression. A recent meta‐analysis by Cavanagh, Wilson, Kavanagh, and Caputi (2017) showed that males with MDD report a higher frequency of risk taking, alcohol/substance misuse, anger, low self‐esteem, and cognitive difficulties compared to women. Conversely, females with MDD are three times more likely to exhibit atypical depression symptoms such as fatigue, increased appetite, and sleep disturbances compared to men, who more frequently demonstrate typical (melancholic) depression symptoms. Females also have a twofold to threefold greater risk for dysthymia and seasonal affective disorder (Rubinow & Schmidt, 2019). Sex differences in functional impairments are also reported, with males showing greater work impairment and females showing greater social impairment.

Neuroimaging hallmarks of MDD include smaller volumes in the hippocampus, amygdala, ACC, DLPFC, medial PFC and OFC, and larger volumes in the ventricles (Dunlop, Talishinsky, & Liston, 2019; Lai, 2019; Lemogne, Delaveau, Freton, Guionnet, & Fossati, 2012). The ENIGMA‐MDD Working Group confirmed these hallmarks in the largest subcortical, cortical, and white matter neuroimaging studies of MDD to date (*N* > 8,000), yet none of these studies detected sex‐by‐diagnosis interaction effects on brain structure (Schmaal et al., 2016; Schmaal et al., 2017; van Velzen et al., 2019). However, separate work by Frodl et al. (2017) and Tozzi et al. (2019) revealed sex‐by‐diagnosis interaction effects on subcortical and cortical brain structure as a function of childhood trauma exposure. In a meta‐analysis of 958 MDD patients (*M*_age_ = 42.4 ± 14.3 years, 64% females) and 2,078 controls (*M*_age_ = 46.3 ± 15.2 years, 48% females), greater severity of childhood trauma corresponded to smaller bilateral caudate volumes in female patients and controls, but this effect was not significant in males (Frodl et al., 2017). In a subsequent meta‐analysis of cortical morphometry in 1,284 MDD patients (*M*_age_ = 40.9 ± 14.6 years, 63.3% females) and 2,588 controls (*M*_age_ = 43.3 ± 15.9 years, 50.3% females), childhood trauma severity was positively associated with cortical thickness in the rostral ACC of males in both patient and control groups, but this effect was not significant in females (Tozzi et al., 2019).

Inconsistent and heterogeneous medication use is another complicating factor for interpreting neuroimaging results in MDD samples, as medication use is often inconsistently measured across studies. An earlier study of 29 medication‐naive MDD patients (55% female; *M*_age_ = 29.5 ± 6.8 years) and 33 controls (51.5% female; *M*_age_ = 29.9 ± 8.3 years) revealed significant sex‐by‐diagnosis interactions in the PFC, amygdala, hippocampus, and caudate (Kong et al., 2013). Compared to controls, females with MDD had lower gray matter density in the amygdala and hippocampus, whereas males with MDD had lower gray matter density in the striatum compared to controls. Recent work from the International Study to Predict Optimized Treatment in Depression (Saveanu et al., 2015) used “fixel‐based” analysis (FBA) to identify microstructural white matter predictors of treatment remission after 8 weeks of antidepressant treatment in 221 medication‐naive MDD patients and 67 controls, aged 18–65 years (Lyon et al., 2019). The FBA technique measures individual fiber bundle elements (with different orientations) within an image voxel (i.e., fixel) to measure the degree and density of crossing fibers in white matter tracts (Raffelt et al., 2015; Raffelt et al., 2017), which describe more than 90% of white matter voxels (Jeurissen, Leemans, Tournier, Jones, & Sijbers, 2013). FBA produces three key metrics based on the fiber orientation distribution: fiber density (FD),^2^ fiber cross section (FC),^3^ and fiber density cross section (FDC)^4^; in which lower values of all three metrics represent decreased white matter microstructure. Sex‐stratified analyses by Lyon et al. (2019) showed that females with MDD (*n* = 115) had ~10% lower FDC (the product of fiber density and FC) in the *genu* of the corpus callosum compared to healthy controls (*n* = 34), whereas males with MDD (*n* = 106) showed ~11% lower FDC in the right anterior limb of the internal capsule (ALIC) compared to controls (*n* = 33) independent of treatment outcome. Additionally, compared to female controls, females with MDD had on average 5–8% lower FC in clusters containing the corpus callosum, right ALIC, *tapetum*, and ILF; males with MDD exhibited ~7% lower fiber cross section in the right ALIC only. Across both sexes, lower fiber cross section in the *tapetum* predicted decreased likelihood for remission after antidepressant treatment (Lyon et al., 2019). As the *tapetum* is the major connector between the left and right hippocampus, these findings align with existing studies by our group and others implicating the hippocampus as a common site for brain disruption in MDD.

Finally, advanced age may also affect sex differences in clinical and neuroimaging phenotypes of MDD. In a recent analysis of 610 community‐dwelling older adults (ages 67–74, 52.5% females), sex‐by‐lifetime MDD interactions were significant in the amygdala, caudate, and rostral ACC (Ancelin et al., 2019). Lifetime MDD was associated with a 4 and 7% reduction in the amygdala and caudate in males respectively, with no significant effect in females. By contrast, lifetime MDD in females was associated with 6% larger volume in the rostral ACC compared to healthy controls, with no significant effect in males. Interestingly, the proportion of females with both current and lifetime MDD was significantly higher than in males, but antidepressant use and prevalence of hypertension were significantly higher and lower (respectively) in females than in males (Ancelin et al., 2019).

### 4.7 Bipolar disorder

Bipolar disorder (BD) is a manic‐depressive mental health condition characterized by severe mood shifts that interfere with daily life (The National Institute of Mental Health, 2020). The age‐standardized 12‐month prevalence rate of BD is around 0.7% of the world population—a rate that has remained stable since 1990 (Ferrari et al., 2016). There are two primary subtypes of BD; BD‐I is “traditional” BD, which involves manic and depressive periods that generally last at least 7 days and 2 weeks, respectively. BD‐II also involves mood fluctuations but with less severe manic episodes (hypomanic). The prevalence of BD‐I is generally consistent between males and females (Grant et al., 2009; Szádóczky, Papp, Vitrai, Ríhmer, & Füredi, 1998), but subtle differences have been reported in patients from Ethiopia (*N* = 68,378; lifetime prevalence in males = 0.6%, females = 0.3%) (Negash et al., 2005) and Hungary (*N* = 2,953; lifetime prevalence in males = 1.3% males, females = 1.6%) (Szádóczky et al., 1998). Several studies show higher prevalence of BD‐II in females than males, again with some inconsistencies (Szádóczky et al., 1998). The global prevalence estimate of BD is approximately 0.6% (both types), which is generally consistent with the United States (James et al., 2018).

There are also sex differences in symptom patterns and treatment response. Females show increased frequency of hypomania and more rapid cycling between manic and depressive states. Atypical depression symptoms are more common in BD females, and there is a higher risk for manic‐depressive cycles during menopause and around childbirth, which has been attributed to intense hormonal fluctuations (López‐Zurbano & González‐Pinto, 2019; Sit, 2004). Females are more frequently treated for BD with typical psychotropic medication (antidepressants, benzodiazepines) and cognitive behavioral therapy, whereas males are more commonly treated with lithium (López‐Zurbano & González‐Pinto, 2019).

Small‐scale neuroimaging studies have reported interactions between sex and BD in the ventricles, caudate, hippocampus, amygdala, nucleus accumbens, OFC, PFC, occipitotemporal cortex, anterior pituitary, cerebellar vermis, and temporal lobe asymmetry (for review, see Jogia, Dima, & Frangou, 2012), but across studies there is no clear direction of male–female differences among BD patients, likely due to small sample sizes (*n*'s < 100). In larger samples, however, a meta‐analysis of subcortical brain structures in 1,710 BD patients (59.1% females) and 2,594 controls (55.4% females) from the ENIGMA‐BD Working Group (Hibar et al., 2016) revealed larger thalamus volumes in females (vs. males) with BD. The ENIGMA‐BD cortical analysis (Hibar et al., 2018) did not reveal significant sex differences in the effect of BD on cortical thickness or surface area in BD patients over age 25, but significant sex‐by‐diagnosis interactions were observed in patients under age 25 (*n* = 411, *M*_age_ = 21.1 ± 3.1). Specifically, young females with BD had less cortical thinning than would be expected by sex and diagnosis alone in the right *pars triangularis* (*d* = 0.26), right superior frontal gyrus (*d* = 0.19), left insula (*d* = 0.20), and left temporal pole (*d* = 0.20) than males. The authors noted that these sex differences may reflect a normative sexual dimorphism in cortical development where cortical thickness is larger in females than males, though these interactions were not observed at older ages. Sex differences in cortical surface area were not detected. In a separate recent large‐scale meta‐analysis of adult BD (*N* = 50 neuroimaging studies; 1,843 BD patients, 2,289 controls), the proportion of females with BD (54.7%), and specifically BD‐I (55.1%), correlated negatively with volume in the insula (Wang et al., 2019).

These results and the general variability of sex‐by‐diagnosis interactions in the BD literature call for longitudinal studies to better understand the trajectory of brain alterations in males and females with BD; such large‐scale longitudinal studies are now underway in the ENIGMA Bipolar Working Group. Additionally, many studies do not specify BD subtypes, so it is unclear whether more pronounced sex differences in brain structure would emerge when accounting for subtype variance, particularly as BD‐II has stronger sex differences in disease prevalence than BD‐I.

### 4.8 Schizophrenia

SCZ is a serious mental health disorder, broadly characterized by disorganized thoughts, beliefs, and behaviors (National Institute of Mental Health, 2016). Although commonly described as a developmental condition, symptoms do not typically present until the third decade, with an incidence rate approximately 40% higher in males than females (McGrath et al., 2004; Seeman, 2013), though these numbers have been debated. SCZ also may be subdivided into early‐onset and late‐onset cases, with even greater sex disparities in late‐onset cases. Symptom onset is generally earlier in males than females for young adult cases (Li, Ma, Wang, Yang, & Wang, 2016), but females make up around 66–87% of late‐onset cases after age 40, with more positive symptoms than men, particularly sensory hallucinations and persecutory delusions (Lindamer, Lohr, Harris, McAdams, & Jeste, 1999). Across all ages, males with SCZ tend to be less responsive to antipsychotic medications and have more frequent hospitalizations than females. The prognosis of SCZ is also generally better in females than in males, with higher rates of recovery and remission as well as better preserved interpersonal relationships, employment and marriage retention (Grossman, Harrow, Rosen, & Faull, 2006; Grossman, Harrow, Rosen, Faull, & Strauss, 2008).

The most prominent neuroimaging markers of SCZ are enlarged ventricles and volume deficits in the hippocampus, though these hallmarks were initially established in predominantly male samples (Exner et al., 2008). Indeed, earlier work in small samples (*N* < 100) reported larger ventricles and smaller volumes in the frontal (Narr et al., 2001) and temporal (Bryant, Buchanan, Vladar, Breier, & Rothman, 1999; Narr et al., 2001) lobes in SCZ males than females, and greater volume reductions (Takayanagi et al., 2011) and leftward asymmetry in the amygdala (Niu et al., 2004). Other small‐scale studies reported smaller ACC and insula volumes in SCZ females compared to healthy controls (Duggal, Muddasani, & Keshavan, 2005; Goldstein et al., 2002; Takahashi et al., 2002), but differences were not significant in males. More recently, Womer et al. (2016) revealed smaller cerebellar vermis volume in a matched sample of male SCZ patients (*n* = 24, *M*_age_ = 31.0 ± 10.6) compared to male controls (*n* = 24, *M*_age_ = 33.7 ± 8.9), but no significant effect in females (*n*_SCZ_ = 26, *M*_age_ = 30.7 ± 10.5; *n*_Controls_ = 30, *M*_age_ = 32.0 ± 12.0). du Plessis et al. (2020) identified a three‐way interaction between sex, first episode SCZ, and childhood trauma on hippocampal subfield volumes extracted with the FreeSurfer‐v.6.0 hippocampal subfield protocol (Iglesias et al., 2015), such that female SCZ patients (*n* = 21) had a larger hippocampal fissure than female controls (*n* = 35), male SCZ patients (*n* = 58), and male controls (*n* = 47) who were matched for age (*M*_age_ = 23 ± 7). However, further work is needed to determine the reliability of these findings given the small sample size and the fact that the fissure is prone to noise given its small size and proximity to the ventricles.

White matter microstructure is also impacted in SCZ, potentially in a sex‐specific manner. Recently, Kelly et al. (2018) conducted the largest‐ever meta‐analysis of white matter diffusion in 1,963 SCZ patients (ages 18–86 years) and 2,386 controls (ages 18–77 years) on behalf of the ENIGMA‐SCZ Working Group. Main analyses revealed a widespread pattern of lower FA in SCZ patients, but sex‐by‐diagnosis interactions were not detected. Sex‐specific analyses, however, revealed lower FA in SCZ females (*n* = 671) than female controls (*n* = 1,090) in 20 of 25 white matter tracts, whereas lower FA in SCZ males (*n* = 1,292) was only observed in 14 tracts compared to male controls (*n* = 1,296); female‐specific significant effects were observed in the superior *corona radiata*, uncinate fasciculus, IFOF, hippocampal segment of the cingulate gyrus, and internal capsule. Similar to Kelly et al. (2018), other work by the ENIGMA‐SCZ Working Group (van Erp et al., 2018) revealed nonsignificant sex‐by‐diagnosis interactions on cortical thickness or surface area, but sex‐specific analyses were not tested.

Recently, machine learning methods were used to identify voxel‐based neuroimaging phenotypes across 157 SCZ patients (van Erp et al., 2018, 24.8% females) and 169 controls (van Erp et al., 2018, 31.4% females) when the diagnostic label was hidden (Honnorat, Dong, Meisenzahl‐Lechner, Koutsouleris, & Davatzikos, 2019). A semisupervised clustering method (CHIMERA) revealed three distinct clusters: Clusters 1 and 2 consisted of more than 80% males, but Cluster 3 (*n* = 52) had a mixed sex distribution (56% male). Pairwise comparisons revealed less CSF expansion and lower white matter, total brain, and ICVs in Cluster 3 compared to other clusters, with less frontal and temporal atrophy (Honnorat et al., 2019). While these methods need to be tested in larger samples, results appear to align with findings from Kelly et al. (2018), Bryant et al. (1999), and Narr et al. (2001), suggesting greater white matter disruption with relatively preserved frontal‐temporal gray matter in female SCZ patients. It is unclear whether this phenotype is a cause or consequence of SCZ.

### 4.9 Substance use disorders

Prevalence rates of substance use disorders (SUDs) tend to be higher in males for most substances, but sex differences in overall SUD prevalence is narrowing as the number of females with SUDs increases (Keyes, Grant, & Hasin, 2008; McHugh, Votaw, Sugarman, & Greenfield, 2018; Seedat et al., 2009). While prevalence rates for SUDs are generally higher in males, the health impact of SUDs is more detrimental in females for most (but not all) substances of abuse and dependence (McHugh et al., 2018). Specifically, females with SUDs have higher risk of alcohol‐induced injuries, liver damage, tobacco‐induced heart and lung disease, and increased mortality (Agabio, Campesi, Pisanu, Gessa, & Franconi, 2016). Females also show greater negative side effects from marijuana, cocaine, and heroin use, and metabolize nicotine more quickly than males. Among males and females who consume equal amounts of alcohol, females show increased risk for alcohol toxicity, cognitive impairment, sleep disturbance, and intoxication (Franconi & Campesi, 2014).

Several papers describe sex differences across all phases of substance use and addiction (Becker & Hu, 2008; Bobzean, DeNobrega, & Perrotti, 2014; Keyes et al., 2008; McClellan, Reed, & Becker, 2017). McClellan et al. (2017) recently detailed faster escalation of drug use, maintenance of drug use at higher doses, greater negative affect and stress during withdrawal, and greater likelihood of relapse in females. These differences may be due to a sexually dimorphic reward system that differentially reinforces substance use behaviors between females and males (Fattore, Melis, Fadda, & Fratta, 2014). Accordingly, motivations for substance use also differ by sex, with females engaging in substance use to reduce stress and negative emotions, and males engaging in substance use to increase positive emotions (Glavak Tkalić, Sučić, & Dević, 2013). In studies of tobacco use, females show faster nicotine metabolism than males, which is linked to higher levels of estrogen and the metabolic liver enzyme cytochrome P450 2A6 (CYP2A6) (Benowitz, Lessov‐Schlaggar, Swan, & Jacob III, 2006; Franconi, Sanna, Straface, Chessa, & Rosano, 2012). Additionally, women taking oral contraceptives, particularly estrogen‐only, show faster nicotine clearance than women on progesterone‐only contraceptives or not taking any contraceptives (Benowitz et al., 2006). Interestingly, sex differences in nicotine clearance do not exist between men and postmenopausal women, suggesting an important and specific role of estrogen in nicotine metabolism. Similar to AUD, women exhibit greater health consequences of nicotine than men, specifically in risk for coronary heart and lung disease (Greenfield, Back, Lawson, & Brady, 2010; Huxley & Woodward, 2011).

Treatment‐seeking behaviors and treatment success for SUDs also differ by sex. A report from the Substance Abuse and Mental Health Services Administration (Substance Abuse and Mental Health Services Administration, 2016) showed that significantly more males than females were admitted to a treatment facility for misuse of alcohol and marijuana (72% males), heroin (66% males), and nonsmoked cocaine (69% males). By contrast, admissions for misuse of crack, methamphetamines/amphetamines, and opiates other than heroin were more proportional between sexes (Substance Abuse and Mental Health Services Administration, 2016). Interestingly, 82% of admissions for sedative misuse (barbiturates, benzodiazepines, etc.) were among White, non‐Hispanics, with significantly higher admissions among females (50%) than males (32%). This is perhaps not surprising as benzodiazepines are the hallmark treatment for anxiety disorders, which are significantly more common in females than males. Indeed, a recent study of benzodiazepine use and misuse in 349 adults (*M*_age_ = 39.2 ± 13.0) revealed a lifetime benzodiazepine prescription in 58% of females compared to 44% of males (*p* < .01) (McHugh et al., 2021). While the proportion of individuals who misused these prescriptions did not differ significantly by sex, 68% of females (vs. 49% of males) attributed prescription misuse to coping motives, whereas 40% of males (vs. 19% of females) attributed misuse to drug enhancement. Further, females with a lifetime history of benzodiazepine misuse reported significantly higher levels of drug craving compared to males (McHugh et al., 2021).

The neuroimaging literature on sex differences in SUDs is complicated, as brain signatures differ based on the substance of abuse/dependence. In individuals with cocaine dependence, Rando, Tuit, Hannestad, Guarnaccia, and Sinha (2013) used VBM to identify sex differences in gray matter volume in cocaine‐dependent individuals who were completing an in‐patient treatment program and had been abstinent at least 3 weeks prior to the imaging scan (*N* = 36) compared to controls. Results revealed lower gray matter volume in the left IFG, insula, STG, and hippocampus of cocaine‐dependent females (*n* = 18, *M*_age_ = 36.7 ± 5.6) compared to female controls (*n* = 22, *M*_age_ = 31.5 ± 8.3), whereas cocaine‐dependent males (*n* = 18, *M*_age_ = 38.2 ± 5.4) had lower gray matter volume in the precentral gyrus and mid‐cingulate compared to male controls (*n* = 28, *M*_age_ = 30.9 ± 9.7). More recently, the ENIGMA‐Addiction Working Group revealed significantly lower gray matter volume in the left anterior insula and lingual gyrus in cocaine‐dependent females (*n* = 70, *M*_age_ = 39.6 ± 7.6) compared to female controls without a history of substance dependence or other disorders (*n* = 70, *M*_age_ = 37.2 ± 9.8), and after adjusting for age, education, project site, and ICV (Rabin et al., 2020). There were no significant differences between cocaine‐dependent males (*n* = 140, *M*_age_ = 37.8 ± 6.7) and male controls (*n* = 140, *M*_age_ = 37.0 ± 8.5), but within‐group analyses showed that hippocampal volume was negatively associated with the duration of cocaine use in cocaine‐dependent males, but not in cocaine‐dependent females. These results did not change when adjusting for current tobacco use and alcohol use disorder.

Problematic alcohol use is also associated with sex differences in microstructural white matter and substructures of the limbic system. A study of 303 heavy alcohol users (ages 21–56, 30% females) used DTI to determine the effect of problematic alcohol use and alcohol drinking frequency on five white matter tracts (CC, fornix, external capsule, superior longitudinal fasciculus [SLF], cingulum) that were previously associated with heavy drinking (here defined as ≥4 heavy drinking episodes per month in females and ≥5 episodes per month in males) (Monnig et al., 2015). The authors used confirmatory factor analysis and structural equation modeling to create a latent “white matter factor” based on FA values of the five tracts of interest. Sex significantly moderated the effect of the proportion of drinks per day on the white matter factor, such that a higher number of drinks per day was associated with lower FA in the white matter factor in females, but not males (Monnig et al., 2015). Interestingly, a more recent voxelwise study of the effects of heavy alcohol use history on white matter microstructure revealed a somewhat different pattern of effects by sex (*N* = 90, ages 23–76) in abstinent adults who met DSM‐IV criteria for lifetime alcohol use or dependence disorder (minimum heavy drinking history of 5 years). Specifically, alcoholic males who were abstinent at the time of scan (*n* = 23) had lower FA than nonalcoholic male controls in the CC, SLF, arcuate fasciculus and external capsule, whereas abstinent alcoholic females (*n* = 26) had higher FA in these regions compared to female controls (Sawyer et al., 2018). While these results seem inconsistent with those by Monnig et al. (2015), these studies cannot be truly compared given that participants in Sawyer et al. (2018) had been sober for an average of 4–9 years whereas participants in the former study were not abstinent. Further, although both studies focused on the same tracts of interest, a direct comparison cannot be made between tract‐specific FA outcomes and an aggregate measure of tract FA (i.e., white matter factor) as examined in Monnig et al. (2015). Several other methodological components may have also contributed to the seemingly divergent study outcomes including differences in sample size, sample composition, and operational definitions for “heavy drinking”.

A recent substructural analysis (*N* = 131, ages 23–76, 46.9% females) from the same group showed that a longer duration of alcohol sobriety predicted larger volumes in the hippocampal CA1 region for males, but smaller CA1 volumes in females (Sawyer et al., 2020). Finally, the most recent work from ENIGMA‐Addiction examined neuroimaging sex differences in the effect of alcohol dependence on subfield volumes of the hippocampus and amygdala (Grace et al., 2021). Subfields were identically processed and QC'd across 10 cohorts using the FreeSurfer‐v.6.0 pipeline for 643 individuals with alcohol dependence (35% females) and 323 healthy controls (30.3% females) without alcohol dependence or other psychiatric disorders. Results revealed significant main effects of sex and sex‐by‐diagnosis interactions in a priori ROI of the whole bilateral amygdala and basolateral subregion, but not the central amygdala. Specifically, alcohol‐dependent males (*n* = 418) had 5% and 3% lower volumes in these regions (respectively) compared to male controls (*n* = 225) after adjusting for age, education, ICV, and tobacco use. These regions did not differ significantly between female cases (*n* = 225) and controls (*n* = 98). An exploratory analysis of sex differences in the accessory basal, anterior and cortico‐amygdaloid nuclei also revealed significant sex‐by‐diagnosis interactions, with larger volumes in males than females with alcohol dependence. Sex‐by‐diagnosis interactions in hippocampal subfields were not significant in any a priori defined region (CA1, CA3, subiculum, DG), but analyses of exploratory subfields revealed significant interactions in the bilateral HATA and right fimbria, with 11 and 8% lower volumes in alcohol‐dependent males than females, respectively, after adjusting for the same covariates (Grace et al., 2021).

Finally, preliminary work from the ENIGMA‐Addiction Working Group showed a significant role of sex on the effects of cannabis dependence (*N* = 270, 31.1% female, *M*_age_ = 26.91 ± 9.66) in the cerebellum and OFC (Rossetti et al., 2019). Specifically, females with cannabis dependence showed smaller white matter volume in the cerebellum and a thinner OFC compared to female nonaddicted cannabis users and female controls. Cannabis use status was not associated with imaging outcomes in males, but males with higher monthly cannabis use and earlier cannabis use onset had lower cerebellar gray and white matter volume, respectively (Rossetti et al., 2019). Other studies have identified sex differences in neural circuitries underlying SUDs using resting state functional connectivity, and these have been reviewed elsewhere (Hamidullah, Thorpe, Frie, Mccurdy, & Khokhar, 2020; Rakesh, Allen, & Whittle, 2020).

Sex differences in comorbid and multimorbid SUD profiles further complicate the interpretation of sex effects on the brain, particularly for substances of abuse/dependence with opposing mechanisms of action or prescription medications designed to treat a separate psychiatric condition. Indeed, McCabe, West, Jutkiewicz, and Boyd (2017) showed that most past‐year nonalcohol SUDs co‐occurred with another SUD, with a greater likelihood of multiple SUDs in males than females. These factors illustrate the complexities of SUD research, and future neuroimaging studies may benefit from the use of sophisticated machine learning algorithms in large and diverse cohorts to model these complex interactions with precision.

## 5 SEX DIFFERENCES IN NEURODEGENERATIVE DISORDERS

The role of biological sex in neuropsychiatric conditions is relevant for understanding the trajectory of brain health across the lifespan, as all of the abovementioned conditions have been linked to an “accelerated” profile of aging using neuroimaging (Hajek et al., 2019; Han et al., 2019; Koutsouleris et al., 2014; van Gestel et al., 2019) and epigenetics (Fries et al., 2017; Luo et al., 2020; Wolf et al., 2017). While these studies use algorithmic tools to determine patterns that appear consistent with “accelerated aging,” it is speculative at best to conclude that biological patterns that appear older than expected for a person's chronological age are representative of true biological aging—a phenotypic concept that has no true measurement (Butler et al., 2020; Freund, 2019). Still, each of the psychiatric conditions discussed in this review has been linked to increased risk for dementia, suggesting an important role for mental distress as an etiological predictor of neurodegenerative disease. In the sections below, we describe the most common neurodegenerative conditions and review the existing evidence for sex effects on disease prevalence, symptom patterns, and neuroimaging signatures. As with the previous section, conditions are presented in order of the magnitude of the observed sex difference in disease prevalence.

### 5.1 Parkinson's disease

Parkinson's disease (PD) is a neurodegenerative movement disorder characterized by symptoms of resting tremor, bradykinesia, rigidity, gait, and speech abnormalities; cognitive systems are also affected (Podcasy & Epperson, 2016). Classic biomarkers of PD include loss of dopaminergic neurons in the *substantia nigra* and basal ganglia, as well as cell loss in the *nucleus basalis* of Meynert—the major cholinergic projection to the cortex (Benazzouz, Mamad, Abedi, Bouali‐Benazzouz, & Chetrit, 2014; Zarow, Lyness, Mortimer, & Chui, 2003). The first‐line treatment for PD includes levodopa or carbidopa, which promote dopamine synthesis from tyrosine. Unfortunately, an estimated 50–70% of dopaminergic neurons are lost by the time a diagnosis is made, reducing the potential efficacy of pharmacotherapies (Cheng, Ulane, & Burke, 2010).

The prevalence of PD is higher in males and typically diagnosed 2 years earlier than in females (Haaxma et al., 2007; Taylor, Cook, & Counsell, 2007). Males with PD report more sleep disruption, sexual dysfunction, urinary incontinence, and symmetrical upper body symptoms than females. Males also exhibit more severe cognitive impairment including emotion recognition compared to females, and are more likely to progress to dementia (Cerri, Mus, & Blandini, 2019; Cholerton et al., 2018). Female‐dominant symptoms include greater postural problems and poorer motor performance on the Unified PD Rating Scale (Cerri et al., 2019; Fahn et al., 2008)—a common measure of clinical function in PD patients. Females also tend to report more nonmotor symptoms than males, including increased fatigue, apathy, cardiovascular symptoms, anhedonia, sensory dysfunction, constipation, sweating, and pain (Martinez‐Martin et al., 2012; Solla et al., 2012).

Across all patients with PD, neuroimaging studies show lower gray matter volume in the basal ganglia, motor cortex, and cerebellum and lower FA in the substantia nigra of PD patients compared with controls (Berman & Miller‐Patterson, 2019; Geng, Li, & Zee, 2006; Kang et al., 2015; Zhang et al., 2015). In recent work by the ENIGMA‐PD Working Group, individuals with PD (*n* = 2,367, *M*_age_ = 63.4 ± 9.8, 36% females) showed widespread cortical thinning in parietal association areas, primary and supplementary motor areas, inferior temporal cortex, precuneus, and PCC, and smaller volumes in the bilateral putamen, hippocampus, amygdala and accumbens compared to healthy controls (*n* = 1,183, *M*_age_ = 59.4 ± 12.3, 46% females) (Laansma, Bright, Al‐Bachari, & Anderson, 2020), but sex‐by‐diagnosis interactions were not significant.

Despite reported sex differences in PD clinical presentation and symptom expression, there is almost no literature devoted to sex differences in structural brain metrics in PD. In one of the only studies we could find, Tremblay et al. (2020) used DTI and deformation‐based morphometry (DBM—an alternative to VBM that maps regional atrophy patterns and has heightened sensitivity to subcortical brain atrophy (Scanlon et al., 2011))—to examine sex‐specific brain atrophy maps and structural connectivity patterns between 232 PD patients (36% females) and 117 healthy controls (33% females) participating in the Parkinson's Progression Markers Initiative (PPMI). “W‐score” (covariate adjusted Z‐scores) maps (Jack Jr et al., 1997; La Joie et al., 2012) were created to obtain cross‐sectional estimates of regional brain atrophy relative to controls after accounting for age and sex. Here, the W‐score indicated the normal deviation in a PD patient's brain metric relative to the expected value in controls. A total of 246 cortical and subcortical regions were analyzed using the parcellation from the Brainnetome atlas (Fan et al., 2016). Results showed lower cortical gray matter volume and higher intracerebral CSF in females than males with PD after adjusting for ICV. There were no sex differences in cortical thickness, but DBM analyses revealed greater brain atrophy overall in males than females with PD, despite comparable levels of disease severity and duration (mean age of onset was 63.9 years for both sexes). Regionally, PD males had a stronger pattern of atrophy in frontal‐subcortical structures (thalamus, two regions of the left insula, six regions of the frontal lobe) compared to PD females, whereas PD females had a stronger atrophy pattern in the posterior cortex (superior parietal, right occipital lobe) and three regions of the frontal cortex. Structural connectivity analyses also showed lower local network efficiency in males than females with PD in ~45% of the 246 ROIs, and efficiency in two regions was associated with cognitive performance in males only. Specifically, local efficiency in the right IFG was positively associated with delayed recall on the Hopkins Verbal Learning Test (HVLT), and efficiency in the ventral agranular insula was positively associated with scores on the Letter Number Sequencing task (*r*'s = .42). DBM measures were not associated with clinical or cognitive outcomes in males or females.

The major takeaway of the abovementioned study was that males with PD had greater brain disruption than females, with more widespread sex differences in local white matter network efficiency than brain atrophy. Lower network efficiency indicates less efficient information transfer between white matter networks (Bullmore & Sporns, 2009; Sporns, 2018), and has been recently linked to age‐related cognitive difficulties on tests of memory, executive function, and attention—domains that are tapped by the HVLT and LNS. As several studies show that microstructural white matter degeneration precedes brain atrophy in normal aging, the degree to which local efficiency metrics are a marker of sex differences in PD versus normal aging are unclear, even with the use of W‐scores. Further, the beta weights for each of the significant sex effects appeared to be stronger in DBM (absolute *β*'s = .39–.72) than connectivity (absolute *β*'s < .001) metrics. While these differences may be due to a lack of standardized beta scaling, these methods are not described and the effect sizes were not interpreted (Tremblay et al., 2020).

Other DTI work in the PPMI cohort (Burciu et al., 2017) has shown that longitudinal changes in the degree of free water in the posterior substantia nigra (PSN) is a marker of PD progression using Pasternak's free water method (Pasternak, Sochen, Gur, Intrator, & Assaf, 2009), and that sex is a strong predictor of PSN free water over a 4‐year period (*M*_age_ at baseline = 59.1 ± 9.7). Specifically, the 4‐year change in PSN free water was significantly greater in males (*n* = 34) than females (*n* = 12) with PD, and annual changes in PSN free water were significantly associated with disease progression on the widely used Hoehn and Yahr scale. However, these results should be interpreted with caution given the very small sample size and imbalanced sex ratio in this study (Burciu et al., 2017).

### 5.2 Multiple sclerosis

MS is a chronic and debilitating neuroinflammatory condition that affects approximately 2.8 million people worldwide (Hauer, Perneczky, & Sellner, 2020). MS is the most common neuroimmune condition in young adults, with a diagnosis typically occurring between ages 20 and 50, and average onset around age 32 in high‐income countries of North America, Western Europe, and Australia (Wallin et al., 2019; Walton et al., 2020) (Hauer et al., 2020). MS is twice as common in females than males worldwide, with regional sex ratios as high as 4:1 (Walton et al., 2020). In the United States, females account for over 70% of the 409,217 MS cases that were reported in 2019 (Institute for Health Metrics and Evaluation (IHME), 2019).

MS consists of four primary clinical subtypes. Relapsing–remitting MS (RRMS) is the most common MS subtype (~85% of cases) and is characterized by intermittent neurologic symptoms (relapse) followed by partial or complete remission (Luchetti et al., 2018). Secondary progressive MS (SPMS) is the second most common form of MS that develops after an initial period of RRMS (often during the fifth decade) and is characterized by chronic worsening of neurologic symptoms (Tutuncu et al., 2013). Primary progressive MS (PPMS) is the most aggressive form of MS and characterized by chronic worsening of symptoms from the point of onset (Kantarci, 2019). Finally, progressive‐relapsing MS is the least common form of MS, in which patients experience chronic worsening of symptoms punctuated by periods of remission (Kalincik et al., 2013). RRMS is significantly more common in females than males (risk ratio = 1.08), with a 17.7% higher relapse rate in females (Kalincik et al., 2013). By contrast, males typically exhibit more severe clinical symptoms, faster disease progression, and younger age of conversion from RRMS to SPMS than females (Golden & Voskuhl, 2017). Sex differences in the prevalence of PPMS have not been reported, but male sex is a significant predictor of PPMS following a radiologically isolated syndrome (i.e., asymptomatic white matter lesions) (Kantarci, Lebrun, et al., 2016).

The neuropathological footprint of MS is heterogeneous across individuals, but traditional characteristics include neuroinflammation, enlarged ventricles, whole brain atrophy and regional atrophy of the medial temporal lobe, thalamus, and deep gray matter, and demyelinating lesions that typically affect white matter in the optic nerve, brainstem, basal ganglia, spinal cord, and fiber tracts proximal to the lateral ventricles (Cortese, Collorone, Ciccarelli, & Toosy, 2019; Filippi et al., 2019; Lassmann, Brück, & Lucchinetti, 2007; Reynolds et al., 2011). Cortical lesions are also detected in over 70% of patients with SPMS and over 60% of patients with RRMS (Calabrese et al., 2010). Neuroimaging is essential for visualizing MS brain abnormalities in vivo. As MS has historically been considered a white matter disease, we were surprised to find that only two studies had examined sex differences in white matter microstructure using DTI. In the earlier of these two studies, Schoonheim et al. (2014) examined the relationship between voxelwise DTI metrics (FA, mean [MD], radial [RD], and axial [AxD] diffusivity) and cognitive performance in 131 MS patients (67.2% females) approximately 6 years after diagnosis and 49 age‐matched healthy controls (59.2% females). Most participants had RRMS (*n* = 114, 79 females), followed by SPMS (*n* = 9, 6 females) and PPMS (*n* = 8, 2 females), but subtype was not included in the statistical model. After adjusting for age and education, a generalized linear regression showed significantly lower FA, and higher MD, AxD, and RD in the CC, temporal white matter, and posterior periventricular regions in both male and female patients compared to controls. Male patients also had lower FA and higher diffusivity in the posterior CC, thalamus, cerebellum, pons, and fronto‐parietal white matter compared to male controls. Case–control differences in voxelwise DTI metrics were larger and more expansive in male than female patients, and male patients performed worse on a series of cognitive tasks compared to controls and female patients, but no significant case–control differences in cognitive performance were observed in females (Schoonheim et al., 2014). A longitudinal study by Klistorner et al. (2018) used DTI, T1‐, and T2‐weighted scans to examine microstructural changes in brain lesions over an average of 3.5 years (range = 36–50 months) in 43 consecutive RRMS patients (no relapse; 55.8% female, *M*_age_ = 42.1 ± 6.1) and 20 controls (60% female, *M*_age_ = 41.0 ± 9.1). Results showed worsening of white matter microstructure within the lesion core of all participants over time, as well as increased lesion volume across the whole brain and increased volume in the lateral ventricle. A significant sex difference was observed for the magnitude of MD change in the lesion core, with males showing an increase in MD nearly twice as large as that in females at the follow‐up visit (Klistorner et al., 2018). Taken together, these two studies align with the clinical and neuropathological literature showing greater disease severity and progression in males than females with MS on indices of white matter microstructure. Given all that is known about the neuroimmunology of MS and the vulnerability of white matter microstructure to inflammation, there is an urgent need for additional DTI and beyond‐tensor studies of sex differences in white matter microstructure in MS, particularly in large samples within and across MS subtypes.

While the DTI literature on MS sex differences is sparse, several structural MRI studies have examined sex differences in volumetry and atrophy patterns in MS patients. In an earlier cross‐sectional study of subcortical and lesion volume differences between 120 RRMS patients and 50 healthy controls (Schoonheim et al., 2012), male (*n* = 40, *M*_age_ = 40.4 ± 9.0) and female (*n* = 80, *M*_age_ = 39.6 ± 8.3) patients exhibited lower volumes in all subcortical regions except the bilateral hippocampus in males, and left hippocampus and bilateral putamen in females when compared to controls. Within the patient group, males had significantly lower volumes than females in the bilateral caudate and putamen, with typically larger effect sizes than females. Lesion volumes did not differ significantly between male and female patients, but in male patients only, larger lesion volume was associated with lower cognitive performance on test scores averaged across seven domains (executive functioning, verbal memory, processing speed, visuospatial memory, working memory, attention, psychomotor speed). More recent cross‐sectional work using VBM, however, showed a somewhat different pattern of sex effects between patients and controls in subcortical volumes (Sanchis‐Segura et al., 2016; Voskuhl et al., 2020). Similar to Schoonheim et al. (2012) and Voskuhl et al. (2020) (79 RRMS, 10 SPMS, 45 controls, ages 18–69), Sanchis‐Segura et al. (2016) (56 RRMS, 63 controls, ages 18–61) reported smaller thalamus volumes in both male and female MS patients compared to controls, as well as smaller putamen volumes specifically in male patients than male controls. In contrast to Schoonheim et al. (2012), case–control differences in other subcortical structures were not detected. Surprisingly, neither Voskuhl et al. (2020) nor Sanchis‐Segura et al. (2016) offered any potential explanation for these discrepant studies despite the very limited number of MS studies that have examined sex effects.

Longitudinal studies have also revealed sex differences in atrophy patterns in MS patients. Specifically, Rojas, Patrucco, Besada, Funes, and Cristiano (2013) compared sex differences in total and regional brain volumes, lesion load, and changes in brain volume over a 6‐year period in 45 RRMS patients who were within 60 days of their first demyelinating episode at the baseline visit. Atrophy patterns were measured using the SIENA software from FSL (https://fsl.fmrib.ox.ac.uk/fsl/fslwiki/SIENA) (Smith et al., 2002). Sex differences in imaging outcomes were not significant at baseline, but after 6 years, males (*n* = 20, *M*_age_ = 34.2 ± 1.1) showed a sharper decrease in TBV and total gray matter and sharper increase in lesion volume and annual percentage of global brain atrophy compared to females (*n* = 25, *M*_age_ = 33.5 ± 1.6). Sex differences in the distribution of atrophy patterns were also significant, with males showing a more diffuse global atrophy pattern than females, and females showing more localized atrophy in subcortical frontal lobe areas than males (Rojas et al., 2013).

Finally, sex differences in neuroimaging phenotypes of MS also differ by age, as older age is linked to greater disease severity and disability burden (Zeydan & Kantarci, 2020). In a recent lifespan study of brain volume trajectories of 2,199 MS patients (75.1% female, *M*_age_ = 46 ± 11.6), significant age‐by‐sex interactions were observed for volumes in the lateral ventricle and total normalized gray matter (adjusted for head size using FSL's SEINAX). Post hoc analyses conducted in 10‐year age bands (ranging from 18 to 60 + years) revealed significantly lower lateral ventricle volumes in female than male MS patients specifically between ages 40 and 60, whereas gray matter volumes were lower in male than female patients between ages 18 and 59, but not after age 60 (Jakimovski et al., 2020). The lack of significant sex differences after age 60 are consistent with many other studies discussed in this review, and may be driven by worsening of symptoms in females due to estrogen loss at menopause. Of note, this sample consisted of both RRMS (*n* = 1,554) and PPMS (*n* = 453) patients, as well as 192 individuals who had been diagnosed with a clinically isolated syndrome (CIS)—a single episode of MS‐like CNS inflammatory and demyelinating symptoms that often precedes the development of MS (Miller, Chard, & Ciccarelli, 2012). As CIS is not typically defined as an MS subtype in clinical research, direct comparison of results to other studies should be interpreted with caution. Indeed, exploratory statistical models that included MS subtype as a covariate predictor of imaging outcomes were significant, but post hoc analyses by subtype were not reported (Jakimovski et al., 2020). However, an earlier study conducted by the same group did reveal sex differences in imaging outcomes by MS subtype. Specifically, Antulov et al. (2009) reported significantly smaller volumes in normalized peripheral gray matter and larger volumes in the lateral ventricle of males (*n* = 96) than females (*n* = 403) with RRMS, whereas females with RRMS had significantly lower normalized white matter volumes than RRMS males. Males with progressive MS subtypes (SPMS, PPMS) also had larger third ventricle widths than females, but results did not survive multiple test correction.

Finally, machine learning may have important utility for defining subgroups of MS patients with shared neuroimaging features that may differ by sex. Indeed, a recent machine learning analysis of 8,968 MS patients (RRMS *n* = 2,884; SPMS *n* = 1,837; PPMS *n* = 1,601) revealed three primary MRI phenotypes that were distinguished by the temporal sequence of brain abnormalities along the MS disease course (Eshaghi et al., 2019). (a) *cortex‐first*: most common in RRMS, early atrophy in the occipital, parietal, and frontal cortices and late‐stage reduction in T1/T2 ratio in normal appearing white matter (NAWM; an index of extra‐lesional white matter changes); (b) *NAWM‐first*: most common in PPMS, early reduction in the T1/T2 ratio in NAWM of the cingulum and CC and late stage atrophy of deep gray matter and the frontal, temporal, and parietal cortices; and (c) *lesion‐first*: most common in SPMS, early and extensive T2 lesions and late stage reduction in the T1/T2 ratio in NAWM. A higher proportion of females than males had RRMS (internal validation dataset: 70% females, external validation dataset: 61% females) or SPMS (internal validation dataset: 65% females, external validation dataset: 67% females), whereas PPMS was equally common in both sexes. Interestingly, a higher proportion of females than males had the NAWM‐first neuroimaging phenotype in the external validation dataset only (71% females) (Eshaghi et al., 2019). These comparisons were the only analyses completed to address sex effects, but collectively mark an encouraging step toward data‐driven discovery of neuroimaging phenotypes that have a sex‐specific signature.

Collectively, most of the neuroimaging literature in MS is consistent, generally showing greater imaging abnormalities in males than females, with the exception of white matter indices that tend to be worse in females. Advanced neuroimaging techniques such as quantitative susceptibility mapping (Tolaymat et al., 2020), myelin imaging (Ouellette et al., 2020), and diffusion basis spectrum imaging (Shirani et al., 2019) are emerging techniques in the MS literature that provide enhanced biological information relative to conventional structural and diffusion MRI methods. As there are limited discrepancies to address in the existing MS neuroimaging literature, future studies should capitalize on the strength of these advanced techniques to better define the biological basis for sex differences in MS and its specific subtypes.

### 5.3 Alzheimer's disease

AD is the most common dementia syndrome worldwide, affecting approximately 50 million people around the globe, and 6.08 million people in the United States (Brookmeyer, Abdalla, Kawas, & Corrada, 2018; Prince, 2015). Advanced age is the single greatest risk factor for AD (Alzheimer's Association, 2015; Guerreiro & Bras, 2015), with the majority of AD cases occurring after age 65 (late onset AD). Females have a significantly higher risk for AD than males, representing approximately two thirds of all AD cases (Mielke, Vemuri, & Rocca, 2014). In the United States, the estimated lifetime risk for AD was approximately twice as high in females than males both at ages 45 and 65 (Anon, 2020). Although higher AD risk in females has been largely attributed to increased longevity, many studies suggest this is an oversimplification of numerous confounding factors that may differentially influence male and female AD risk. For example, the e4 allele of the apolipoprotein E (*APOE*) gene is an established genetic risk factor for late onset AD; at least one copy of *APOE*4 is found in over 60% of AD cases (Farrer et al., 1997; Raber, Huang, & Ashford, 2004). Previous work suggests that there may be an interactive effect of *APOE*4 and sex as it relates to age, where females with one copy of *APOE*4 have increased risk of developing AD between ages 65 and 75 compared to males with the same genotype (Neu et al., 2017); we discuss these interactions in relation to imaging variables in the paragraphs below. Other more controversial factors that may contribute to increased AD risk in females include lifetime estrogen exposure, number of pregnancies, and use of HRT (de Lange et al., 2020; Georgakis et al., 2016; Prince et al., 2018).

The neuropathological substrates of AD include abnormal accumulation of amyloid‐beta (Aβ) and neurofibrillary tau tangles (NFT) that results in neurodegeneration and progressive changes in cognition (Bondi, Edmonds, & Salmon, 2017; Jack Jr et al., 2018). Aβ and NFT have long been used as part of the neurodiagnostic system of AD, but disease classification schemes have evolved considerably in the past 30 years. In general, most of the scientific community agrees that AD refers to a continuum of Aβ and NFT accumulation and neurodegeneration that manifests some degree of cognitive impairment, but there are no assumptions about the temporal sequence of these biomarkers or their causal mechanisms (Bondi et al., 2017). In terms of progression, most studies show that AD neuropathology begins in the brainstem and medial temporal lobe (hippocampus, ERC), affecting storage and retrieval of new information early during the disease course (Bondi et al., 2017; Braak & Braak, 1991; Braak & del Tredici, 2015; Braak, Thal, Ghebremedhin, & del Tredici, 2011; Hyman, van Hoesen, Damasio, & Barnes, 1984). Eventually AD pathology spreads beyond the MTL to the neocortex and frontal lobes, affecting other cognitive systems such as language and executive function (Ramirez‐Gomez et al., 2017). Widespread brain atrophy is normative in the late stages of AD which typically corresponds to cognitive impairment in multiple domains and the inability to complete basic or instrumental ADLs (Jack Jr et al., 2018).

Although changes in cognitive function are the focus of most AD clinical research, significant changes in mood, behavior, and somatic symptoms accompany and often precede cognitive decline. Sex differences have been reported in many of these symptoms. Specifically, females with AD exhibit greater insomnia, disability, depression, reclusiveness, emotional instability, delusions, and manic symptoms than males with AD. Females with AD also tend to demonstrate a classical amnestic cognitive signature and faster progression of clinical symptoms and brain atrophy after a suspected diagnosis compared to males with AD (Hebert, Weuve, Scherr, & Evans, 2013; Laws, Irvine, & Gale, 2016; Sinforiani et al., 2010). By contrast, males with AD exhibit greater symptoms of apathy, aggression, agitation and socially inappropriate behaviors (Ferretti et al., 2018). On average, males also exhibit cognitive symptoms at an earlier age, have a shorter disease course, and are more frequently classified by a nonamnestic cognitive phenotype compared to females, despite the presence of AD neuropathology (Liesinger et al., 2018).

Evidence from postmortem studies suggest that males and females exhibit different susceptibilities to AD neuropathology (Braak & Braak, 1991; Serrano‐Pozo, Frosch, Masliah, & Hyman, 2011), particularly in the accumulation of NFT. A neuropathology study in 1,028 deceased individuals (AD, *n* = 736; nondemented controls, *n* = 292; *M*_age_ at death ~82 years) with antemortem clinical assessments showed that females had significantly greater NFT density than males in both the AD and control group after adjusting for age and cognitive symptom duration. Further, a significantly higher proportion of AD females (*n* = 345) reached the highest stage of NFT severity (i.e., Braak Stage VI) compared to AD males (*n* = 391) (Filon et al., 2016). Similarly, Liesinger et al. (2018) revealed increasingly greater NFT burden with advanced age in the hippocampal CA1 and subiculum in females than males with AD at autopsy, with the largest sex differences in the oldest old (ages 90–99). Beyond the hippocampus, males and females had a slightly different pattern of NFT pathology, such that NFT burden was most pronounced in the hippocampus of AD females and in the neocortex of AD males with relative hippocampal sparing. Beyond the hippocampus, AD females had greater NFT burden than AD males in the association cortex after age 60. In early‐onset AD cases (ages 50–59), males had greater NFT burden in the fronto‐parietal cortex than females, but females had around six times the NFT burden in the visual cortex (Liesinger et al., 2018). While these earlier studies provided seminal evidence of sex differences in neuropathology, in vivo examination of AD biomarkers has become the gold standard for assessing AD pathology through CSF analysis of Aβ and tau and/or positron emission tomography (PET) radiotracers that detect amyloid (e.g., Pittsburgh Compound‐B [^11^C‐PiB], ^18^F‐Florbetapir) and tau (^18^F‐AV1451) burden in the brain (Leuzy et al., 2019; Maclin, Wang, & Xiao, 2019; Márquez & Yassa, 2019).

So far, neuroimaging studies of sex differences along the AD continuum generally mirror neuropathological findings. As the hippocampus is one of the earliest targets for AD pathology, several studies have focused on hippocampal morphometry in relation to clinical stages and AD biomarkers (Aβ and tau). In longitudinal work from the AD Neuroimaging Initiative (ADNI) cohort, Koran et al. (2017) examined the impact of sex and AD biomarkers (CSF Aβ_42_, total‐tau) on hippocampal volume and cognitive performance in participants with mixed cognitive status (normal *n* = 348, MCI *n* = 565, AD *n* = 185) at their baseline visit and over an average of 2.5 years (range = 0–9 years, *M*_age_ across all groups at baseline = 72–75 years). Cross‐sectional interactions between sex and AD biomarkers were not significant, but longitudinal analyses revealed significant interactions between sex and AD biomarkers on hippocampal atrophy and cognitive decline on executive and memory tasks after adjusting for age, sex, education, diagnosis, ICV, and scanner strength. Specifically, females exhibited a faster rate of hippocampal volume loss and executive decline than males in the presence of high CSF total tau and low CSF Aβ_42_ (an indicator of high Aβ_42_ in the brain). Memory performance also declined faster in females than males with low CSF Aβ_42_, and additional analyses showed that sex effects were most pronounced in females with lower education and the *APOE*4 genotype (Koran et al., 2017). In more recent work from the National Alzheimer's Coordinating Center (NACC, *N* = 483, *M*_age_ at baseline = 70–78 years), a survival analysis showed that hippocampal volume was a significant predictor of progression to MCI and AD in females, but not males, regardless of AD biomarker status (AD biomarkers were not assessed). Specifically, every 1% increase in hippocampal volume in females was associated with a 46.5% reduction in the rate of progression to AD (*n* = 106), and a 61.4% reduction in the rate of progression to MCI (*n* = 316), over a 10‐year period (Burke et al., 2019).

Although females generally exhibit greater hippocampal volume loss than males, there may be a stronger link between hippocampal volume and AD pathology in males in preclinical stages of AD. For example, recent work using Florbetapir PET to quantify brain amyloid burden in 520 individuals (*M*_age_ = 71.3 ± 6.9; 178 cognitively normal, 342 early MCI) revealed a negative association between high brain amyloid levels (i.e., amyloid‐positivity) and right hippocampal volume in cognitively normal males (*n* = 85, 18.8% amyloid+), but not females (*n* = 93, 36.6% amyloid+), after adjusting for ICV, age, education, *APOE*4 carrier status, and performance on the Montreal Cognitive Assessment (Caldwell et al., 2018). Similar results were observed in the subiculum of the hippocampus in a secondary analysis of hippocampal subfield volumes (parcellated with FreeSurfer‐v.6.0). Specifically, amyloid positivity was negatively associated with right subiculum volume in cognitively normal males, but not females. These sex differences yielded tighter confidence intervals than those in the whole hippocampus, suggesting sex effects in whole hippocampal volume were driven by underlying effects in the subiculum. Neither whole hippocampal volume nor subfield volumes differed by sex in participants with early MCI, and three‐way sex‐by‐diagnosis‐by‐amyloid interactions were not significant in any other subfield. A significant two‐way interaction was reported between sex and diagnosis in the right CA1, but post hoc tests were not reported. While these results suggest possible male vulnerability to amyloid burden than females in the prodromal phases of disease, these findings should be interpreted with caution as there were only 16 males who were both amyloid‐positive and cognitively normal, compared to 34 females who met these criteria (Caldwell et al., 2018). Interestingly, this was not mentioned as a limitation of the study.

Studies of cortical thickness and atrophy in regions beyond the hippocampus also show divergent MRI patterns between males and females along the AD continuum. The most consistent of these patterns is faster atrophy in females than males. In a cross‐sectional vertex‐wide study of cortical thickness in 152 individuals with early stage AD (clinical dementia rating scale = 0.5 or 1), a hierarchical cluster analysis revealed three dominant patterns of cortical atrophy in AD (medial temporal/cingulate, parietal, diffuse), two of which differed by sex (females *n* = 101; males *n* = 51) (Noh et al., 2014). Specifically, a higher proportion of females than males exhibited medial temporal/cingulate dominant atrophy (i.e., *n* = 52, 73.1% females) or diffuse dominant atrophy (i.e., nearly all association cortices affected except occipital and OFC, *n* = 72, 66.7% females), whereas the sex ratio for parietal‐dominant atrophy (i.e., bilateral parietal cortex, precuneus, DLPFC) was approximately equal (*n* = 28, 53.6% females). Individuals with the medial temporal and diffuse patterns were also significantly less educated than those with a parietal pattern, and were more likely to have an *APOE*4 allele, whereas those with the parietal pattern were significantly younger than the other groups and exhibited the worst cognitive performance (Noh et al., 2014). Secondary analyses revealed three additional subtypes within the diffuse atrophy group that differed significantly by sex: medial frontal (*n* = 33, 84.8% females), frontal–parietal (*n* = 31, 58.1% females), and frontal‐temporal (*n* = 8, 25.0% females). The most common pattern in females (medial frontal) was further characterized by greater cortical thickness than the frontal–parietal and frontal‐temporal groups, and poorer performance on tests of verbal and visual recognition (Noh et al., 2014). Although these patterns reflect cross‐sectional estimates of sex differences in “atrophy,” longitudinal work in early‐stage AD patients generally supports the finding of faster cortical thinning in females than males in medial temporal, ACC and frontal areas. Specifically, Lee et al. (2018) showed modestly faster cortical thinning in females (*n* = 22 at baseline, *M*_age_ = 68.4 ± 8.8) than males (*n* = 14 at baseline; *M*_age_ = 73.1 ± 5.6) over a 5‐year period in the DLPFC, medial PFC, STG, ACC, temporal–parietal and occipital cortices after adjusting for age, education, *APOE*4 status, disease duration and onset, and ICV. A sex‐by‐time interaction effect was not significant, but this may be due to the presence of multiple atrophy subtypes, including parietal‐dominant, which did not differ by sex in Noh et al. (2014). Nonsignificant sex interactions may also be a function of small sample size, which was reduced to only 5 males and 12 females at Year 5 due to attrition (Lee et al., 2018). Of note, there were no significant sex differences in cortical thickness at the baseline visit, which is consistent with cortical thickness estimates in a larger study of 193 AD patients (69.4% females) that did not differ significantly by sex (Seo et al., 2011).

Sex differences in neuroimaging markers also exist in earlier stages of the AD continuum, prior to widespread brain atrophy and cognitive impairment. In the ADNI cohort, Hua et al. (2010) compared male and female brain atrophy rates by disease stage (AD = 144, MCI = 338, cognitively normal = 202) over 1 year with tensor‐based morphometry—a warping method used to estimate brain tissue shrinkage and expansion. On average, brain atrophy rates were approximately 1–1.5 times faster in females than males, particularly in the medial temporal lobe among individuals with MCI. In another longitudinal study from ADNI, Skup et al. (2011) revealed significant sex differences in voxelwise atrophy patterns within and across AD diagnostic groups (AD = 197, MCI = 266, cognitively normal = 224) in the caudate, thalamus, MTG, precuneus, caudate, ERC, and insula over a 2–3 year period after adjusting for ICV, education, sex, age, age^2^, and diagnosis. While the results generally show faster decline in females than males, the directionality and magnitude of these sex differences are not easily interpretable based on the many interactions and post hoc tests reported in the manuscript; we summarize these findings as they were originally reported Table S1 in the Supplementary Material.

Sex differences in brain white matter also have been reported along the AD continuum, but less has been done in this area and existing findings are less compelling. For example, Salat, Greve, Pacheco, and Quinn (2009) showed smaller white matter volumes in females (*n* = 54) than males (*n* = 37) with AD (*M*_age_ = 77.6 ± 0.7) in the *pars opercularis*, OFC, STG, MTG, supramarginal, and caudal middle frontal gyri. The authors described the magnitude of these effects as “minor,” but effect sizes were not reported in the manuscript. At the microstructural level, a voxelwise DTI study of sex differences in MCI patients revealed significantly greater white matter disruption (lower FA, higher radial, and mean diffusivity) in females (*n* = 21, *M*_age_ = 66.2 ± 6.5) than males (*n* = 12, *M*_age_ = 71.3 ± 7.0) (O'Dwyer et al., 2012). Finally, several studies have examined sex differences in the role of white matter lesions in AD progression (Kao, Chou, Chen, & Yang, 2019; Lee et al., 2016; Nasrabady, Rizvi, Goldman, & Brickman, 2018) since they are present in the majority of the adult population over age 65 (Paul et al., 2005). White matter lesions are commonly visualized as hyperintense signals (white matter hyperintensities, WMH) on T2‐weighted FLAIR scans because the FLAIR sequence suppresses the CSF signal enabling better lesion detection (Kates, Atkinson, & Brant‐Zawadzki, 1996; Wardlaw, Valdés Hernández, & Muñoz‐Maniega, 2015). WMH have been shown to increase risk for AD disease progression in males, but not females. For example, a longitudinal study of AD risk factors showed that severe WMH volume (>10 mm on MRI) surrounding the ventricles (i.e., periventricular) at baseline predicted a nearly eightfold increased risk of progression from MCI to AD in males over an average of 13.8 months (*n* = 101, hazard ratio [HR] = 7.9, 95% CI [2.4–26.6]) compared to males with mild to moderate periventricular WMH at baseline (Kim et al., 2015). Severe WMH in deep subcortical brain tissue of males, however, were associated with significantly decreased risk for AD progression (HR = 0.08, 95% CI [0.02–0.4]), which was suggested to represent potentially independent mechanisms of periventricular versus deep WMH in AD pathogenesis. There was no significant effect of either periventricular or deep WMH in females (Kim et al., 2015). In an earlier‐mentioned study by the NACC, Burke et al. (2019) showed that WMH risk for AD in males may depend on race and ethnicity, as larger WMH volume predicted faster progression to AD (HR = 4.7, 95% CI [1.1–20.3]) in White males than other race groups over a 10‐year period. Cognitively normal males of *presumably* any race showed a 4.8% increased risk for progression to MCI for every 1% increase in the proportion of WMH to TBV (HR = 1.05, 95% CI [1.01–1.09]); “any race” is presumed because race was not included as a predictor variable in the survival analysis of MCI for reasons that are not stated. A limitation of this study is the predominantly White Non‐Hispanic dataset (~73.3% of the total sample [(*N* = 483]), of which only 14% of the 105 males who progressed to AD were identified as Black/African American. WMH did not influence MCI or AD progression in females regardless of race or ethnicity (Burke et al., 2019).

An important consideration for future work is that inconsistencies in AD diagnostic methods influence purported sex differences in prevalence, onset, and staging of disease, which have downstream effects on neuroimaging research designs. For example, Liesinger et al. (2018) did not find sex differences in AD prevalence when AD was defined strictly by autopsy‐derived neuropathology, but when AD was defined by a combination of clinical and neuroimaging criteria, females were more likely to present with AD compared to males. Vascular risk factors for AD and comorbidity rates between AD and vascular dementia (VaD) are important to consider in the context of sex‐specific prevalence rates for AD. In a recent population‐based study of dementia‐related mortality in Australia, 53% of dementia‐related deaths were documented as “unspecified dementia,” 30% AD dementia, and 12% VaD on death certificates (Buckley, Waller, Masters, & Dobson, 2019). Death rates attributable to AD were higher in females, whereas death rates attributable to VaD were higher in males. However, prior work from the same group revealed that over half of the females with an antemortem dementia diagnosis did not have dementia listed on their death certificate, and nearly 20% of females without an antemortem dementia diagnosis had dementia listed on their death certificate (Waller, Mishra, & Dobson, 2017). These findings highlight reliability issues when using death certificates for retroactive disease classification. Greater prevalence of AD in females may also reflect a survival bias, as AD incidence increases with age and females have longer lifespans than males (Mayeda, 2019). Accordingly, a neuropathology study of AD patients across six decades revealed an overrepresentation of AD females after age 80, whereas males were overrepresented between 50 and 80 years (Liesinger et al., 2018).

### 5.4 Vascular cognitive impairment and dementia

Vascular cognitive impairment (VCI) is a neurocognitive syndrome caused by occlusive or hemorrhagic damage to the cerebrovascular system that eventually impairs ADLs (Paul & Salminen, 2019). Cerebrovascular damage increases with advanced age due to changes in vessel structure (stenosis, stiffness) and accumulation of atherosclerotic plaque (i.e., vascular aging) that reduces blood flow to the brain (Paul & Salminen, 2019). Ischemic stroke is a common consequence of cerebral hypoperfusion that accounts for the vast majority (~87%) of all stroke syndromes (Benjamin et al., 2017). The size and the location of vessel damage and infarction are critical predictors of the resulting cognitive phenotype. Accordingly, approximately one third of patients who suffer from a large vessel stroke meet criteria for VCI within 1 year of the event (Paul & Salminen, 2019). However, VCI is a more common consequence of extensive microvascular dysfunction in small vessels of the brain, which supply blood to subcortical brain structures, particularly in the frontal lobe (Akashi et al., 2017).

Prior studies have noted greater prevalence of subcortical white matter lesions in females than males, and faster progression of small vessel disease. In a large community sample from Rotterdam (*N* = 1,077, ages 60–90 years, 52% females), females had a greater incidence of subcortical white matter lesions than males, particularly in frontal and periventricular regions after accounting for other health confounders (diabetes, hypertension, atherosclerosis); subcortical white matter lesions in females were also more severe compared to males (de Leeuw et al., 2001). A follow‐up study from this group (*N* = 668, ages 60–90, 52% females) revealed a significantly greater likelihood of subcortical white matter lesion progression (OR = 1.8) and incident cerebral infarctions (OR = 1.63) in females than males over a 3‐year period (van Dijk et al., 2008), aligning with earlier work from the Cardiovascular Health Study (*N* = 3,660) showing lower likelihood (OR = 0.74) of white matter lacunes in male participants (Longstreth Jr et al., 1998). A very recent study from the Endovascular Therapy Following Imaging Evaluation for Ischemic Stroke (DEFUSE 3) trial characterized sex differences in cerebrovascular dynamics 6–16 hr after a large vessel occlusion. Here, females (*N* = 90, median age = 72 years) had superior collateral circulation, smaller core lesion volumes, and slower ischemic core lesion growth after large anterior artery occlusions compared to males (*N* = 92, median age = 69 years). However, females did not improve significantly after intra‐arterial medical treatment (while males did improve), and a lower proportion of females (compared to males) achieved functional independence within 90 days (Dula et al., 2020). Divergent findings between males and females emphasize the importance of sex‐specific neurophenotyping, as less severe MRI findings in females may paradoxically translate to significantly worse functional outcomes in females than males.

Less is known about phenotypic sex differences in VCI, and there is essentially no information linking clinical symptoms to specific neuroimaging measures in VCI patients. Of the information we could find, males performed significantly better than females (*N* = 139, *M*_age_ = 69.3 ± 12.3, 43% females) on tests of memory 1‐month after first‐ever stroke, and performed significantly better on tests of psychomotor speed at 1‐month and 6‐months poststroke (Rasquin, Verhey, Lousberg, Winkens, & Lodder, 2002). Males also showed significantly lower likelihood (OR = 0.60) of dysfunction in instrumental ADLs in a large clinical trial of lacunar stroke patients (*N* = 2,820, *M*_age_ = 63.4 ± 10.8, 37% females) (Dhamoon et al., 2015). However, neither of these studies specifically addressed whether participants met criteria for VCI.

While many studies report higher stroke prevalence in males (Barker‐Collo et al., 2015), females exhibit more atypical cardiac symptoms that result in longer delays to seek treatment and a higher rate of misdiagnosis that may contribute to less frequent stroke recovery in females than males (Mayeda, 2019). Atypical cardiac characteristics in females include nonobstructive cardiac plaques and coronary microvascular dysfunction. Further, females are twice as likely as males to receive a diagnosis of heart failure despite preserved ejection fraction. Clinicians who are unaware of these sex differences, or more typically encounter male patients with classical cardiovascular symptoms, may be less likely to diagnose females with VCI or VaD when significant (yet atypical) cardiovascular morbidities are present (Mayeda, 2019).

### 5.5 Frontotemporal dementia

FTD affects the youngest population of the dementia subtypes on average, with a typical age of onset between 45 and 65 years; between 10 and 15% of FTD cases are diagnosed before age 50 (Knopman & Roberts, 2011; Onyike & Diehl‐Schmid, 2013). Approximately 50% of FTD cases show a genetic or familial linkage, making it the second strongest genetically linked condition after autosomal dominant AD. As its name implies, FTD primarily affects the frontal and temporal lobes, with some involvement of the limbic system and striatum (Rohrer, 2012). Neuropathology of FTD is characterized by prominent microvascular changes and severe astrocytic gliosis. These changes occur in the presence of randomly arranged tau filaments (Pick bodies) in approximately 20% of FTD cases (Higgins & Mendez, 2000; Lezak, Howieson, Bigler, & Tranelet, 2004). Over 70% of FTD cases meet diagnostic criteria for both FTD and AD at autopsy (Chare et al., 2014; Lezak et al., 2004; Varma et al., 1999), and typically exhibit focal hippocampal and temporal lobe atrophy. FTD brains that do not resemble the AD phenotype generally show preservation of the acetylcholine system (Huey, Putnam, & Grafman, 2006). Although the cause of nongenetic idiopathic FTD is unknown, it is more common for individuals with a history of traumatic brain injury and thyroid disease (Kalkonde et al., 2012; Rosso et al., 2003).

FTD consists of several subtypes with distinct symptom profiles, and there is some evidence these subtypes differ by sex. The most common subtype is the behavioral variant FTD (bvFTD)—a dysexecutive syndrome with extreme changes in behavior, judgment, and personality (Piguet, Hornberger, Mioshi, & Hodges, 2011). Here behavioral symptoms often precede cognitive changes and diagnosis by many years. Additional characteristics of FTD include lack of insight, stereotypic behaviors (i.e., purposeless involuntary behaviors that occur without conscious control), dysnomia, difficulties with confrontation naming, and poor performance on tests of abstraction and reasoning (Cousins & Grossman, 2017; Ranasinghe et al., 2016). Base rate estimates of bvFTD suggest it is approximately four times more prevalent in males than females, with similar survival rates by sex. In recent work by Ranasinghe et al. (2016), females with bvFTD exhibited more delusions and performed more poorly on tests of executive function and visual free recall than males, whereas males exhibited more apathy, sleep disturbances, and caregiver stress than females. This was the only study we could find that examined sex‐specific clinical characteristics in bvFTD, and although the sample was larger than many studies of sex differences in neurological disease (*N* = 204), the number of males and females was not given (Ranasinghe et al., 2016).

Primary progressive aphasia (PPA) is an umbrella term for two FTD subtypes that interfere with the ability to communicate (e.g., semantic fluent PPA, nonfluent agrammatic PPA), and is approximately twice as common in males than females (Mesulam & Weintraub, 1992; Westbury & Bub, 1997). Although limited work has been done to investigate sex differences in PPA cognitive phenotypes, Rogalski, Rademaker, and Weintraub (2007) showed that females with PPA (*n* = 40) performed more poorly than males with PPA (*n* = 40) on tests of category and letter fluency, and females also exhibited greater longitudinal decline than males. While poorer performance in males might be expected given the higher prevalence rate, a study of three university dementia clinics (*N* = 353) revealed different sex‐specific prevalence rates by PPA subtype, with higher male prevalence in semantic PPA (66.7%) and higher female prevalence in nonfluent PPA (60.9%) (Johnson et al., 2005). In semantic PPA, neuropsychological symptoms are typically characterized by confrontation naming difficulties on the Boston Naming Test, but preserved function on tests of fluency (Marshall et al., 2018). Sex differences in PPA subtype were not examined in Rogalski et al. (2007), but results align with these findings.

We could not find any neuroimaging study that directly examined sex differences in atrophy patterns in FTD or any FTD subtype. In general, MRI studies of FTD show frontal and temporal lobe atrophy in patients versus controls, with relative sparing of posterior brain regions. However, a recent machine learning study of dementia patients in two European memory clinics (*N* = 1,213, 48% female, *M*_age_ = 67 years) showed that only 59% of FTD patients (*N* = 116) demonstrated a distinct frontotemporal atrophy pattern; the rest showed a subcortical atrophy pattern similar to AD (Bruun et al., 2019). Further, across all dementia subtypes (including AD and VaD), the highest classification accuracy was achieved using asymmetric frontotemporal atrophy to distinguish PPA and bvFTD subtypes, and temporal pole volume to distinguish the fluent and nonfluent PPA subtypes (85% AUC for both analyses) (Bruun et al., 2019). These findings may reflect sex differences inherent to the male‐dominant bvFTD subtype, and a female‐dominant nonfluent PPA subtype, but further work is needed to interpret these results.

### 5.6 Dementia with Lewy bodies

Lewy body diseases (LBD) refer to a class of syndromes that involve abnormal aggregation of the alpha‐synuclein protein (i.e., Lewy bodies) in the brainstem, limbic system, basal ganglia, cortical and olfactory network, and ventral forebrain. This pathology can cause motor symptoms, hallucinations, severe functional impairment, and dementia (Mayo & Peavy, 2019). LBD most commonly refers to dementia with Lewy Bodies (DLB), PD, and PD dementia (PDD), as these conditions share many neuropathological, clinical, and prognostic features (Outeiro et al., 2019). The overlap in these conditions is significant, with many reporting underdiagnosis of DLB and overdiagnosis of PD. The differential diagnosis of DLB (vs. PDD) is typically based on the development of dementia within 1 year of motor‐related symptoms (i.e., parkinsonism). A distinguishing feature of LBD vs. AD is the higher prevalence and earlier onset in males than females.

DLB is the third most common type of dementia. It accounts for approximately 4–7.5% of all diagnosed dementia cases (Jones, Vann Jones, & O'Brien, 2014; Kane et al., 2018), but much higher rates of significant DLB pathology have been reported at autopsy (Outeiro et al., 2019). DLB is often misdiagnosed for other neurodegenerative conditions such as AD (McKeith et al., 2017), and true prevalence rates have been estimated as high as 20–30% of all dementia cases (Haider, Spurling, & Sánchez‐Manso, 2020). DLB causes significantly more impairments in ADLs compared to other dementias. The clinical profile of DLB includes cognitive fluctuations, visual hallucinations, parkinsonism, and rapid eye movement sleep behavior disorder, anxiety, depression, and delusions (McKeith et al., 2017). Visual hallucinations are uniquely prevalent in DLB patients (50–60% of cases) and occur early in the disease course (Eversfield & Orton, 2019). Males have approximately four times the incidence rate of DLB compared to females (Savica et al., 2013), though visual hallucinations are reportedly more common in females (Chiu, Teng, Wei, Wang, & Tsai, 2018), particularly at older ages and in the context of more severe neuropsychiatric symptoms. Recent work suggests that DLB is more prevalent in females than males after age 75, which may reflect a survival bias (Mouton et al., 2018). Sex differences have been less frequently studied in DLB than other LBDs.

Structural MRI studies of DLB patients show lower volumes in the frontal and temporal lobes and insular cortex, with relative sparing of the medial temporal lobe and hippocampus in the early stages of disease (Berman & Miller‐Patterson, 2019; Oppedal et al., 2019). Studies using PET and SPECT show hypoperfusion in the occipital cortex and relative sparing of the PCC—a distinguishing feature from AD and potentially PD (Gutman et al., 2018; Ishibashi, Kimura, Sumi, Aso, & Matsubara, 2019). Several studies used DTI to characterize white matter pathology in DLB patients; many show abnormal structural connectivity in visual association areas, but other patterns have been inconsistently defined (Berman & Miller‐Patterson, 2019). However, a literature search for sex differences in neuroimaging characteristics in DLB patients did not yield any results in the past 10 years. In early work from members of our group, males with DLB showed gray matter deficits in frontal dorsal and parietal lobes compared to females with DLB, particularly in the ACC (*N* = 16, *M*_age_ = 76.4 ± 6.7, 50.0% females) (Ballmaier et al., 2004). The lack of literature on neuroimaging sex effects in DLB is concerning, particularly as the hallmark feature—visual hallucinations—is reportedly more common in females than males (Chiu et al., 2018).

## 6 ADDRESSING REMAINING GAPS TO OPTIMIZE RIGOR AND REPRODUCIBILITY

The studies above highlight the progress and gaps in the neuroimaging literature related to sex differences in health and disease. We propose a number of solutions to the pervasive problems in the existing literature, where many studies are underpowered to detect sex differences in disease, despite large differences in risk factors, co‐morbidities, onset, and expression of disease in males and females. In Table 1, we identify several common problems in the neuroimaging literature related to sex differences and offer potential remedies to maximize rigor and reproducibility. We summarize the key messages from Table 1 in the paragraphs below.

**TABLE 1:** Key problems and recommendations for addressing sex‐based knowledge gaps in neuroimaging

Problem	Recommendation
1. Few large studies comparing sex differences in MRI outcomes in major brain diseases	To overcome sample size limitations, leverage big data through accessible sources (ENIGMA, UK Biobank, ADNI, PPMI, NIMH data archive, etc.)
2. “Controlling” for sex as a nuisance covariate	Test sex‐by‐diagnosis interactions and sex‐specific effects when possible. Small samples should conduct sex‐specific analyses when underpowered to examine interactions
3. Inconsistent and/or inappropriate methods to correct for head size	Move beyond trying to “correct” for head size and consider it an explanatory marker Avoid methods that use ratios of regional volume to ICV or TBV to minimize issues with allometric scaling For standard statistical methods, model ICV or TBV as a covariate as long as these variables do not differ across levels of the predictor variable
4. Variability in inclusion criteria, covariate selection, and risk factor modeling	Pre‐register analytic plan and justify covariate use Collect lifetime health histories for all participants Report all lifetime comorbidities, particularly those that are current, to appropriately model and interpret sex effects on neuroimaging Attempt to replicate previous findings by modeling the same set of covariates when possible Model sex‐dependent risk factors (e.g., depression, education, WMH, etc.) using data‐driven methods and/or structural equation models to better interpret sex differences in brain outcomes
5. Lack of dimensional measures assessing the clinical relevance of sex differences in neuroimaging	Map neuroimaging features to clinical phenotypes and examining sex differences in these associations. Clinical phenotyping may be achieved by 1) analyzing NIH RDoC domains across highly comorbid disorders, or 2) using data‐driven clustering methods that include item scores on self‐report measures and neuropsychological tests as input features.
6. Lack of attention to race/ethnicity	Sex‐by‐race or sex‐by‐race‐by‐diagnosis interactions should be tested when possible. Rich assessment of psychosocial and environmental factors that could bias results Examine sex‐by‐race interactions within distinct socioeconomic strata

Abbreviation: WMH, white matter hyperintensities.

### 6.1 Few large studies compare sex differences in MRI outcomes in major brain diseases

An alarmingly small number of neuroimaging studies have assessed structural brain differences between males and females despite known sex differences in disease prevalence and/or clinical features. This literature is particularly sparse for anxiety disorders, DLB, and FTD, with only one neuroimaging study examining sex effects in PaD, one study in DLB, and no studies in GAD or FTD. In many cases there was no clear indication of why such a massive research gap exists more in some diseases than others, but may be due to a combination of factors stemming from a long history of biases affecting recruitment, retention, and basic design decisions. Additionally, small studies still dominate the neuroimaging literature on purported sex effects. The risks of small sample sizes have been well documented over the past 20 years and include (but are not limited to) a high propensity for false negatives, false positives, various forms of publication and reporting bias, and inflated effect sizes due to low statistical power (Button et al., 2013; Ioannidis, 2005a; Ioannidis, 2005b; Ioannidis, 2008; Szucs & Ioannidis, 2017). Indeed, a review of 41 meta‐analyses of brain volume (*n* = 461 unique datasets) revealed an average maximum sample size of 185 per meta‐analysis and median statistical power of only 8% (Button et al., 2013; Ioannidis, 2011). As a result, the number of studies that reported a positive result (a nominally significant effect at *α* < .05) was more than double what would be expected based on formal power calculations if all of the reported effect sizes were accurate, and more than four times the expected number if only half of the reported effect sizes were accurate. Although sex effects were not analyzed, a separate review of MRI studies examining sex effects between 1980 and 2007 reported only one study with a male or female *N* > 100 (Cosgrove, Mazure, & Staley, 2007). While we applaud these pioneer studies for their clairvoyant recognition of the importance of sex research in the era of MRI, their results must be interpreted with caution until they have been replicated in larger datasets.

An obvious solution to the plague of small sample sizes is to analyze more data, but this may be a catch‐22 for investigators without funding to pursue large‐scale recruitment, particularly those in early career stages. Fortunately, large sample sizes can now be achieved without incurring additional costs by participating in consortia such as ENIGMA. ENIGMA was designed to circumvent the high price of large‐scale neuroimaging data collection and capitalize on the massive amounts of archival data that are often untouched after specific research aims are achieved. This global alliance has led the largest‐ever neuroimaging studies of several psychiatric and neurological disorders, and created an unprecedented opportunity to perform well‐powered, large‐scale, investigations of sex differences in neuroimaging across the lifespan. Importantly, ENIGMA is continuously expanding and there is no cost to join (http://enigma.ini.usc.edu/join/). Other large‐scale databases (e.g., UK Biobank, ADNI, PPMI, HCP, etc.) have collected state‐of‐the‐art imaging data and deep phenotyping and are accessible to the research community, but these may cost money and/or require a formal application and review process.

### 6.2 “Controlling” for sex as a nuisance covariate

Covarying for sex has become the gold standard in neuroimaging research, but without good reason. The original and appropriate use for covariates is to improve statistical power to detect associations between an independent variable (*IV*) and the dependent variable (*DV*) after removing *random* variance (i.e., noise) associated with the covariate (*Cov*) (Miller & Chapman, 2001). In the classical use of ANCOVA, *IV* should not share any variance with *Cov*, and the residuals for *IV* and *Cov* should be approximately equal (i.e., *homoscedasticity of residuals*). Further, the relationship between *Cov* and *DV* should be the same between all levels of *IV* (i.e., *homogeneity of regression slopes*) (Lord, 1967). The assumption of statistical independence between *IV* and *Cov* is seemingly not well known by neuroimaging researchers or the greater scientific community at large, as many studies justify the use of a covariate specifically when it differs across levels of the *IV* (van Eersel, Bouwmeester, Verkoeijen, & Polak, 2017). However, when researchers attempt to “control” for the effect of *Cov* that is intrinsically related to the IV, covarying for sex may remove variance associated with preexisting differences in the grouping variable rather than removing *random* variance (i.e., noise) associated with the covariate. This will distort the effect of the IV on brain outcomes, rendering results that are essentially meaningless.

To avoid this outcome, we recommend modeling sex‐by‐diagnosis interactions whenever possible. For smaller cohorts with limited power, sex‐stratified analyses are an acceptable alternative. Low statistical power is the most common reason for nonsignificant sex‐by‐diagnosis interactions, particularly in psychiatric studies where effect sizes tend to be much smaller than those for neurological conditions (Thompson et al., 2020). ENIGMA and other large biobank studies are in a good position to address these issues and test interactions with sufficient statistical power, as well as testing the generalizability and reproducibility of findings across many cohorts and cultures (Palk, Illes, Thompson, & Stein, 2020).

### 6.3 Inconsistent and/or inappropriate methods to correct for head size

Inconsistent or biased adjustments for head size are a major issue for interpreting the results of prior work. Misinterpretation of neuroimaging sex differences is common when the measure of interest is nonlinearly related to brain size (Brun et al., 2009; Jahanshad & Thompson, 2017; Thompson et al., 2000), and we know that certain parts of the brain do not scale linearly with TBV. Many studies of sex differences in the brain have not correctly modeled *allometric scaling*. In Luders, Toga, and Thompson (2014), members of our group examined whether sex differences in brain size versus biological sex accounted for apparent sex differences in the morphology of the CC, the major commissure connecting the two brain hemispheres. These analyses showed that the CC was always larger in men than women, but this difference was strongly determined by sex differences in overall brain size. Comparing CC measures between men and women matched for overall brain size may clarify this, as any observed group difference should indicate pure sex effects. Despite many years of work positing profound sex differences in this structure, hardly any callosal differences remained between brain‐size matched men and women—individual differences in brain size largely accounted for apparent sex differences in corpus callosum anatomy (Luders et al., 2014). Sex differences in cortical gray matter distribution also depend on brain scale (Luders et al., 2005; Luders et al., 2006). Variations in methods for head size or ICV correction are found across the literature, and depending on the method used, incomplete adjustment for ICV effects can yield sex differences in regional measures that simply reflect allometric scaling.

Sex differences in head size may also influence artifact measurements on MRI, such as partial volume effects (PVEs). PVEs may occur when there is CSF contamination of white matter voxels or an unclear boundary between gray and white matter tissue. Both of these issues affect the FA signal (Salminen et al., 2016) and are more likely to arise when there is a mixture of different tissue types in a voxel. As the head is larger, on average, in males than females of the same age, males and females scanned at the same spatial resolution (voxel size) and same field of view will tend to yield scans with a greater number of brain voxels in males than females. This means that more voxels will not have PVEs in males, and smaller structures will also tend to be more susceptible to PVEs in females. As PVEs affect the FA signal, these subtle effects of spatial resolution may mean that FA is lower for people with smaller brains (in this case females relative to males) even if the microstructural properties were exactly the same. This effect of brain scale on diffusion measures is not commonly modeled, but could be modeled using regression of ICV on the derived measures and comparing corrected and uncorrected results.

### 6.4 Inconsistent inclusion criteria, covariate selection, and risk modeling

A large part of the crisis of reproducibility in neuroscience stems from the massive variability in study designs. As we described throughout this review, most studies use different sets of covariates for analyses of sex effects on MRI measures. Indeed, recent work from Hyatt et al. (2020) reported 37 different covariate sets across 50 neuroimaging articles. Additional simulation analyses showed that using different covariate sets significantly altered the observed associations between MRI variables and individual differences in six psychological constructs (e.g., stability, plasticity, internalizing and externalizing psychopathology, executive function, processing speed), sometimes in opposite directions. This variability is a major challenge for interpreting and comparing neuroimaging sex differences from previous studies. We echo the recommendations put forth by Hyatt et al. (2020) to preregister analyses and justify the use of specific covariates prior to conducting the work.

Separately, many neuroimaging and genetic studies assume sources of risk and comorbidities operate in the same way in males and females despite considerable evidence to the contrary, and these assumptions perpetuate inequalities and disparities in health care (Young & Pfaff, 2014). It is important to measure and model risk factors that vary by sex, as restrictive inclusion criteria may model the range of disease expression less fully in one sex versus the other, and may inaccurately represent disease effects on the brain. Even if there were no difference in the way a specific brain disease affects males and females, if there are biases in ascertainment, or sex differences in relevant risk factors or the frequency of co‐morbidities (as we note for many disorders above), then these external factors will influence the observed neuroimaging signature. By contrast, if there is a fundamental sex difference in how a specific disease is expressed in the brain, then current methods to “correct for” sex differences using a covariate may not yield an accurate representation of the disease, unless the studies are also well powered to test and model interactions.

Deeper phenotyping will allow us to measure and model disease‐relevant factors that vary by sex, and to identify factors that might account for any observed sex difference in disease prevalence or expression. Larger multicohort studies in conjunction with deep phenotyping of individual cohorts will help achieve the requisite statistical power to model multilevel interactions between hierarchical feature sets. To merge data from diverse cohorts, statistical harmonization efforts may help, as well as efforts to collect common data elements across funded studies, to facilitate adjustments and comparability of findings.

### 6.5 Lack of dimensional measures for assessing the clinical relevance of sex differences in neuroimaging

True shifts toward personalized medicine will require a new multilevel biotaxy in which clinical symptoms (including physiology, cognition, affect, and behavior) are mapped to neuroimaging indices and other biomarkers according to age, sex, and ethnicity. This precision can be achieved through big data, machine learning, and other bioinformatics strategies that capitalize on the enormous wealth of existing data in the health sciences fields. Adopting these methods is further motivated by increasing numbers of Requests for Applications by the NIH and other funding agencies that emphasize a need for big data initiatives and secondary analyses of existing data. In addition, the NIMH developed the Research Domain Criteria (RDoC) to bridge the gap from research to clinical care by using a dimensional approach to understand biologically based constructs of human behavior (Insel et al., 2010; Sanislow, Ferrante, Pacheco, Rudorfer, & Morris, 2019). These constructs reflect higher‐level knowledge of mental function, acquired through integrative models of neuroimaging, neuropsychological assessment, genomics and other omics‐based indices of molecular biology (e.g., metabolomics, transcriptomics). Currently there are six RDoC domains: (a) negative valence, (b) positive valence, (c) cognition, (d) social processes, (e) arousal/regulatory systems, and (f) sensorimotor systems. Each domain represents a unique system that is characterized by subconstructs of human function that map onto distinct brain circuits, cells, neurotransmitter patterns, and physiological responses. As such, RDoC is an integrative research strategy designed to capture the dimensionality of the human condition, independent of disease states. One of the benefits of the RDoC approach is cross‐disorder synthesis of biological, behavioral, and physiological features, which can then be mapped to neuroimaging. For example, a brief nonscientific survey of the sex‐specific clinical differences reported in this review shows a cross‐disorder pattern of anxiety and depressed mood and hallucinations and delusions in females, and a cross‐disorder pattern of substance misuse and risk‐taking behaviors in males (Marcus et al., 2008). Should these phenotypes be confirmed through actual scientific methods, they can be mapped to MRI outcomes to inform a more complete picture of sex effects on the brain regardless of the overarching disease.

### 6.6 Lack of attention to race/ethnicity

Many studies report health disparities between individuals of different race and ethnic groups (Bell, Thorpe Jr, Bowie, & LaVeist, 2018; Jones et al., 2020; Wilkins, Schindler, & Morris, 2020). Perhaps, the most well‐known examples come from the field of cardiology, where ethnic minorities, particularly those who identify as Black/African American, have a significantly higher risk for cardiovascular disease (e.g., hypertension, diabetes, dyslipidemia) than non‐Hispanic Whites (Howard et al., 2017; Howard et al., 2018; Kurian & Cardarelli, 2007; Lepore et al., 2006; Virani et al., 2020; White et al., 2005). Many other medical disciplines report health disparities by race and ethnicity (Vyas, Eisenstein, & Jones, 2020), including the field of neurology. In one recent study of racial differences in AD biomarkers (Morris et al., 2019), Black/African American participants had lower levels of CSF total tau and phosphorylated tau compared to White participants, with stronger differences in Black/African Americans with the *APOE*4 genotype. Additionally, Black/African American participants with a family history of dementia had significantly lower hippocampal volume than White participants with a family history of dementia (Morris et al., 2019). In another study on nondemented older adults using Florbetapir PET (Gottesman et al., 2016), Blacks had a more than twofold increased risk for elevated amyloid deposition than Whites, which was substantially higher than the risk attributed to female sex (OR ~ 1.7). These two studies represent only a fraction of this emerging literature, but we highlight examples in AD given the higher number of AD studies that also examine sex effects. Indeed, in our review of sex differences in AD neuroimaging markers we described findings from Burke et al. showing that WMH increased risk for AD disease progression, specifically in White males (Burke et al., 2019). Still, most studies have not examined sex‐by‐race or sex‐by‐race‐by‐diagnosis interactions in neuroimaging.

The lack of attention to race and ethnicity in neuroimaging studies is likely due to extremely low representation of minorities in clinical research (Babulal et al., 2019; Canevelli et al., 2019; Heller et al., 2014; Hussain‐Gambles, Atkin, & Leese, 2004). Additionally, analysis of race/ethnicity is complicated by a variety of social and environmental factors (e.g., SES, access to healthcare, exposure to toxins/pollutants, poor nutrition, etc.) that can confound MRI outcomes, if not properly modeled (Crowder & Downey, 2010; Gee & Payne‐Sturges, 2004; LeWinn, Sheridan, Keyes, Hamilton, & McLaughlin, 2017; Nuru‐Jeter et al., 2018; Ward et al., 2019). Unfortunately, stressors that result from systemic racism or discrimination are challenging to measure, but interact with social and environmental health determinants to perpetuate disease inequities in minority populations (Adigbli, 2020).

Fortunately, a number of large‐scale initiatives with neuroimaging data have prioritized the recruitment of race and ethnic minorities, including (but not limited to) the Washington Heights‐Inwood Columbia Aging project (WHICAP; https://www.maelstrom-research.org/mica/individual-study/whicap), Uncovering Neurodegenerative Insights Through Ethnic Diversity (UNITED; https://www.theunitedconsortium.com) (Adams, Evans, & Terzikhan, 2019), the Health and Aging Brain in Latino Elders (HABLE; https://grantome.com/grant/NIH/R01-AG058533-01A1), and the All of Us program (https://allofus.nih.gov/about). These diverse cohorts also exist for studies of brain development (e.g., Adolescent Brain and Cognitive Development [ABCD] Study; https://abcdstudy.org/about/). For researchers with access to these data, we recommend testing two‐way interactions between sex and race/ethnicity on brain outcomes, and three‐way interactions (sex‐by‐race‐by‐diagnosis) for clinical research questions. Interactions should be analyzed in conjunction with a rich set of social and environmental measures to account for potential confounders. Testing sex‐by‐race interactions within and between distinct SES strata may further inform these associations.

## 7 CONCLUSIONS

There are significant sex differences in the mean age of onset of many psychiatric and neurological disorders, in treatment response, symptom severity and disease trajectory, and yet we still lack detailed knowledge of how sex‐specific or sex‐dependent mechanisms contribute to specific neuroimaging phenotypes. This lack of knowledge—even of basic trajectories of brain disease in males versus females—stalls efforts to identify cures for the most common and debilitating mental health and neurological diseases. As we continue to expand international consortia and improve imaging and medical technology, it is imperative to consider the role of sex as a separate etiological factor in psychiatric and degenerative disease, and ensure that this factor is reflected in our neuroimaging designs. Future work using machine learning will also help to discover sex‐dependent interactions with biological, genetic, and environmental factors that influence unique patterns of brain structure and function in males and females across the lifespan.

## CONFLICTS OF INTEREST

P. M. T. received a research grant from Biogen, Inc. (Boston, MA) and served as a consultant for Kairos Venture Capital, Inc., on topics unrelated to this manuscript. The other authors report no conflicts of interest.

## Supporting information


**Table S1** Neuroimaging studies of sex effects in human brain structure

## Data Availability

Data sharing is not applicable to this article as no new data were created or analyzed in this study.

## Endnotes

^1^
An experimental variable was broadly defined as any form of statistical comparison between males and females, including the use of sex as a covariate.

^2^
FD depicts the intra‐axonal restricted compartment of a fiber pathway.

^3^
FC is the approximate fiber bundle area that is perpendicular to fiber length.

^4^
FDC is a summary measure, or the product, of FD and FC (Raffelt et al., 2017).
