# Supplementary material for: Sex is a defining feature of neuroimaging phenotypes in major brain disorders
Source: Hum Brain Mapp. 2021 May 5;43(1):500–42. doi: 10.1002/hbm.25438 (PMC8805690; doi:10.1002/hbm.25438)
Supplement: Supplementary file 1 — Table S1 Neuroimaging studies of sex effects in human brain structure [file HBM-43-500-s001.pdf]

Table S1. Neuroimaging studies of sex effects in human brain structure

Study	Sample	N^	% Female	Age (M ± SD)	Age Range	Design	MRI Modality	Imaging Metric	Main Findings Related to Sex
Normal Populations									
Wierenga et al. 2020	ENIGMA (78 cohorts)	16,683	47.0%	31.1 F*; 31.4 M*	1-90	Cross-Sectional	sMRI	**subcortical VOL**	• Females had smaller VOL than males in all regions
								**SA**	• Females had smaller SA than males in all DK atlas ROIs (n=68)
								**CTh**	• Females had lower CTh than males in 38/68 DK atlas regions (primarily frontal-parietal)
								**subcortical VOL** (between-subject variance)	• Males had greater between-subject variance than females in all subcortical regions
								**SA** (between-subject variance)	• Males had greater between-subject variance than females in all DK atlas ROIs
								**CTh** (between-subject variance)	• Males had greater between-subject variance than females in 41 of 68 DK atlas ROIs
van Eijk et al. 2020	Queensland Twin Imaging Study (QTIM), Human Connectome Project (HCP)	727 QTIM; 960 HCP	63.5% QTIM; 55.5% HCP	23.9 ± 2.5 QTIM; 28.8 ± 3.7 HCP	21-30 QTIM; 22-36 HCP	Cross-Sectional	sMRI	**hippo subfield VOL**	• Females had smaller VOL than males in parasub, fimbria, hippo fissure, presub<br>• Males had smaller VOL than females in hippo tail<br>• No sex effects in CA2/3, CA4, HATA, or GCDG (adjusting for total hippo VOL or ICV)
Ching et al. 2020	UK Biobank (UKB)	26,440 full sample; 9,414 N with *APOE* genotyping	52.1% full sample; 52.0% N with *APOE* genotyping	63.5 ± 7.5 full sample; 62.6 ± 7.5 N with *APOE* genotyping	44-81	Cross-Sectional	sMRI	**subcortical VOL** (change with age)	• **All ages:** Females had less VOL change with age than males in all regions<br>• **Over age 60:** Females had less subcortical VOL change in lat ventricles, caudate with age than males<br>• 3-way interaction tests between sex, age, *APOE*4 status not significantly associated with VOL change
Dima et al. 2020	ENIGMA (88 cohorts)	11,550	53.8%	32.9 ± 18.3*	6-90	Cross-Sectional	sMRI	**ICV-adjusted subcortical VOL***	• Correlations between age and subcortical VOL did not differ by sex

Table S1. Neuroimaging studies of sex effects in human brain structure

Frangou et al. 2020	ENIGMA (83 cohorts)	17,075	52.0%	31.0 ± 18.2	3-90	Cross-Sectional	sMRI	**CTh**	• **Lifespan**: No sex differences in total CTh<br>• **Ages 3-29:** Males had stronger negative associations between CTh and age than females in: BL cuneus, BL lat occipital, BL lingual, BL SPG, BL postcentral, BL paracentral, BL precuneus, BL PCAL<br>• **Ages 30-59:** Males had stronger negative association between age and CTh than females in: BL pars orbitalis, BL pars triangularis, L isthmus cingulate, L pars opercularis, L precuneus, L RMFG, L SMG, R fusiform, R insula, R lat OFC, R rostral ACC, R ITG, R IPL, R lat occipital, R SFG, R SMG
Yang et al. 2020	HCP, Chinese HCP	250 HCP; 250 CHCP	50% HCP; 48.4% CHCP	22.8 ± 3.2 HCP; 21.5 ± 2.4 CHCP	22-35 HCP; 19-37 CHCP	Cross-Sectional	sMRI	**CTh**	• **Chinese HCP:** Females had lower CTh than males<br>• **Caucasian HCP:** Males had lower CTh than females in R caudal ACC adjusting for age, ICV<br>• **Caucasian HCP:** Females had lower CTh than males adjusting for age, ICV in: R insula, L lat OFC, R isthmus cingulate
								**Sulcal depth**	• **In Chinese HCP:** Females had larger sulcal depth than males adjusting for age, ICV in: BL superior temporal sulci, R insula, R precuneus<br>**In Chinese HCP**: Males had larger sulcal depth than females adjusting for age, ICV in: BL ITG, R IPL, R medial OFC<br>• **In Caucasian HCP:** Females had larger sulcal depth than males adjusting for age, ICV in: L parietal lobe, L STG, L rostral MFG, R SFG<br>• **In Caucasian HCP:** Males had larger sulcal depth than females adjusting for age, ICV in: R ITG, R precuneus, R pars triangularis
Nobis et al. 2019	UKB	19,793	52.9%	63.0 ± 7.5	45-80	Cross-Sectional	sMRI	**hippo VOL**	• No sex effects on hippo VOL
								**GM VOL**	• Males had smaller total GM VOL than females (correcting for head size)
								**cortical VOL**	• Females had smaller VOL than males in PHG, temporal pole
								**hippo VOL** (change with age)	• **Ages 60-65:** Females had faster VOL loss than males

Table S1. Neuroimaging studies of sex effects in human brain structure

Ritchie et al. 2018	UKB	5,216	52.7%	61.7 ± 7.5	44-77	Cross-Sectional	sMRI	**TBV**	• Females had smaller TBV than males
								**CTh**	• Males had lower CTh than females in most regions (adjusting for TBV, age, ethnicity)<br>• Females had lower CTh than males in R insula (adjusting for TBV, age, ethnicity)<br>• Females had lower CTh than males in medial OFC, rostral ACC (adjusting for TBV, age, ethnicity)<br>• Males had lower CTh than females in 24/68 ROIs (adjusting for total mean CTh, age, ethnicity)<br>• Females had lower CTh than males in 25/68 ROIs (adjusting for total mean CTh, age, ethnicity)
								**subcortical VOL**	• Males had smaller VOL than females in nucleus accumbens (adjusting for TBV, age, ethnicity)<br>• Females had smaller VOL than males in putamen, amygdala, pallidum (adjusting for TBV, age, ethnicity)
								**cortical VOL**	• Females had smaller cortical VOL than males in 11/68 ROIs (DK atlas) (adjusting for TBV, age, ethnicity)<br>• Males had smaller cortical VOL than females in 13/68 ROIs (adjusting for TBV, age, ethnicity)
								**SA**	• Females had smaller cortical SA than males in 18/68 ROIs (adjusting for total SA, age, ethnicity)<br>• Males had smaller cortical SA than females in 9/68 ROIs (adjusting for total SA, age, ethnicity)
							dMRI/ NODDI	**FA**	• Males had lower FA than females in L ILF, L PTR<br>• Females had lower FA than males in R ARC, BL CST and BL STR
								**OD**	• Males had higher OD than females in all WM tracts
Kong et al. 2018	ENIGMA (99 cohorts)	17,141	51.3%*	Median age available by cohort in original publication	3-90	Cross-Sectional	sMRI	**CTh asymmetry**	• Males had more leftward CTh asymmetry than females in PHG and ERC
								**SA asymmetry**	• Males had more rightward global SA asymmetry than females<br>• Males had more rightward SA asymmetry than females in SFG, pars orbitalis, STG, temporal pole, PHG, fusiform, IPL, SMG, and ACC

Table S1. Neuroimaging studies of sex effects in human brain structure

Farokhian et al. 2017	Information Extraction from Images Dataset (IXI Dataset)	142 Young; 135 Old	50.0% Young; 51.1% Old	27.9 ± 3.8 Young M; 27.1 ± 3.6 Young F; 68.4 ± 5.8 Old M; 68.4 ± 5.8 Old F	20-34 Young; 60-86 Old	Cross-Sectional	sMRI/ VBM	**GM VOL**	• Males had smaller total GM VOL than females<br>• Females had a steeper negative association between age and GM VOL than males
Guadalupe et al. 2017	ENIGMA (52 cohorts)	15,847	52.5%	Mean age available by cohort in original publication	~5-85	Cross-Sectional	sMRI	**subcortical VOL asymmetry**	• Males had greater rightward asymmetry than females in putamen<br>• Females had greater leftward asymmetry than males in globus pallidus
Malykhin et al. 2017	University of Alberta	129	54.3%	47.6 ± 18.9	18-85	Cross-Sectional	sMRI	**hippo VOL**	• Males had smaller VOL than females in head (DG), body (CA1-3, subiculum, DG), tail (all subfields), and DG normalizing hippo VOL by ICV (raw hippo VOL / ICV of same subject x sample averaged ICV) and removing effects of age
Dennis et al. 2017	QTIM	667	62.0%	22.7 ± 2.8	18-30	Cross-Sectional	dMRI	**FA**	• Males had lower FA than females in R frontal callosal fibers and R IFOF<br>• Females had a more positive association between FA and age than males in R frontal callosal fibers and R IFOF
Tan et al. 2016	Meta-Analysis; (29 studies that corrected for ICV or TBV)	2,183	52.8%	36.9 ± 25.1*	Birth-79 (mean age range across studies)	Cross-Sectional	sMRI	**hippo VOL**	• No sex differences in hippo VOL (adjusting for TBV or ICV)
Kochunov et al. 2015	HCP	481	60.0%	29.1 ± 3.5	22-36	Cross-Sectional	dMRI / TBSS	**FA**	• Males had ~2% lower global FA than females<br>• Males had lower tractwise FA than females in most WM tracts; strongest effects in internal capsule and fornix
Post-traumatic Stress Disorder (PTSD)									
Wang et al. 2020	ENIGMA (28 cohorts)	1,379 PTSD; 2,192 Control	41.5% PTSD; 42.1% Control	36.0 ± 14.1 PTSD; 34.3 ± 15.5 Control	6-85	Cross-Sectional	sMRI	**cortical VOL**	• No significant sex-by-PTSD interactions on cortical VOL
Dennis et al. 2019	ENIGMA (28 cohorts)	1,426 PTSD; 1,621 Control	28.7%	39.6	18-83	Cross-Sectional	dMRI	**FA**	• No significant sex-by-PTSD interactions on regional FA and total mean FA
Logue et al. 2018	ENIGMA (16 cohorts)	794 PTSD; 1,074 Control	39.6%	36.4*	8-83	Cross-Sectional	sMRI	**subcortical VOL**	• No significant sex-by-PTSD interaction on hippo VOL<br>• **In females only:** smaller hippo VOL in PTSD than controls<br>• **In males only:** No case-control differences
Generalized Anxiety Disorders (GAD)									
No studies evaluating neuroimaging sex effects were found.									
Panic Disorder									

Table S1. Neuroimaging studies of sex effects in human brain structure

Asami et al. 2009	Yokohama City University Hospital	24 PaD;<br>24 Control	62.5% PaD;<br>62.5% Control	33.4 ± 9.1 PaD M;<br>39.2 ± 10.5 PaD F;<br>33.2 ± 5.2 Control M;<br>39.3 ± 10.8 Control F	19-57	Cross-Sectional	sMRI/ VBM	**cortical VOL**	• **In PaD:** smaller VOL in males than females in BL insula, R amygdala, and L OTG<br>• **In PaD:** smaller VOL in females than males in R STG<br>• **In females only:** smaller VOL in PaD than controls in BL DLPFC, ventrolateral PFC, thalamus, parietal cortex, and R cerebellar vermis<br>• **In males only**: No case-control differences
Obsessive Compulsive Disorder (OCD)									
Boedhoe et al. 2018	ENIGMA (26 cohorts)	1,498 OCD;<br>1,436 Control	50.5% OCD;<br>49.7% Control	31.4 ± 9.9 OCD;<br>30.7 ± 10 Control	NA	Cross-Sectional	sMRI	**CTh**	• No significant sex-by-OCD interaction on CTh in 68 DK atlas ROIs
								**SA**	• No significant sex-by-OCD interaction on SA in 68 DK atlas ROIs
Piras et al. 2019	ENIGMA (19 cohorts)	700 OCD*;<br>645 Control*	42.1% OCD*;<br>41.4% Control*	31.4 ± 9.9 OCD;<br>30.7 ± 10 Control	NA	Cross-Sectional	dMRI/ TBSS	**FA, MD, RD, AxD**	• No significant sex-by-OCD interactions on regional FA, AxD, MD, or RD
Boedhoe et al. 2017	ENIGMA (25 cohorts)	1,495 OCD;<br>1,472 Control	49.9% OCD;<br>52.9% Control	32.0 ± 9.8 OCD;<br>30.6 ± 9.8 Control	NA	Cross-Sectional	sMRI	**subcortical VOL**	• No significant sex-by-OCD interactions on any subcortical VOL
Hawco et al. 2017	Centre for Addiction and Mental Health Toronto, Canada	38 OCD;<br>45 Control	55.3% OCD;<br>48.9% Control	34.6 ± 11.3 OCD;<br>33.2 ± 10.3 Control	18-60	Cross-Sectional	dMRI	**FA**	• **In OCD patients:** Females had lower whole brain FA than males
Major Depressive Disorder (MDD)									
van Velzen et al. 2020	ENIGMA (16 cohorts)	1,305 MDD;<br>1,602 Control	60.2% MDD*;<br>63.1% Control*	41.1 MDD*;<br>37.0 Control*	21-88	Cross-Sectional	dMRI	**FA, MD, RD, AxD**	• No significant sex-by-MDD interactions on regional FA, AD, RD, or MD
Tozzi et al. 2020	ENIGMA (12 cohorts)	1,284 MDD;<br>2,588 Control	63.3% MDD;<br>50.3% Control	40.9 ± 14.6 MDD;<br>43.3 ± 15.9 Control	13-89	Cross-Sectional	sMRI	**CTh**	• **In females:** greater childhood trauma severity associated with smaller total CTh<br>• **In males:** no effect of childhood trauma severity on total CTh<br>• **In males:** greater childhood trauma severity associated with greater CTh in rostral ACC<br>• **In females:** no effect of childhood trauma severity on regional CTh
								**SA**	• **In MDD males:** males with childhood abuse/neglect had lower SA than males without childhood trauma in caudal ACC

Table S1. Neuroimaging studies of sex effects in human brain structure

Ancelin et al. 2019	French ESPRIT Study	162 Lifetime MDD; 448 No lifetime MDD	53.5% whole sample	70.7 (median) whole sample	67-74	Cross-Sectional	sMRI	**subcortical VOL**	• **In males:** smaller VOL in lifetime MDD than no lifetime MDD in amygdala and caudate<br>• **In females:** no case-control differences in regional VOL
								**cortical VOL**	• **In females:** larger VOL in lifetime MDD females than no lifetime MDD in rostral ACC<br>• **In males:** no case-control differences in regional VOL
Lyon et al. 2019	International Study to Predict Optimsed Treatment in Depression (iSPOT-D)	221 MDD; 67 Control	52% MDD; 51 Control	33.6 ± 11.7 MDD; 30.3 ± 12.8 Control	18-65	Cross-Sectional	dMRI / Fixel-based analysis	**fiber density cross-section (FDC)**	• **In females:** MDD had lower FDC than controls in genu of CC<br>• **Males:** MDD had lower FDC than controls in R ALIC
								**fiber cross-section (FC)**	• **Females:** lower FC in MDD than controls in CC, R ALIC, R tapetum, R ILF<br>• **Males:** lower FC in MDD than controls
Frodl et al. 2017	ENIGMA (9 cohorts)	958 MDD; 2,078 Control	64.1% MDD; 47.8% Control	42.4 ± 14.3 MDD; 46.3 ± 15.2 Control	NA	Cross-Sectional	sMRI	**subcortical VOL**	• **In females:** greater childhood trauma severity associated with smaller VOL in BL caudate<br>• **In males:** childhood trauma severity not associated with subcortical VOL
Schmaal et al. 2017	ENIGMA (20 cohorts)	1,911 MDD*; 7,663 Control*	61.7% MDD*; 52.6% Control*	40.5 MDD*; 38.9 Control*	NA	Cross-Sectional	sMRI	**CTh**	• No significant sex-by-MDD interactions
								**SA**	• No significant sex-by-MDD interactions
Schmaal et al. 2016	ENIGMA (15 cohorts)	1,728 MDD; 7,199 Control	52.8% MDD*; 62.6% Control*	43.3 MDD*; 56.0 Control*	NA	Cross-Sectional	sMRI	**subcortical VOL**	• No significant sex-by-MDD interactions
Kong et al. 2013	China Medical University	29 MDD; 33 Control	55.2% MDD; 51.5% Control	29.5 ± 6.8 MDD; 29.9 ± 8.3 Control	18-45	Cross-Sectional	sMRI/ VBM	**GM density**	• **In males:** lower GM density in MDD than controls in BL caudate to L ventral striatum<br>• **In females:** lower GM density in MDD than controls in BL amygdala and hippo
								**subcortical VOL**	• **In males:** smaller VOL in MDD than controls in BL caudate<br>• **In females:** no case-control differences in subcortical VOL
Bipolar Disorder (BD)									
Hibar et al. 2018	ENIGMA (28 cohorts)	2,447 BD; 4,056 Control	59.7% BD*; 54.9% Control*	38.4 BD; 35.6 Control	~17-65	Cross-Sectional	sMRI	**CTh**	• **BD aged < 25 years:** larger CTh in females than males in insula, temporal pole, pars tri, SFG<br>• **BD aged > 25 years:** No sex differences in regional CTh
								**VOL**	• BD males had smaller thalamus VOL than BD females
								**SA**	• No significant sex-by-BD interactions

Table S1. Neuroimaging studies of sex effects in human brain structure

Hibar et al. 2016	ENIGMA (20 cohorts)	1,710 BD; 2,594 Control	59.1% BD; 55.4% Control	40.1 BD*; 36.5 Control*	~17-70	Cross-Sectional	sMRI	**subcortical VOL**	• **In BD:** males had smaller thalamus VOL than females
Schizophrenia (SCZ)									
Du Plessis et al. 2020	Tygerberg/Stikland Hospitals	79 SCZ; 82 Control	26% SCZ; 43% Control	23.0 ± 7.0 SCZ; 23.0 ± 7.0 Control	16-45	Cross-Sectional	sMRI	**hippo subfield VOL**	• **SCZ females** had larger hippo fissure VOL than other subgroups<br>• **In SCZ females:** greater childhood trauma severity associated with larger hippo fissure VOL<br>• **In SCZ males**: no association between childhood trauma and regional VOL
Honnorat et al. 2019	Ludwig-Maximilian University	157 SCZ; 169 Control	24.8% SCZ; 31.4% Control	31.0 ± 9.1 SCZ; 31.6 ± 9.3 Control	NA	Cross-Sectional	sMRI / VBM	**GM VOL**	• Machine learning revealed 3 latent clusters that differed significantly by sex Cluster 1: less GM in thalamus, ACC, and STG and CSF expansion in temporal, thalamic, and perisylvian areas, altered GM/WM contrast, longest disease duration Cluster 2: CSF expansion predominantly in frontal regions, lower VOL associated with positive SCZ symptoms and marginally earlier disease onset Cluster 3: mildest CSF VOL expansion, lower TBV and lower education Clusters 1 and 2: >80% male Cluster 3: approx balanced sex distribution (56% male)
van Erp et al. 2018	ENIGMA (39 cohorts)	4,474 SCZ; 5,098 Control	34.0% SCZ; 47.0% Control	32.3 SCZ; 32.8 Control	10-87	Cross-Sectional	sMRI	**CTh**	• No significant sex-by-SCZ interactions
								**SA**	• No significant sex-by-SCZ interactions
Kelly et al. 2018	ENIGMA (29 cohorts)	1,963 SCZ; 2,386 Control	33% SCZ; 46.6% Control	36.2 SCZ; 36.1 Control	18-86	Cross- Sectional	dMRI	**FA, MD, RD, AxD**	• No significant sex-by-SCZ interactions on FA, MD, RD, AxD
								**FA**	• **In males separately:** lower FA in (14/25) tracts in SCZ than controls<br>• **In females separately:** lower FA (20/25 WM tracts) in SCZ than controls with female-specific significant effects in SCR, UNC, IFOF, CHC, and internal capsule
Womer et al. 2016	First Affiliated Hospital of China Medical University	50 SCZ; 54 Control	52.0% SCZ; 55.6% Control	30.9 ± 10.4 SCZ; 32.7 ± 10.7 Control	NA	Cross-Sectional	sMRI	**cerebellar vermis VOL**	• **In males:** smaller cerebellar vermis VOL in SCZ than controls (adjusting for age and TBV)<br>• **In females:** no case-control differences in cerebellar vermis VOL (adjusting for age and TBV)

Table S1. Neuroimaging studies of sex effects in human brain structure

Takayanagi et al. 2011	Tokyo Metropolitan Matsuzawa Hospital	52 SCZ;<br>40 Control	44.2% SCZ;<br>45.0% Control	27.9 ± 6.4 SCZ M*;<br>28.2 ± 7.2 SCZ F*;<br>30.3 + 5.8 Control M*;<br>28.0 ± 4.3 Control F*	< 45	Cross-Sectional	sMRI	**cortical VOL**	• **In SCZ:** smaller BL amygdala VOL in females than males
Exner et al. 2008	University of Marburg	21 SCZ;<br>21 Control	33.3% SCZ;<br>38.1% Control	31.0 ± 11.0 SCZ M;<br>32.0 ± 10.0 SCZ F;<br>29.0 ± 10.0 Control M;<br>32.0 ± 9.0 Control F	NA	Cross-Sectional	sMRI	**hippo VOL**	• **In males:** smaller BL hippo VOL in SCZ than controls<br>• **In females**: no case-control differences
Duggal et al. 2005	University of Pittsburgh Medical Center	30 SCZ;<br>30 Control	50% SCZ;<br>50% Control	31.4 ± 5.5 SCZ M;<br>25.9 ± 8.7 SCZ F;<br>33.5 ± 7 Control M;<br>26.5 ± 11.4 Control F	NA	Cross-Sectional	sMRI	**insula VOL**	• **In females:** smaller VOL in SCZ than controls in R insula<br>• **In males:** no case-control differences in insula VOL
Niu et al. 2004	Toyama Medical and Pharmaceutical University Hospital	40 SCZ;<br>40 Control	50% SCZ;<br>50% Control	26.4 ± 5.1 SCZ M;<br>25.9 ± 5.2 SCZ F;<br>25.5 ± 5.6 Control M;<br>24.8 ± 6.2 Control F	NA	Cross-Sectional	sMRI	**amygdala VOL**	• **In males:** smaller amygdala VOL in SCZ than controls<br>• **In females:** no case-control differences in amygdala VOL<br>• **SCZ females** had smaller R amygdala VOL than SCZ males
Goldstein et al. 2002	Massachusetts General Hospital	40 SCZ;<br>48 Control	32.5% SCZ;<br>43.8 Control	46.6 ± 11.4 SCZ M;<br>41.4 ± 7.0 SCZ F;<br>41.6 ± 11.6 Control M;<br>39.9 ± 9.9 Control F	23-68	Cross-Sectional	sMRI	**cortical VOL**	• **In males:** smaller VOL in SCZ than controls in frontomedial (BA 11, 12) and middle frontal cortices, (BA6, 8, 9, 46) paracingulate gyrus (BA32), insula (BA13, 14, 15, 16), Heschl's gyrus (BA 41), and Broca's area<br>• **In females:** smaller VOL in **SCZ** than **controls** in OFC, basal forebrain, ACC, posterior SMG
								**VOL asymmetry**	• **In males:** less asymmetry in SCZ than controls in R vs L planum temporale VOL<br>• **In females:** greater rightward planum temporale VOL asymmetry in SCZ than controls
								**subcortical VOL**	• No significant sex-by-SCZ interactions
								**ventricle VOL**	• No significant sex-by-SCZ interactions
Takahashi et al. 2002	Toyama Medical and Pharmaceutical University Hospital	40 SCZ;<br>40 Control	50% SCZ;<br>50% Control	26.1 ± 5.0 SCZ;<br>25.1 ± 5.8 Control	15-38	Cross- Sectional	sMRI	**cortical VOL**	• **In females:** smaller VOL in SCZ than controls in R ACC<br>• **In males:** no case-control differences in GM VOL
								**cortical VOL asymmetry**	• **In females:** greater rightward asymmetry in ACC VOL in controls than SCZ<br>• **In males:** no case-control differences in asymmetry

Table S1. Neuroimaging studies of sex effects in human brain structure

Narr et al. 2001	National Health Services (NHS) Hospitals	25 SCZ; 28 Control	40% SCZ; 46.4% Control	32.4 ± 7.9 SCZ M; 39.9 ± 10.2 SCZ F; 33.0 ± 10.1 Control M; 35.2 ± 9.0 Control F	NA	Cross-Sectional	sMRI	**ventricle VOL**	• **In males:** larger ventricle VOL in SCZ than controls<br>• **In females:** no case-control differences
								**subcortical VOL asymmetry**	• **In males:** greater rightward asymmetry in hippo VOL in SCZ than controls<br>• **In females:** no case-control differences
Bryant et al. 1999	Maryland Psychiatric Research Center	59 SCZ; 37 Control	40% SCZ; 48.6 Control	32.66 ± 5.5 SCZ M; 37.3 ± 5.1 SCZ F; 34.3 ± 6.6 Control M; 33.3 ± 8.0 Control F	21-45	Cross- Sectional	sMRI	**GM VOL**	• **In males:** smaller VOL in L temp lobe in SCZ than controls<br>• **In females:** no case-control differences in regional VOL<br>• No significant sex-by-SCZ interactions on *a-priori* selected regions: STG, amygdala/hippo complex, prefrontal GM, prefrontal WM, and caudate
Substance Use Disorder (SUD)									
Grace et al. 2020 (in press)	ENIGMA (10 cohorts)	643 Alcohol dependent; 323 Control	50.2% Alcohol dependent; 30.3% Control	34.3 ± 10.4 Alcohol dependent M; 32.7 ± 10.5 Alcohol dependent F; 30.3 ± 10.5 Control M; 39.5 ± 9.8 Control F	NA	Cross-Sectional	sMRI	**amygdala subfield VOL**	• **In males:** alchohol-dependents had smaller VOL than controls in total amygdala, basolateral nucleus (adjusting for age, education, ICV, and tobacco use)<br>• **In females:** no case-control differences in amgdala subfield VOL<br>• **In Alcohol-dependents:** females had smaller VOL than males in accessory basal, anterior and cortico-amygdaloid nuclei
								**hippo subfield VOL**	• No significant sex-by-alcohol dependence interactions on hippo subfields (CA1, CA3, subiculum, DG)<br>• **In Alcohol-dependents:** males had smaller VOL than females in BL HATA and R fimbria (adjusting for age, education, ICV, and tobacco use)
Rabin et al. 2020	ENIGMA (7 cohorts)	210 Cocaine-dependent; 210 Control	33.3% Cocaine-dependent; 33.3% Control	37.8 ± 6.7 Cocaine-dependent M; 39.6 ± 7.6 Cocaine-dependent F; 37.0 ± 8.5 Control M; 37.2 ± 9.8 Control F	NA	Cross-Sectional	sMRI / VBM	**cortical VOL**	• **In females:** lower VOL in cocaine-dependents than controls in L anterior insula, L lingual gyrus<br>• **In males:** no case-control differences in cortical VOL
								**hippo VOL**	• **In cocaine-dependent males:** longer cocaine use duration associated with smaller R hippo VOL<br>• **In cocaine-dependent females:** no association between cocaine use duration and hippo VOL
Sawyer et al. 2020	Massachusetts General Hospital	67 Abstinent alcoholics; 64 Control	46.2% Abstinent alcoholics; 48.4% Control	51.1 ± 10.8 Abstinent alcoholics M; 54.5 ± 12 Abstinent alcoholics F; 50.5 ± 12.3 Control M; 53.6 ± 15.8 Control F	27-82	Cross- Sectional	sMRI	**hippo subfield VOL**	• No significant sex-by-diagnosis interactions on hippo subfield VOL<br>• **In females:** longer length of sobriety associated with smaller CA1 VOL<br>• **In males:** longer length of sobriety associated with larger CA1 VOL

Table S1. Neuroimaging studies of sex effects in human brain structure

Rossetti et al. 2019	ENIGMA (4 cohorts)	129 Cannabis users; 114 Control	29.5% Cannabis users; 28.9% Control	27.5 ± 10.1 Cannabis users; 26.2 ± 9.1 Control	NA	Cross-Sectional	sMRI	**GM VOL**	• **In males:** higher monthly cannabis use associated with lower cerebellar GM VOL (adjusting for ICV, age, IQ, montly standard drinks, and montly cigarettes)
								**WM VOL**	• **In females:** Cannabis dependents had smaller WM VOL in cerebellum and a thinner OFC compared to non-addicted cannabis users and controls (adjusting for ICV, age, IQ, montly standard drinks, and monthly cigarettes)<br>• **In males:** cannabis use status not associated with WM VOL (adjusting for ICV, age, IQ, monthly standard drinks, and monthly cigarettes)<br>• **In males:** earlier cannabis use onset associated with lower cerebellar WM VOL (adjusting for ICV, age, IQ, montly standard drinks, and monthly cigarettes)
								**CTh**	• **In males:** cannabis use status not associated with CTh (adjusting for ICV, age, IQ, monthly standard drinks, and monthly cigarettes)
Sawyer et al. 2018	Boston University School of Medicine/ Massachusetts General Hospital	49 Alcoholics; 41 Control	53.1% Alcoholics; 53.7 Control	54.0 ± 11.4 Alcoholic M; 51.6 ± 12.0 Alcoholic F; 49.9 ± 13.4 Control M; 56.7 ± 14.0 Control F	23-76	Cross-Sectional	dMRI / TBSS	**FA**	• **In males:** lower FA in alcoholics than controls in CC, SLF, ARC and external capsule<br>• **In females:** higher FA alcoholics than controls in CC, SLF, ARC and external capsule<br>• **Male alcoholics** had lower FA in one TBSS cluster in superior to posterior horn of L lateral ventricle (including posterior thalamic radiation)<br>• **In females only:** no case-control differences in FA clusters<br>• **In male alcoholics:** longer length of sobriety associated with higher FA<br>• **In female alcoholics:** no significant association between length of sobriety and FA
Monnig et al. 2015	University of New Mexico	114 Treatment-seeking heavy drinkers; 189 Treatment-naive heavy drinkers	33.0% Treatment-seeking; 29.0% Treatment-naive	30.9 ± 9.1	21-56	Cross-Sectional	dMRI	**FA**	• In **females:** higher number of drinks per day associated with lower FA in a latent “WM factor” consisting of average FA across CC, fornix, external capsule, SLF, and cingulum<br>• In **males:** higher number of drinks per day not associated with lower FA in a latent "WM factor" of average FA in CC, fornix, external capsule, SLF, and cingulum

Table S1. Neuroimaging studies of sex effects in human brain structure

Rando et al. 2013	Connecticut Mental Health Center	36 Cocaine users; 50 Control	50.0% Cocaine users; 44.0% Control	38.2 ± 5.4 Cocaine users M; 36.7 ± 5.6 Cocaine users F; 30.9 ± 9.7 Control M; 31.5 ± 8.3 Control F	18-50	Cross-Sectional	sMRI / VBM	**VOL**	• All **females** had smaller VOL than all males in BL cerebellum and PCC<br>• All **females** had larger VOL than all males in L STG and L insular, orbital, IFG, MFG, and SFG<br>• **In females:** larger BL cerebellum VOL in cocaine users than controls
								**GM VBM**	• **In females:** smaller GM VOL in cocaine users than controls in a R hemisphere cluster including inferior/middle occipital gyri, MTG, ITG, IPG, angular and SMG gyri, and L hemisphere cluster including insula, STG, hippo, and anterior IFG<br>• **In males:** smaller VOL in cocaine users than controls in a VBM cluster including BL mid-cingulate cortex, SMA, SFG, MFG, paracentral lobule, precentral and postcentral gyri
Parkinson's disease (PD)									
Laansma et al. 2020 (preprint)	ENIGMA (43 cohorts)	2,367 PD; 1,183 Control	36.0% PD; 46.0% Control	63.4 PD; 59.4 Control	NA	Cross-Sectional	sMRI	**CTh**	• No significant sex-by-PD interactions
								**SA**	• No significant sex-by-PD interactions
								**subcortical VOL**	• No significant sex-by-PD interactions
Tremblay et al. 2020	Parkinson's Progression Markers Initiative (PPMI)	232 PD; 117 Control	35.8% PD; 33.3% Control	61.0 ± 9.0 PD M; 60.0 ± 9.0 PD F	> 30	Cross-Sectional	sMRI / DBM	**VOL**	• **In PD:** smaller cortical GM VOL in females than males (adjusting for ICV)<br>• **In PD:** larger CSF VOL in females than males (adjusting for ICV)
								**CTh**	• No significant sex effects in global or regional CTh
								**GM VOL**	• **In PD:** smaller GM VOL in males than females in BL MFG, L IFG, R precentral gyrus, L insular lobe, L medial prefrontal, thalamus, L ITG, R postcentral gyrus<br>• **In PD:** smaller GM VOL in females than males in superior parietal, R occipital lobe and three regions of frontal cortex<br>• DBM measures were not associated with clinical or cognitive outcomes in males or females
								**Local network efficiency** (structural connectivity)	• **In PD:** lower local network efficiency (in a structural connectivity analysis) in males than females in ~45% of 246 ROIs<br>• **In males:** lower local network efficiency in R IFG associated with lower scores on delayed recall on HVLT<br>• **In males:** lower efficiency in ventral agranular insula associated with lower scores on Letter Number Sequencing task

Table S1. Neuroimaging studies of sex effects in human brain structure

Burciu et al. 2017	PPMI	46 PD;<br>49 Control	26.1% PD;<br>24.5% Control	59.1± 9.7 PD;<br>60.3 ± 10.4 Control	NA	Longitudinal	dMRI	**FA**	• **In PD:** greater increase in free water corrected FA in males than females (over 4 years) in posterior substantia nigra
Multiple Sclerosis (MS)									
Jakimovski et al. 2020	University at Buffalo MS clinic	1,554 RRMS;<br>453 PPMS;<br>192 CIS	76.3% RRMS;<br>70.4% PPMS;<br>76.5% CIS	46.0 ± 11.6 MS	~18-85	Cross-Sectional	sMRI	**TBV**	• **In MS (aged 18-59 years) MS:** smaller normalized whole brain VOL (adjusted for head size using FSL's SEINAX) males than females
								**GM VOL**	• **In MS (aged 18-59 years) MS:** smaller GM VOL in males than females
								**ventricle VOL**	• **In MS (aged 40-59 years):** smaller lateral ventricle VOL in **females** than **males**
Voskuhl et al. 2020	VIMS Study	89 MS;<br>45 Control	68.0% RRMS;<br>65.0% SPMS;<br>50.0% PPMS	42.1 ± 12.4 MS F;<br>40.7 ± 11.7 MS M;<br>38.2 ± 12.5 Control F;<br>37.2 ± 15.7 Control M	18-69	Cross-Sectional	sMRI / VBM	**GM VOL**	• **In males:** smaller GM VOL in MS than controls in thalamus, putamen, precuneus, and medial PFC<br>• **In females:** smaller GM VOL in MS than controls in thalamus
								**CTh**	• **In males only:** lower CTh in MS than controls, localized to R intraparietal sulcus<br>• **In females:** no case-control differences
								**WM VOL**	• **In females:** smaller global WM VOL in **MS** than **controls**<br>• **In males:** no case-control differences
Eshaghi et al. 2019	15 RCT, 3 observational cohorts	2,884 RRMS;<br>1,837 SPMS;<br>1,601 PPMS	68.0% RRMS;<br>65.0% SPMS;<br>50.0% PPMS	37.4 ± 9.2 RRMS;<br>49.4 ± 8.1 SPMS;<br>49.2 ± 8.4 PPMS	NA	Longitudinal	sMRI	**T1/T2 MRI ratio**	• A higher proportion of females than males have early reduction in T1/T2 MRI ratio in normal appearing WM in cingulum and CC, followed by subcortical GM atophy in frontal, temporal, and parietal cortices
Klistorner et al. 2018	University of Sydney/ Macquarie University	43 RRMS;<br>20 Control	55.8% RRMS;<br>60.0% Control	42.1 ± 6.1 RRMS;<br>41.0 ± 9.1 Control	NA	Longitudinal	dMRI	**MD**	• **In MS:** larger increase in MD in males than females in lesion core (approx 42 months later)

Table S1. Neuroimaging studies of sex effects in human brain structure

Sanchis-Segura et al. 2016	Hospital General of Castellón	56 RRMS;<br>63 Control	60.7% RRMS;<br>44.4% Control	38.7 ± 8.7 RRMS M;<br>40.9 ± 10.2 RRMS F;<br>25.5 ± 5.4 Control M;<br>28.0 ± 7.9 Control F	18-60	Cross-Sectional	sMRI / VBM	**GM VOL**	• **In females:** smaller GM VOL in MS than controls in BL thalamus, L calcarine sulcus, and L precuneus<br>• **In males:** smaller GM VOL in MS than controls in BL thalamus L calcarine sulcus, L precuneus, L lingual gyrus and L cerebellum<br>• **In MS:** larger GM VOL in females than males in frontal lobe
Schoonheim et al. 2014	VUMC Amsterdam	114 RRMS;<br>9 SPMS;<br>8 PPMS;<br>49 Control	69.3% RRMS;<br>66.7% SPMS;<br>25.0% PPMS;<br>59.2% Control	42.1± 9.6 MS M;<br>40.5 ± 9.2 MS F;<br>39.6 ± 11.6 Control M;<br>41.0 ± 11.2 Control F	NA	Cross-Sectional	dMRI	**FA, MD, RD, AxD**	• Both male and female MS patients had lower FA, and higher MD, RD, and AxD than male and female controls in CC, temporal WM and posterior periventricular regions (adjusting for age and education)<br>• **In males:** lower FA and higher diffusivity in MS than controls in posterior CC, thalamus, cerebellum, pons, and fronto-parietal WM<br>• Case-control differences in FA, MD, RD, and AxD were significantly larger (i.e. greater severity) in **males** than **females**<br>• MS males had more abnormal voxels (i.e. severity extent) of FA, MD, AxD, RD than MS females
Rojas et al. 2013	Hospital Italiano de Buenos Aires	45 RRMS	55.6% RRMS	34.2 ± 1.1 RRMS M;<br>33.5 ± 1.6 RRMS F	NA	Cross-Sectional	sMRI	**TBV, GM, WM, lesion VOL**	• **No sex effects** in baseline MRI assessment of TBV, GM VOL, WM VOL, and lesion VOL
						Longitudinal		**TBV, GM VOL**	• **In MS:** smaller decrease in TBV and GM VOL in females than males at 6 year follow-up visit
								**lesion VOL**	• **In MS:** smaller increase in lesion VOL in females than males at 6 year follow-up visit
								**VOL** (atrophy)	• **In MS:** more localized atrophy in females than males in subcortical frontal lobe areas<br>• **In MS:** more diffuse atrophy pattern in males than females

Table S1. Neuroimaging studies of sex effects in human brain structure

Schoonheim et al. 2012	VUMC Amsterdam	120 RRMS;<br>50 Control	66.7% RRMS;<br>60.0% Control	40.4 ± 9.0 RRMS M;<br>39.6 ± 8.3 RRMS F;<br>39.6 ± 11.6 Control M;<br>40.9 ± 11.0 Control F	NA	Cross-Sectional	sMRI	**WM VOL**	• **In males:** smaller normalized WM VOL in MS than controls<br>• **In females:** smaller normalized WM VOL in MS than controls
								**cortical VOL**	• **In males:** smaller subcortical GM VOL in MS than controls in BL thalamus, BL caudate, BL putamen, BL pallidum, BL amygdala, and L nucleus accumbens (strongest effect in L caudate)
								**subcortical VOL**	• **In females:** smaller subcortical GM VOL in MS than controls in BL thalamus, BL caudate, BL pallidum, BL amygdala, and L nucleus accumbens (strongest effect in L pallidum)<br>• **In MS:** smaller subcortical GM VOL in males than females in BL caudate and R putamen
								**GM VOL**	• **In males:** smaller normalized GM VOL in MS than controls<br>• **In MS males:** smaller normalized GM VOL, normalized cortical GM VOL, and normalized deep GM VOL were associated with worse average cognition<br>• **In MS females**: normalized GM VOL, normalized cortical GM VOL, and normalized subcortical GM VOL were associated with worse average cognition
								**lesion VOL**	• **No sex effects** in T1 or T2 lesion VOL<br>• **In MS males**: larger lesion VOL (T1-w) associated with lower mean cognitive performance across 7 domains
Antulov et al. 2009	SUNY at Buffalo	499 RRMS;<br>230 SPMS;<br>34 PPMS;<br>32 CIS;<br>101 Control	80.8% RRMS;<br>75.2% SPMS;<br>55.9% PPMS;<br>78.1% CIS;<br>69.3% Control	43.8 ± 9.3 RRMS;<br>51.0 ± 9.7 SPMS;<br>52.6 ± 20.8 PPMS;<br>41.3 ± 9.2 CIS;<br>44 ± 10 Control	19-78	Cross-Sectional	sMRI	**GM VOL**	• **In MS:** smaller normalized GM VOL and normalized peripheral GM VOL in males than females
								**WM VOL**	• **In MS:** smaller normalized WM VOL and third ventricle width in females than males
								**ventricle VOL**	• **In MS:** smaller third ventricle width in females than males
Alzheimer's disease (AD)									

Table S1. Neuroimaging studies of sex effects in human brain structure

Burke et al. 2019	National Alzheimer's Coordinating Center (NACC)	211 MCI at baseline; 483 CN at baseline	50.2% MCI; 65.4% CN	76.76 MCI M; 77.5 MCI F; 72.98 CN M; 70.44 CN F	NA	Longitudinal	sMRI	**hippo VOL**	• **In CN females**: every 1% increase in hippo VOL associated with a 465% reduction in rate of progression to AD, and a 614% reduction in rate of progression to MCI (over a 10-year period)<br>• **In males:** hippo VOL not associated with an increased risk of cognitive decline (MCI or AD) (over a 10-year period)
								**WMH VOL**	• **In females**: WMH ratio (relative to TBV) not associated with an increased risk of cognitive decline (MCI or AD) (over a 10-year period)<br>• **In CN males**: larger WMH VOL (relative to TBV) associated with faster progression to MCI, with White males having an increased rate of progression to AD compared to males in other racial groups (over a 10-year period)
Lee et al. 2018	Alzheimer Disease and Positron Emission Tomography (ADAPET)	36 AD; 14 CN	61.1% AD; 50.0% CN	73.1 ± 5.6 AD M; 68.4 ± 8.8 AD F; 68.3 ± 9.1 CN M; 66.0 ± 6.2 CN F	NA	Longitudinal	sMRI	**CTh**	• **In AD:** faster cortical thinning in females than males in L DLPFC, L STG, BL temporo-parietal association cortices, BL ACC, BL middle PFC, and BL occipital cortices (over 5 years) (adjusting for age, education, *APOE*4 status, disease duration and onset, and ICV)<br>• **In CN:** no significant sex differences in total or regional CTh (over 5 years)
Caldwell et al. 2018	Alzheimer's Disease Neuroimaging Initiative (ADNI)	342 MCI; 178 CN	46.1% MCI; 52.2% CN	71.3 ± 6.9	55-89	Cross-Sectional	sMRI	**hippo subfield VOL**	• **In CN males:** Aβ+ associated with smaller R whole hippo VOL and subiculum VOL<br>• **In CN females:** no sex-by-Aβ+ interaction on hippo VOL or subiculum VOL<br>• **In MCI:** no sex effect on whole hippo VOL or subfield VOL<br>• **In MCI:** no sex-by-MCI-by-Aβ interactions on CA1, CA3 CA4, and dentate gyrus VOL<br>• **In MCI:** a sex-by-MCI interaction significant in R CA1, but post hoc tests were not reported

Table S1. Neuroimaging studies of sex effects in human brain structure

Koran et al. 2017	ADNI	185 AD;<br>565 MCI;<br>348 CN	46.0% AD;<br>42.0% MCI;<br>53.0% CN	74.0 ± 8.0 AD;<br>72.0 ± 8.0 MCI;<br>74.0 ± 6.0 CN	55-90	Cross-Sectional	sMRI	**hippo VOL**	• No significant sex-by-Aβ or sex-by-total tau interactions on baseline hippo VOL
						Longitudinal	sMRI	**hippo VOL** (atrophy)	• **In total sample**: faster rate of hippo atrophy (average over 2.5 years) in females with low CSF Aβ42 than males with low CSF Aβ42<br>• **In total sample**: faster rate of hippo atrophy (average over 2.5 years) in females with high CSF t-tau than males with high CSF t-tau<br>• **In total sample:** faster rate of hippo atrophy (average over 2.5 years) in females than males in presence of *APOE*4 genotype<br>• **In total sample:** faster rate of hippo atrophy (average over 2.5 years) in females with lower education than males with lower education
Kim et al. 2015	Clinical Research Center for Dementia of South Korea Study (CREDOS)	294 MCI	65.6% MCI	72.0 MCI (median)	60-91	Longitudinal	sMRI	**WMH** (visual rating severity)	• **In MCI males:** WMH predicted increased risk for disease progression<br>• **In MCI males:** severe periventricular WMH (>10mm VOL) at baseline predicted ~8-fold increased risk of progression from MCI to AD (over an average of 3 years) compared to those with mild-to-moderate periventricular WMH (5 mm–10 mm VOL)<br>• **In MCI males:** severe deep subcortical WMH associated with significantly decreased risk for AD progression<br>• **In MCI females:** WMH were not associated with disease progression to AD
Noh et al. 2014	Samsung Medical Center	152 AD;<br>72 CN	66.4% AD;<br>70.8% CN	71.8 ± 8.9 AD;<br>71.3 ± 5.8 CN	NA	Cross-Sectional	sMRI / vertexwise	**VOL** (atrophy)	• Higher proportion of females than males exhibited a dominant pattern of medial temporal/cingulate atrophy (731% female) or diffuse atrophy (ie, nearly all association cortices except occipital and OFC, 667% female)
O'Dwyer et al. 2012	Adelaide and Meath Hospital	33 MCI;<br>40 CN	63.6% MCI;<br>60.0% CN	71.3 ± 7.0 MCI M;<br>66.2 ± 6.5 MCI F;<br>68.3 ± 8.4 CN M;<br>64.1 ± 6.8 CN F	NA	Cross-Sectional	sMRI	**GM VOL**	• **In MCI:** no significant sex-by-MCI interaction on GM VOL (normalized by head size)
								**WM VOL**	• **In MCI:** No significant sex-by-MCI interaction on WM VOL (normalized by head size), and no main effect of sex on WM VOL
							dMRI	**FA**	• **In total sample:** higher FA in females than males, predominantly in splenium of CC
								**RD**	• **In total sample:** lower RD in females than males in BL SLF and ILF, splenium of CC, ATR, forceps minor, UNC
								**MD**	• **In total sample:** lower MD in females than males in R SLF, R ILF

Table S1. Neuroimaging studies of sex effects in human brain structure

Skup et al. 2011	ADNI	197 AD;<br>266 MCI;<br>224 CN	48.7% AD;<br>33.8% MCI;<br>49.1% CN	75.7 ± 7.7 AD;<br>74.9 ± 7.6 MCI;<br>76.0 ± 5.0 CN	55-90	Longitudinal	sMRI (VBM)	**VOL**<br>(atrophy)	• **In AD:** faster atrophy in females than males of BL thalamus, MTG, and L insula (over 2-3 years; adjusting for ICV, education, sex, age, age2, and diagnosis)<br>• **In amnestic MCI:** faster atrophy in females than males in BL caudate and thalamus<br>• **In amnestic MCI:** males had faster atrophy of BL precuneus and R ERC
Seo et al. 2011	Alzheimer Disease and Positron Emission Tomography (ADAPET) study	193 AD;<br>142 CN	69.4% AD;<br>60.6% CN	73.5 ± 7.3 AD;<br>66.0 ± 7.9 CN	NA	Cross-Sectional	sMRI	**CTh**	• **No sex differences** in regional CTh
Hua et al. 2010	ADNI	144 AD;<br>338 MCI;<br>202 CN	NA AD;<br>35.5% MCI;<br>NA CN	76.5 ± 7.4 AD;<br>76.0 ± 7.2 MCI;<br>77.0 ± 5.1 CN	NA	Longitudinal	sMRI /TBM	**cortical VOL**<br>(atrophy)	• **In all females** and **MCI females**, brain atrophy rates were ~1-15 times faster in whole brain and medial temporal lobe compared to all males and MCI males respectively (1 year follow-up)<br>• **In AD:** no significant sex effects
Salat et al. 2009	Alzheimer's Disease Research Center (ADRC)	91 AD;<br>73 Old CN;<br>135 Young CN	59.3% AD;<br>64.4% Old CN;<br>54.1% Young CN	77.6 ± 0.7 AD;<br>76.8 ± 0.8 Old CN;<br>26.5 ± 0.8 Young CN;	18-93	Cross- Sectional	sMRI	**WM VOL**<br>**cortical VOL**	• **In AD:** smaller WM VOL in females than males<br>• **In AD:** males had less VOL than females in L lateral OFG, SMG, R STG, R caudal MFG, R MTG, and R pars operculari
Vascular dementia (VAD)									
van Dijk et al. 2008	Rotterdam Scan Study	668 non-demented	0.52	71.0 ± 7.0	60-90	Longitudinal	sMRI	**lesion VOL**<br>(progression)	• Females had a greater likelihood of subcortical WM lesion progression (over 3 years) than males
								**lacunar infarcts**<br>(yes/no)	• Females had a greater likelihood of incident cerebral infarctions than males
De Leeuw et al. 2001	Rotterdam Scan Study	1,077 non-demented	52%	75.9	60-90	Cross-Sectional	sMRI	**subcortical WM lesions**<br>(incidence)	• Females had a greater incidence of subcortical WM lesions than males in frontal and periventricular regions<br>• Females had more large (upper quintile of their distribution) subcortical WM lesions that were large than males
Longstreth et al. 1998	Cardiovascular Health Study (CHS)	3,660 population-sample	NA	NA	> 65	Cross-Sectional	sMRI	**WM lacunes type**<br>(yes/no)	• Females had more silent and multiple WM lacunes than males
Frontotemporal lobe dementia (FTD)									
No studies evaluating neuroimaging sex differences were found.									
Lewy Body dementia (LBD)									

Table S1. Neuroimaging studies of sex effects in human brain structure

Ballmaier et al. 2004	University of California, Los Angeles (UCLA)	16 LBD;<br>29 AD;<br>38 Control	50.0% LBD;<br>44.8% AD;<br>52.6% Control	76.4 ± 6.7 LBD;<br>77.9 ± 5.5 AD;<br>75.3 ± 6.8 Control	> 60	Cross-Sectional	sMRI	**GM VOL** (cortical pattern matching)	• **In DLB:** less GM in males than females in frontal dorsal and parietal lobes, particularly in ACC

Systematic reviews were not included. Studies that do not include a standard deviation were not reported in the original publication. The table lists all cited references in the manuscript that discussed neuroimaging also did not include sex differences in clinical presentation or neuropathology. Structural indicates the analysis was done either using a T1-weighted, T2-weighted, or Proton Density scan. Diffusion indicates the ana was not reported for the combined cohort in the original publication and therefore we manually computed an estimate mean age based on averages ages reported in each cohort form the mega-analysis or meta-an inaccurate the subfields. Abbreviations: ACC = anterior cingulate cortex, AD = Alzheimer's disease, ADAPET = Alzheimer Disease and Positron Emission Tomography, ADNI = Alzheimer's Disease Neuroimaging In diffusivity, Aβ = amyloid-beta, BA = Brodmann areas, BD = Bipolar disorder, BL = bilateral, CA = cornu ammonis, CHS = Cardiovascular Health Study, CIS = Clinically isolated syndrome, CN = Cognitively Normal, C With Relapsing-Remitting Multiple Sclerosis, CREDOS = Clinical Research Center for Dementia of South Korea Study, CSF = Cerebrospinal Fluid, CST = Corticospinal tract, CTh = cortical thickness, DBM = Defor Tensor Imaging, ENIGMA = Enhancing Neuro Imaging Genetics Through Meta-analysis, ESPRIT = Enquête de Santé Psychologique – Risques, Incidence et Traitement or Psychological Health Survey – Risk, Incid Anisotropy, FDC = fiber density cross-section, GAD = Generalized Anxiety Disorder, GCDG = granule cell layer of dentate gyrus, GM = Gray matter, HATA = hippocampal-amygdala transition area, HCP = Human C fasciculus, ILF = inferior longitudinal fasciculus, IPL = inferior parietal lobe, lat = lateral, LBD = Lewy body dementia, M = Male, MCI = Mild Cognitive Impairment, MD = mean diffusivity, MDD = Major Depressive Dis OCD = Obsessive Compulsive Disorder, OFC = orbitofrontal cortex, PaD = Panic Disorder, PD = Parkinson's disease, PFC = prefrontal cortex, PPMI = Parkinson's Progression Markers Initiative, PPMS = Primary p Randomized controlled trial, RD = Radial diffusivity, RRMS = Relapsing remitting multiple sclerosis, SA = surface area, SCR = superior corona radiata, SCZ = Schizophrenia, SHIP = Study of Health in Pomerania, S progressive multiple sclerosis, STR = Super thalamic radiation, STS = superior temporal sulcus, SUD = Substance Use Disorder, TBM = Tissue based morphometry, TBSS = Tract Based Spatial Statistics, TBV = To volume, WM = White matter, WMH = White matter hyperintensity
